# Cancer-associated fibroblasts and their prognostic role in colorectal cancer: review and meta-analysis

**DOI:** 10.3389/fonc.2025.1635055

**Published:** 2025-12-10

**Authors:** Silvia Mihaela Ilie, Laurentia Gales, Ana Maria Musina, Kevin Rouet, Alexandra Elena Stefan, Mara Simona Belceanu, Anda Stefania Trandafir, Constantin Guillaume, Aurélie Bertaut, Ikram Charifi, Adel Cueff, Valentin Derangère, Francois Ghiringhelli

**Affiliations:** 1 Department of Medical Oncology, Centre Georges François Leclerc, Dijon, France; 2 Universite Bourgogne Europe, Center for Translational and Molecular Medicine, INSERM U1231, Dijon, France; 3 Department of Medical Oncology, Medical Oncology, Institute of Oncology Alexandru Trestioreanu, Bucharest, Romania; 4 Department of Medical Oncology, Carol Davila University of Medicine and Pharmacy, Bucharest, Romania; 5 Department of Medical Oncology, Regional Institute of Oncology, Iasi, Romania

**Keywords:** cancer-associated fibroblasts, colorectal cancer, prognostic biomarkers, tumor microenvironment, meta-analysis

## Abstract

**Background:**

Cancer-associated fibroblasts (CAFs) are a component of the tumor microenvironment, influencing tumor behavior and progression. This meta-analysis aims to describe the prognostic value of CAF biomarkers in colorectal cancer (CRC).

**Methods:**

A systematic search of the existing literature was performed in Medline and Embase on CAF immunohistochemical (IHC) biomarkers and their association with disease-free survival (DFS) and overall survival (OS). The selection of studies was conducted following the Preferred Reporting Items for Systematic Reviews and Meta-Analyses guidelines and Population, Intervention, Comparator, Outcome criteria. The quality of the included articles was assessed using the Newcastle–Ottawa Scale.

**Results:**

A total of 3,535 records were identified, of which 84 were selected for the qualitative review and 59 (*N* = 15,396 patients) were included in the final meta-analysis. In general, CAF biomarker expression was associated with poor prognosis, with high heterogeneity among studies. Significant results for DFS were found for cluster of differentiation (CD) 163, matrix metalloproteinase 9 (MMP-9), and tenascin C. The following markers were significantly associated with OS: CD163, MMP-9, periostin, and vimentin.

**Conclusion:**

Several CAF IHC biomarkers, particularly CD163, MMP-9, periostin, and vimentin, significantly associated with prognosis in CRC, could be proposed as surrogates for the phenotypical characterization of the tumor microenvironment.

## 1 Background

Colorectal cancer (CRC) is the third most common cancer worldwide in terms of incidence and the second in terms of mortality (1). The 5-year survival is approximately 60%, and in the metastatic stage, it drops to 14% (2, 3). The liver is the organ most frequently affected by CRC metastases (4), for which the optimal management is surgical resection when feasible (5). There are several validated prognostic factors in liver metastatic colorectal cancer (mCRC), such as synchronous disease, number of metastases, and tumor size (4). Lately, there has been an increasing interest in the tumor microenvironment (TME) and its impact on disease recurrence and survival (6). One of the main components of the TME is cancer-associated fibroblasts (CAFs), a heterogeneous population that originates from the local fibroblasts, pericytes, and bone marrow-derived mesenchymal cells (7). Normally, they are involved in the wound-healing process, but through proximity and crosstalk with tumor cells, they may take on either protumor activity, promoting cellular migration, pro-angiogenic cytokine synthesis, such as vascular endothelial growth factor A, and inflammation and plasticity of cancer stem cells (8); or a tumor-suppressive role, via the nuclear factor kappa-light-chain-enhancer of activated B-cell pathway (9).

CAFs in all tumors, including colorectal cancer, have mostly been assessed through next-generation sequencing (NGS); however, knowledge regarding their role is still controversial, and none of their characteristic biomarkers are commonly used as prognostic or predictive biomarkers in daily practice (10). In the literature, there are studies reporting certain CAF biomarkers as indicators of poor prognosis, such as vimentin (VIM), related to epithelial–mesenchymal transition (EMT) (11), and C-X-C motif chemokine ligand 12 (CXCL12), which has an immunosuppressive role on cluster of differentiation (CD)8 lymphocytes (12). Others, for example, podoplanin (PDPN), have been associated with improved prognosis in CRC, with PDPN-positive CAFs being related to decreased depth of tumor invasion, and their location is in the tumor center rather than at the invasion front (13).

CAFs are very heterogeneous populations with varying functional and prognostic value. However, to date, there is no universally accepted classification. Based on expression of interleukin 6 (IL-6), a protumoral cytokine, and alpha smooth muscle actin (α-SMA), a marker of myofibroblasts, a distinction into two classes has been proposed in preclinical models, namely, inflammatory CAFs (iCAFs), with an α-SMA^low^ IL-6^high^ profile, and myofibroblastic CAFs (myCAFs), with an α-SMA^high^ IL-6^low^ profile (14). Another proposed classification describes two CAF populations, namely, extracellular matrix-related (ECM)-CAFs, marked by fibroblast activation protein (FAP) and platelet-derived growth factor receptor (PDGFR)-β, involved in collagen deposition and immunomodulation; and contractile CAFs, expressing markers such as myosin heavy chain 11, regulator of G-protein signaling 5, and melanoma cell adhesion molecule, characterized by enhanced contractility (15). Another publication classified CAFs into CAF A cells, with high expression of proteins with a role in ECM remodeling [like matrix metalloproteinase 2 (MMP-2), decorin (DCN), and collagen type I alpha 2 chain (COL1A2)], and CAF B cells, with expression of genes of activated myofibroblasts [transgelin (TAGLN) and platelet-derived growth factor subunit A (PDGFA)] (16).

Although it is known that CAFs play a role in shaping the TME and influencing tumor behavior, individual studies often report conflicting or inconclusive results concerning their prognostic significance due to the heterogeneity of data (17, 18).

In this context, we aimed to identify CAF biomarkers, assessed through common laboratory methods such as immunohistochemistry, expressed in CRC, at all stages, and to describe their prognostic role in terms of either relapse-free survival or general or disease-specific survival, as long-term patient outcomes reported in the literature.

## 2 Materials and methods

### 2.1 Search strategy

A systematic literature search was carried out in the Medline and Embase databases for articles related to CAF-related biomarkers published from inception until December 2024.

To enhance coverage and minimize publication bias, we also performed supplementary searches using Google Scholar. Due to budgetary limitations, we were unable to include subscription-based databases such as Web of Science and Cochrane CENTRAL. The search strategy followed the Preferred Reporting Items for Systematic Reviews and Meta-Analyses (PRISMA) guidelines (19) and the Population, Intervention, Comparator, Outcome PICO criteria (20) and included MeSH/Emtree terms “OR” free terms for each CAF biomarker name “AND” MeSH/Emtree “OR” free terms for “colorectal cancer” “AND” MeSH/Emtree terms “OR” free terms for “prognosis” or “survival.” We refined the search using filters such as English, Humans, and Adult (**Supplementary Tables 1**, **2**). This was completed by a manual search of the reference lists for any relevant publications.

### 2.2 Study eligibility

Three investigators independently screened the titles and abstracts of all articles identified during the literature search. The full text of potentially relevant articles was obtained for further review according to predefined inclusion and exclusion criteria.

The types of studies included were retrospective, retrospective-prospective, or prospective and exploratory analysis of clinical trials. The population studied included patients diagnosed with histologically confirmed CRC, and the intervention was antigen determination of specific CAF biomarkers in the primary tumor or liver CRC metastasis specimens. We included studies reporting at least one of the following outcomes related to CAFs: DFS, recurrence/relapse-free survival (RFS), OS, or cancer-specific survival (CSS).

Only articles in English with the full text available and non-duplicate publications, providing hazard ratios (HRs) with 95% confidence intervals (CIs), were included.

The exclusion criteria were assessment of CAF biomarkers in preclinical models, insufficient data to estimate HRs or missing values (such as cutoff values for positivity), CAF assessment in body fluids or other than in the primary tumor or liver metastasis of CRC, measurement through gene expression or bioinformatics databases, reviews, or meta-analyses.

Quality assessment of the included articles was performed using the Newcastle–Ottawa Scale (NOS) (21) in line with the Cochrane Non-Randomized Studies Methods Working Group recommendations, rating studies on a scale from 0 to 9. Studies meeting five or more NOS criteria were considered eligible. Risk of bias was assessed using the QUADAS-2 scale (22). Funnel plots were constructed for studies reporting overall OS and DFS/RFS (**Supplementary Figure 1**).

### 2.3 Data extraction

Data extraction was performed by two researchers, and any disagreement was resolved through consensus with an independent researcher. Data from each study were summarized in an electronic data extraction form. The data extracted for each study included publication details in Vancouver style, study design, study objectives, participant characteristics, and study-specific particularities. In the final meta-analysis, only articles with multivariate analysis of survival were included to eliminate potential bias. For articles that used high versus low expression of biomarkers to calculate HR, the first or corresponding authors were contacted to ask for the data needed to recalculate it, and if no response was received after reminders, the article was excluded from the final statistical analysis. Articles that reported a different HR value for various methods of quantification for a specific biomarker, or different cohorts, were reported separately in this analysis and designated by a lowercase letter (a, b, c, etc.).

### 2.4 Statistical analysis

Statistical analysis was performed using SAS version 9.4 (SAS Institute Inc., Cary, NC), and a *p*-value <0.05 was considered statistically significant. To estimate the variance (Tau²) between studies, we applied a random-effects model, using restricted maximum likelihood (REML), as this method is more reliable than the DerSimonian and Laird method. We used Cochran’s *Q* test to assess global heterogeneity and *I*² to quantify total heterogeneity in variance between studies. Only studies with multivariate analysis were included in our survival analysis to obtain the pooled effect, to eliminate potential bias. Outcomes reported by individual studies, such as cancer-specific survival and OS, were assimilated to OS, and progression-free survival or relapse-free survival were regrouped under DFS.

## 3 Results

The literature search identified a total of 3,535 records, including 1,034 duplicates, for a total of 16 CAF biomarkers in CRC, namely, CD163, CXCL12, matrix metalloproteinase 9 (MMP-9), MMP-2, periostin (POSTN), calcium-binding protein S100A4 (S100A4), tenascin C (TNC), vimentin (VIM), transgelin 2 (TAGLN2), platelet-derived growth factor subunit α/β (PDGFR-α/β), PDPN, α-SMA, alpha-fibroblast activation protein (αFAP), collagen type I alpha 1 chain (COL1A1), collagen type XI alpha 1 chain (COL11A1), and decorin (DCN).

**Figure 1:**
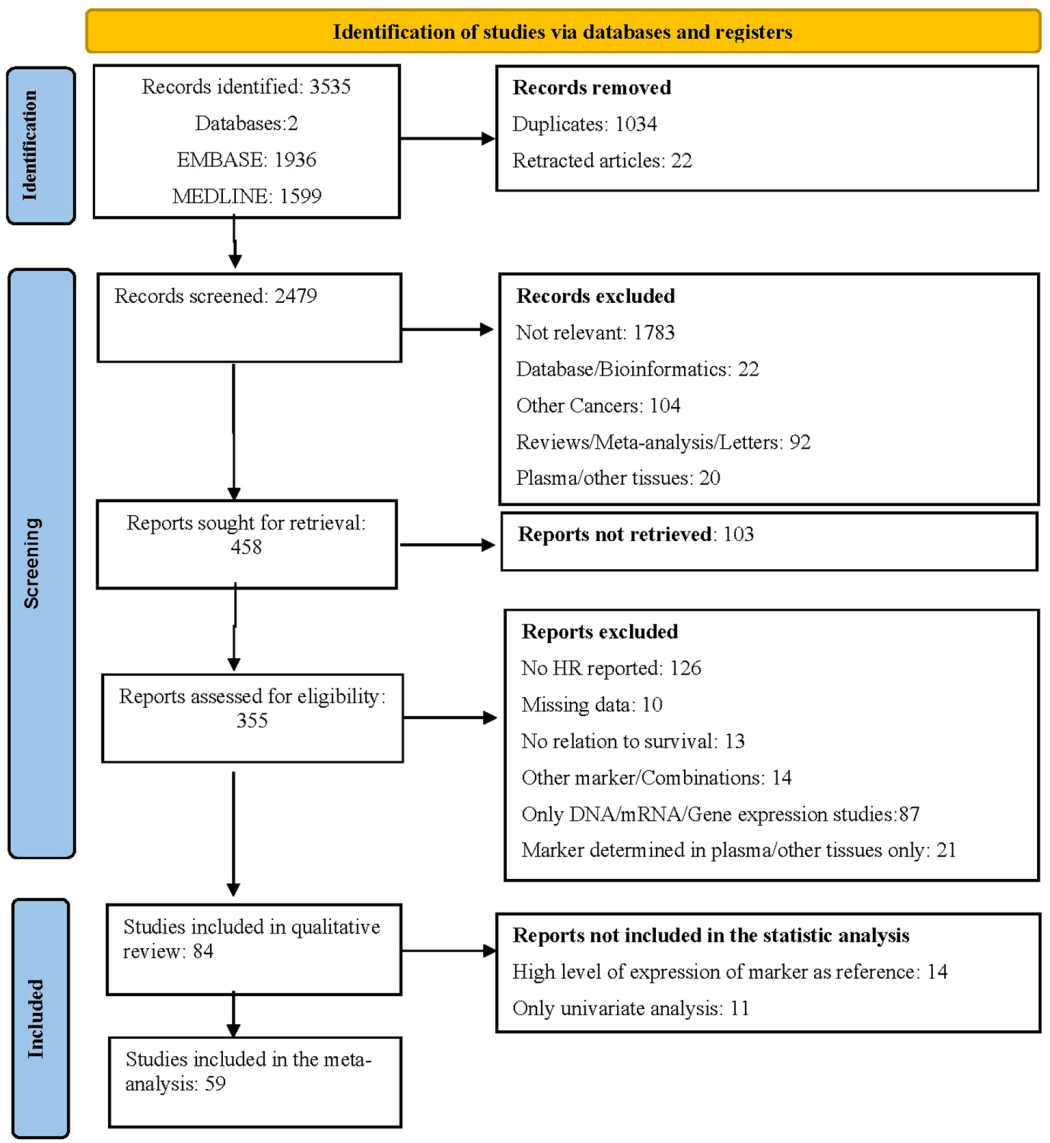
PRISMA flow diagram describing the selection of articles for inclusion in the meta-analysis.

After screening of the titles and abstracts, 2,021 records were excluded, and a further 458 were reviewed for the full text. After the full text was retrieved, 84 publications comprising 21,018 patients and 13 biomarkers were included in the review. In the meta-analysis, we included 59 articles, totaling 15,396 patients, all reporting HRs for survival from multivariate analysis (**Figure 1**).

### 3.1 Study characteristics

The characteristics of the articles and patients included are presented in **Supplementary Table 3**. Among the 59 studies included, NOS scores ranged from 5 to 8, and interrater agreement had a Cohen’s *κ* of 0.78. Most studies were from Asian countries (63/85 articles), accounting for 12,791 patients (60%). The size of the cohorts varied between 21 (23) and 1,125 (24), with 83% (70/84) of the studies including over 100 patients each. The reported mean or median age ranged from 52 to 73 years. All studies used IHC for biomarker detection, except three, which used ELISA on the primary tumor. In 10 articles, the survival outcomes associated with reported biomarkers were adjusted for known risk factors such as age, TNM stage, histology, and lymphovascular invasion. Regarding quality assessment, except for six studies with a quality score of 5/9, all the remaining studies were considered of good quality, with scores of 6 or higher (**Supplementary Table 3**).

Regarding the reports included only in the qualitative review, significant results were found for several markers. Deng et al. found that negative or low expression of MMP-2 was associated with a 27% lower risk of death (25), while Zhou et al. reported a 60% lower risk (26). Two studies regarding TAGLN2 (27, 28) respectively found a twofold increase and a 15% increase in the risk of death for low expression or absence of the marker. The study by Stanisavljević showed an increased risk of recurrence with low/negative expression of cytoplasm CXCL12 in two cohorts of stage I–III CRC, with significant results in the multivariate analysis with a hazard ratio (HR) of 1.61 [(0.95–2.73), *p* = 0.075] in one cohort and an HR of 5.13 [(2.23–11.81), *p* < 0.001] in the other group (29) (**Supplementary Tables 4**-**16**).

### 3.2 Disease-free survival

**Figure 2:**
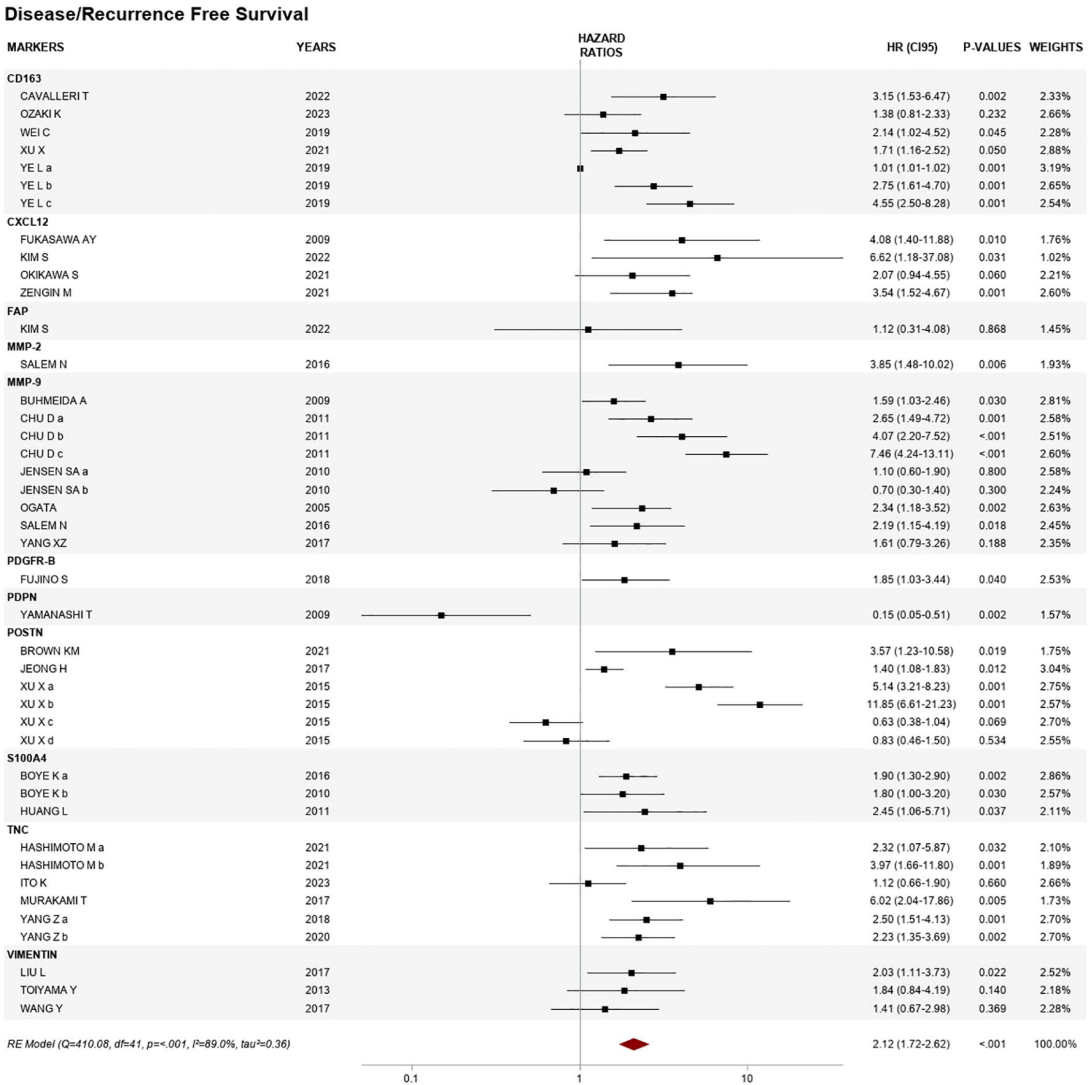
Forest plot of the association between cancer-associated fibroblast (CAF) biomarker expression and disease/recurrence-free survival.

**Figure 3:**
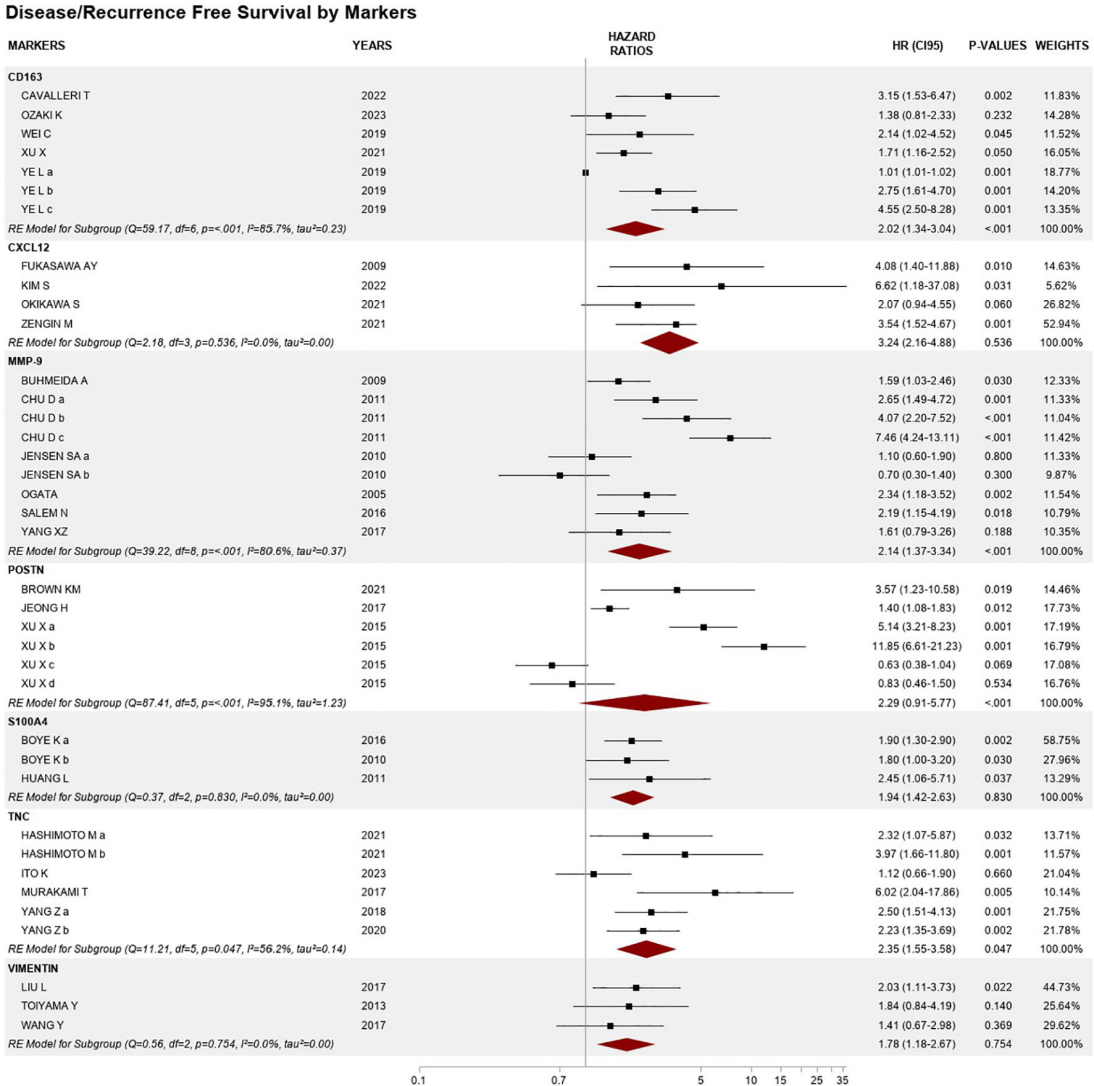
Forest plots of disease/recurrence-free survival by individual CAF biomarkers, based on studies with two or more comparisons.

Thirty articles reported HR for DFS/RFS, for a total of 11 CAF biomarkers: CD163, CXCL12, FAP, MMP-2, MMP-9, PDGFR-β, PDPN, POSTN, S100A4, TNC, and VIM. The overall pooled HR was 2.12 (1.72–2.62), *p* < 0.001, with *I*^2^ of 89.0%, *p* < 0.001, and between-studies variance tau^2^ = 0.47 (**Figure 2**). Regarding the analysis of individual biomarkers, overall hazard ratios and heterogeneity results were obtained for seven biomarkers (**Figure 3**).

For the five studies that assessed CD163, the overall HR was 2.02 (95% CI 1.34–3.04), *p* < 0.01, *I*^2^ 87.1%, and variance value of 0.23, of which three showed an association with shorter DFS. Ye and coworkers assessed CD163 in three different cohorts: a test cohort (*N* = 359), a training cohort (*N* = 249), and a validation cohort (*N* = 400), with significant HRs for the latter two: 2.75 (1.61–4.70) and 4.55 (2.50–8.28), respectively (30).

The overall HR from four articles reporting CXCL12 included in the analysis was 3.24 (95% CI 0.84–7.77) = 0.536, *I*^2^ = 0.0%, tau^2^ = 0.00, of which three studies reported a significant association.

MMP-9 expression showed associations with shorter DFS/RFS, with an overall HR from nine reports in six publications of 2.14 (95% CI 1.37–3.34), *p* < 0.01, with heterogeneity of 80.6%, *p* < 0.01, and variance of 0.37. Four studies reported HRs between 1.59 and 7.46, and Chu et al. assessed the biomarker in stages II–IV (*N* = 192) for three different cutoffs: a = weak, b = moderate, and c = strong expression (31).

Three articles were included for POSTN. Among these, Xu et al. reported results related to the expression in two different compartments, namely, stromal and intratumoral, with different cutoffs: medium (score = 6–8) and high (score = 9–12). Stromal expression was associated with a very high risk of relapse in localized disease with HRs of 5.4 and 11.85 (32). The pooled analysis for POSTN found an HR of 2.55 (95% CI 0.84–7.77), *p* < 0.05, with high heterogeneity and variance between studies (*I*^2^ = 95.1%, tau^2^ = 1.23).

Four studies included in the meta-analysis assessed TNC through a scoring system. The pooled HR was 2.35 (95% CI 1.55–3.58), *p* = 0.047, *I*^2^ = 56.2%, tau^2^ = 0.14, and all showed a significant HR (range from 2.23 to 6.02), except the study by Ito et al. (*N* = 259) on localized disease, who used a qualitative assessment based on the intensity of stained cells (33).

Two biomarkers were represented by three reports each: S100A4 with a pooled HR of 1.94 (95% CI 1.42–2.63) and VIM with a pooled HR of 1.78 (95% CI 1.18–2.67), both with low heterogeneity and variance between studies (*I*^2^ = 0.00%, tau^2^ = 0.00).

### 3.3 Overall survival

**Figure 4:**
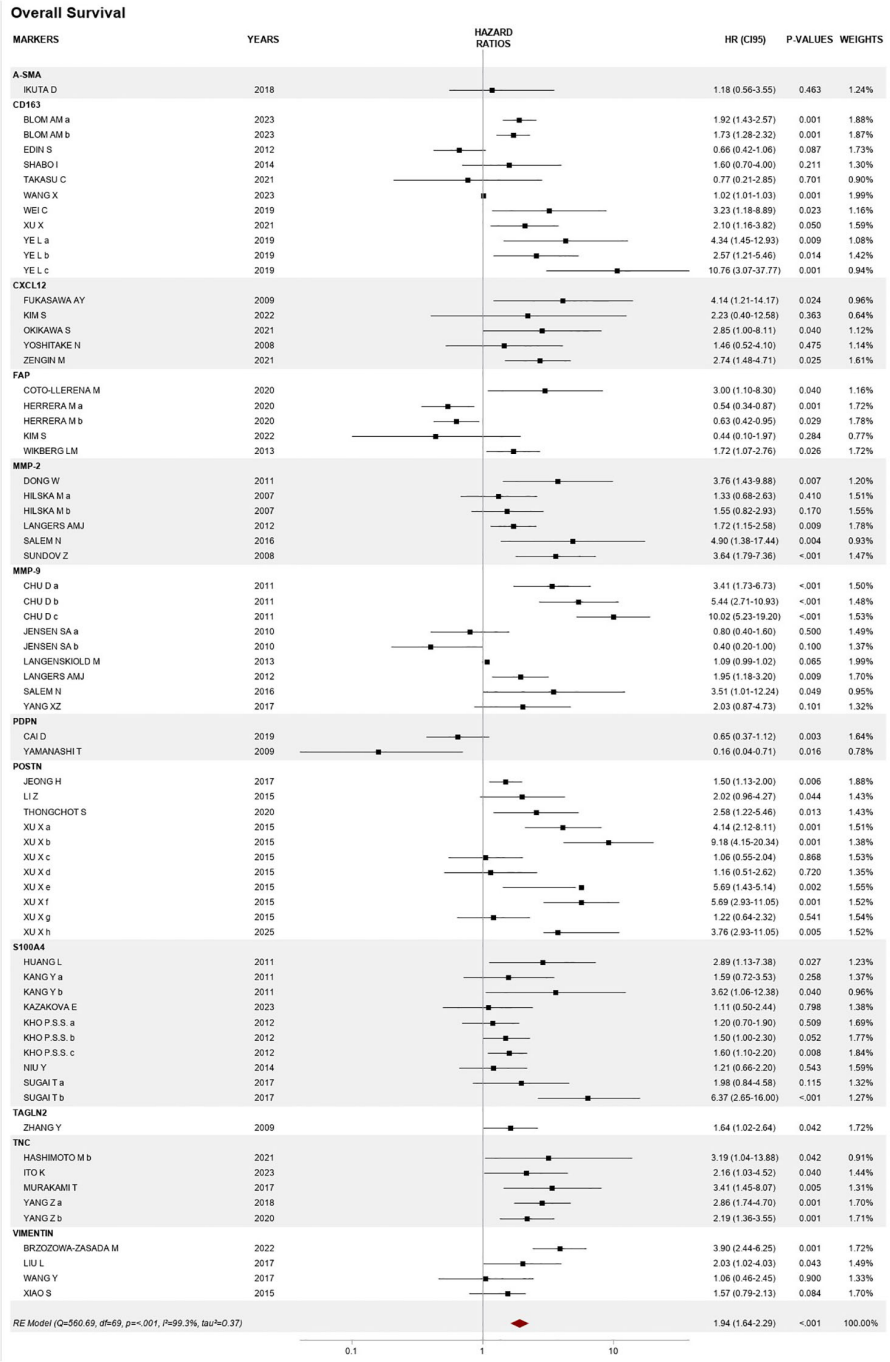
Forest plot of the association between CAF biomarker expression and overall survival.

Forty-eight publications assessing OS for 12 biomarkers were included in the meta-analysis: CD163, CXCL12, FAP, MMP-2, MMP-9, PDPN, POSTN, S100A4, TNC, TAGLN 2, VIM, and α-SMA. The overall HR was 1.94 (95% CI 1.64–2.29), *p* < 0.01, with high heterogeneity between studies, *I*^2^ value of 99.3%, *p* < 0.01, and high variance between studies, tau^2^ = 0.28 (**Figure 4**).

Among the individual markers, the results of our meta-analysis show a strong association with OS for CD163, MMP-9, POSTN, and VIM (**Supplementary Figure 2**).

Of the six publications about MMP-9, Chu et al. presented results using three different cutoffs (weak, moderate, and strong expression) (31), while Jensen et al. reported two areas of assessment, namely, intratumoral and the invasion front (34). Four studies showed consistent results, reporting HRs >1, ranging from 1.95 (95% CI 1.18–3.20) to 10.02 (95% CI 5.23–19.20). The overall HR was 2.10 (95% CI 1.09–4.04), *p* < 0.001, with high heterogeneity and high variance between studies (*I*^2^ = 93.1%, tau^2^ = 0.87).

The four studies reporting VIM showed a pooled HR of 2.01 (95% CI 1.16–3.46), *p* = 0.015, *I*^2^ = 69.5%, tau^2^ = 0.21, of which two showed significant results, namely, Brzozowa-Zasada et al. on localized disease (*N* = 97), with an HR of 3.90 (95% CI 2.44–6.25), *p* = 0.001 (35), and Liu et al. on stage II CRC (*N* = 203), HR 2.03 (95% CI 1.02–4.03), *p* = 0.043 (36).

CD163 was reported from 10 cohorts in eight articles. Blom et al. (*N* = 537) used two different areas of quantification, namely, intratumoral and in the surrounding stroma (37), while Ye et al. tested three cohorts, namely, a training cohort (*N* = 359), a test cohort (*N* = 249), and a validation cohort (*N* = 400) (30). Overall, CD163 was associated with an almost twofold increase in the risk of death, with an HR of 1.82 (95% CI 1.22–2.70), *p* < 0.001, *I*^2^ = 89.7%, and tau^2^ = 0.31.

Results from the four studies of POSTN found an *I*^2^ value = 81.5% and tau^2^ = 0.42. One study, on stage I–III disease, included two different cohorts, one from Shanghai (*N* = 682) and one from Guangzhou (*N* = 343), and assessed intratumoral expression and stromal expression separately for them both, using different cutoffs, namely, moderate (score = 6–8) and high (score = 9–12) (32). The pooled HR for OS of POSTN studies was 2.66 (95% CI 1.72–4.11), *p* < 0.001.

Publications analyzing the association between FAP and survival retrieved inconsistent results, with an HR of 0.44 (95% CI 0.10–1.97), *p* = 0.363, in the study by Kim et al. involving a localized cohort (*N* = 121) using qualitative assessment based on intensity of staining (38), and an HR of 3.00 (95% CI 1.10–8.30), *p* = 0.040, in the study by Coto-Llerena (*N* = 92), in stages I–IV, with semiquantitative assessment based on the percentage of positive staining (39). This resulted in a pooled HR of almost 1 (0.96, 95% CI 0.10–1.97) with high heterogeneity between the four studies included (*I*^2^ value = 82.9% and tau^2^ = 0.44).

The pooled effect of studies of TNC was 2.56 (95% CI 1.92–3.41), *p* = 0.855, from four studies reporting five cohorts (*I*^2^ = 0.0%, tau^2^ = 0.00, *p* = 0.855).

Six publications studied S100A4, of which Kang (*N* = 526) presented results for nuclear and cytoplasmic expression (40), while Kho et al. (*N* = 404) assessed staining at the invasion front, both cytoplasmic (Kho a) and nuclear expression (Kho c), and in the center of the tumor (Kho b) (41). Finally, Sugai (*N* = 106) reported values for intratumoral expression and stromal staining (42). Significant results were reported by Huang (43), Kang (40), and Kho (41), with HRs ranging from 1.60 (95% CI 1.10–2.20), *p* = 0.008, to 6.37 (95% CI 2.65–16.00), *p* = 0.076, reported by Sugai (42). The pooled HR was 1.68 (95% CI 1.32–2.15), *p* = 0.076, and heterogeneity was *I*^2^ = 31.0%, tau^2^ = 0.04, *p* = 0.076.

Five studies were included in the meta-analysis for CXCL12 expression, resulting in a pooled HR of 2.58 (95% CI 1.71–3.90), with *p* = 0.766, *I*^2^ = 0.0%, tau^2^ = 0.00, *p* < 0.001. Significant results were reported by Fukasawa (*N* = 165) with HR = 4.14 (95% CI 1.21–14.17), *p* = 0.024 (44); Okikawa (*N* = 98) with HR = 2.85 (95% CI 1.00–8.11), *p* = 0.040 (45); and Zengin (*N* = 260) with HR = 2.58 (95% CI 1.71–3.90), *p* = 0.025 (46).

From two studies including a total of 284 patients, positive PDPN was associated with a 60% reduction in the risk of death, *p* = 0.084. Yamanashi (*N* = 120) reported an HR of 0.16 (95% CI 0.04–0.71), *p* = 0.016 (47), and Cai reported an HR of 0.65 (95% CI 0.37–1.12), *p* = 0.003 (48). Heterogeneity analysis found *I*^2^ = 66.5% and tau^2^ = 0.64.

## 4 Discussion

### 4.1 Summary of findings

#### 4.1.1 Descriptive features

Of the 84 articles retrieved, we identified 78 publications that analyzed CAFs in the primary tumor, 4 on liver metastases only, and 2 on both sites. Regarding the cohorts, we found 34 publications about patients with localized stage, and 50 that also included patients with metastatic disease. We based our decision to include both localized and liver metastatic CRC cases in our analysis on a report showing that the prognostic value of CAFs is the same regardless of whether the tissue of origin is the primary tumor or the metastasis (49).

#### 4.1.2 Most significant CAF biomarkers and their association with prognosis

The majority of biomarkers identified had a negative prognosis on average, with high CAF levels associated with an almost twofold higher risk of recurrence or progression. The biomarkers significantly associated with an increased risk of disease were CD163, MMP-9, and TNC, with POSTN showing a borderline trend. For the other biomarkers, the results were less consistent across studies, such as CXCL12 and S100A4, where the majority were significant but did not show overall risk association (**Table 1**).

**Table 1:** Characteristics of the included studies.

No.	Study	Country	Patients analyzed/all cohort	Age (years)	Follow-up (months)	Adjustment	Proteins assayed	Outcome	Quality score
1	Ikuta 2018 (60)	Japan	94/104	66^a^ (60–72)	59.9	NS	α-SMA	RFS	6/9
2	Hashimoto 2021 (59)	Japan	286/286	NS	NS	NS	α-SMA, TNC	OS, DFS	7/9
3	Fujino 2018 (23)	Japan	21/194	NS	45.36	NS	PDGFR‐β	OS, DFS	6/9
4	Mezheyeuski 2016 (83)	Sweden, Denmark, Norway, Belarus	311/572	18–75	NS	NS	PDGFR-β	OS	5/9
5	Oh 2017 (24)	Korea	1,125/1,135	62^a^; 20–90	69.8	NS	POSTN	OS, PFS	6/9
6	Li 2015 (86)	China	115/135	NS	61	NS	POSTN	OS	6/9
7	Thongchot 2020 (87)	Thailand	410/410	64^b^ (29–95)	NS	NS	POSTN	OS	6/9
8	Kazakova 2023 (88)	Russia	118/118	66.3^b^ ± 10.3	NS	Age, pTNM, LVI	S100A4	OS	5/9
9	Niu 2015 (89)	China	131/131	57.9^a^ (29–83)	56	NS	S100A4	OS	6/9
10	Kho 2012 (41)	Australia	451/451	NS	NS	Age, grade, pTNM, treatment	S100A4	OS	7/9
11	Boye 2016 (61)	Norway	783/1,290	73^a^ (29–94); 63 (28–75)	116.4; 91.2	NS	S100A4	RFS	8/9
12	Kang 2011	Korea	526/526	NS (30–85)	40.1	NS	S100A4	OS, RFS	6/9
13	Huang 2011 (43)	China	112/144	NS	NS	NS	S100A4	OS, RFS	6/9
14	Sugai 2017 (42)	Japan	106/106	NS	66	NS	S100A4	OS	6/9
15	Yamanashi 2009 (47)	Japan	120/120	60^a^ (31–86)	62.4	pTNM, LVI	PDPN	RFS, DFS	7/9
16	Zhang 2010 (80)	China	120/148	62.2^b^ (25–89)	53.3	NS	TAGLN2	OS	6/9
17	Xu 2016 (28)	China	192/192	NS	NS	NS	TAGLN2	OS, DFS	7/9
18	Zhao 2009 (27)	China	126/138	NS	NS	NS	TAGLN2	OS	7/9
19	Murakami 2017 (90)	Japan	139/139	NS	NS	NS	TNC	OS	6/9
20	Yun 2014 (73)	Korea	409/409	59^b^ (26–85)	NS	NS	Vim, α-SMA	OS, DFS	7/9
21	Wang 2017 (75)	China	102/102	NS	55	NS	Vim, E-cadherin	OS, DFS	7/9
22	Toiyama 2013 (77)	Japan	208/208	68^a^ (12–91)	50	NS	Vim	OS	7/9
23	Lau 2018 (91)	Australia	37/37	58.8^b^ (23–76)	39	NS	Vim	OS, RFS	6/9
24	Secinti 2022 (92)	Turkey	84/100	62.8^b^ ± 12.7	NS	NS	Vim	OS	6/9
25	Liu 2017 (36)	China	203/203	NS	NS	NS	Vim	CSS, DFS	7/9
26	Xiao 2015 (76)	China	105/105	52^b^ (19–85)	NS	NS	Vim	OS	7/9
27	Deng 2017 (25)	China	463/470	NS	60	NS	MMP2	OS	6/9
28	Unsal 2008 (93)	Turkey	60/60	NS	29.45	NS	MMP9, MMP2	OS, DFS	7/9
29	Salem 2016 (64)	Saudi Arabia	127/127	NS	NS	Age, grade, pN	MMP9, MMP2	OS, DFS	6/9
30	Peltonen 2020 (94)	Finland	111/111	62.8^a^ (35.5–80.4)	182,4	NS	MMP9, MMP2	OS, DFS	7/9
31	Šundov 2008 (95)	Croatia	152/152	62^b^ (39–79)	NS	NS	MMP2	OS	6/9
32	Dong 2011 (96)	China	172/172	56.5^a^ (23–83)	NS	NS	MMP2	OS	7/9
33	Hilska 2007 (97)	Turkey	351/351	NS	NS	NS	MMP2	OS	6/9
34	Langer 2008 (98)	Germany	215/215	NS	NS	NS	MMP9, MMP2	OS	7/9
35	Liu 2021 (99)	China	191/191	NS	NS	NS	CD163	OS, DFS	6/9
36	Ledys 2018 (100)	France	114/114	63^a^ (29–83)	34.8	NS	CD163	OS, PFS	7/9
37	Cavalleri 2022 (101)	Italy	165/236	NS	57.48	NS	CD163	DFS	7/9
38	Wikberg 2013 (102)	Sweden	449/488	NS	NS	MSI status	FAP	CSS	6/9
39	Fukasawa 2009 (43)	Japan	165/165	61.8^b^ (32–93)	61	NS	CXCL12	RFS, OS	7/9
40	D’Alterio 2012 (103)	Italy	68/68	NS	64	NS	CXCL12	RFS, CSS	7/9
41	Stanisavljević 2015	Norway	502/677	61.9^b^	NS	Age, gender, pTNM, treatment	CXCL12	DFS	7/9
42	Yoshitake 2008 (104)	Japan	60/60	63.8^b^	NS	NS	CXCL12	OS	6/9
43	Yang 2020 (105)	Japan	100/100	NS	112	NS	TNC	DFS OS	6/9
44	Yang 2018 (106)	Japan	100/154	NS	112	NS	TNC	OS DFS	6/9
45	Ito 2023 (33)	Japan	259/259	69^a^ (34–92)	1,825 days; 2,093 days	NS	TNC	DFS OS	5/9
46	Cai 2019 (48)	China	164/164	62^b^	60	NS	PDPN	RFS/EFS/DFS OS	6/9
47	Algars 2011 (107)	Finland	145/159	72.8^a^ (31.8–91.3)	66,2	NS	PDPN	RFS/EFS/DFS OS	7/9
48	Yang 2017 (108)	China	179/179	59.5^b^	60	NS	MMP9	PFS OS	6/9
49	Ogata 2005 (109)	Japan	307/307	61.9^b^/70.9	64/87	NS	MMP9	RFS/EFS/DFS	6/9
50	Langers 2012 (110)	Netherlands	198/198	NS	60	NS	MMP9	OS	7/9
51	Jensen 2010 (34)	Denmark	340/340	NS	73.2	NS	MMP9	RFS OS	6/9
52	Chu 2011 (111)	China	192/192	NS	56	Gender, age, grade, pTNM, LVI	MMP9	DFS OS	
53	Buhmeida 2009 (68)	Turkey	202/202	NS	>240	Gender, age, pTNM, site	MMP9	DFS	7/9
54	Araújo 2015 (112)	Brazil	180/180	63^b^	60	NS	MMP2, MMP9	OS	6/9
55	Wang 2019 (113)	China	443/470	NS	60	NS	MMP9	RFS/EFS/DFS	7/9
56	Bendardaf 2009 (114)	Turkey	359/359	NS	240	NS	MMP9	DFS	7/9
57	Langenskiöld 2013 (115)	Sweden	136/136	73^b^ (45–91)/81 (51–89)	65	NS	MMP2 MMP9	CSS	8/9
58	Zhou 2011 (116)	China	141/141	59^a^	59 (8–95)	NS	MMP2	DFS OS	7/9
59	Herrera 2020 (117)	Sweden	520/565	NS	100	Age, pTNM, treatment, gender, site	FAP	OS	7/9
60	Coto 2020 (38)	Switzerland	92/100	NS	72	NS	FAP	OS	5/9
61	Zengin 2021	Turkey	260/355	69^a^ (37–92)	60	NS	CXCL12	RFS OS	7/9
62	Okikawa 2021 (45)	Japan	98/98	NS	60	NS	CXCL12	DFS OS	6/9
63	Kim 2021	South Korea	121/121	NS	45	NS	CXCL12 FAP	RFS OS	6/9
64	Ye 2019 (30)	China	1,008/1,008	NS	60	NS	CD 163	DFS OS	7/9
65	Xu 2021 (118)	China	1,021/1,021	NS	58	NS	CD 163	DFS OS	6/9
66	Wang 2023 (119)	China	255/255	NS	150	NS	CD 163	OS	6/9
67	Wen 2020 (120)	Sweden	219/219	NS	60	NS	CD 163	DFS OS	
68	Wei 2019 (121)	China	81/81	NS	>60	NS	CD 163	RFS OS	6/9
69	Takasu 2021 (122)	Japan	71/71	66.9^b^ (32–90)	51.9	NS	CD 163	OS	6/9
70	Shin 2021 (123)	Korea	148/148	67.2^b^ (32–92)	>60	NS	CD 163	DFS OS	7/9
71	Shabo 2014 (124)	Sweden	75	NS	120	NS	CD 163	OS	6/9
72	Ozaki 2023 (125)	Japan	205/205	66^a^ (32–90)	60	NS	CD 163	RFS	7/9
73	Kitagawa 2022 (126)	Japan	275/287	NS	73	NS	CD 163	RFS OS	6/9
74	Edin 2012 (127)	Sweden	422/485	NS	>60	NS	CD 163	CSS	7/9
75	Blom 2023 (37)	Sweden	537/626	71^a^ (50–86)	NS	NS	CD 163	OS	7/9
76	Xue 2021 (128)	China	209/209	NS	>50	NS	CD 163	DFS, OS	6/9
77	Xu 2015	China	1,025/1,098	NS	60	pTNM, treatment	POSTN	DFS (A-B-C-D) DSS	7/9
78	Brown 2021 (129)	Australia	110/220	62.88^a^ (56.26, 72.24)	66/45	NS	POSTN FAP	DFS OS	7/9
79	Boye 2010 (130)	Norway	237/242	73^b^ (35–98)	NS	NS	S100A4	DFS OS	8/9
80	Kwak 2010 (131)	Korea	127/127	59.3^b^ (28–88)	58.7	NS	S100A4	OS	6/9
81	Zasada 2022 (34)	Poland	97	68 (33–89)	60	NS	VIM	OS	7/9
82	Akter 2022 (132)	Korea	399/399	NS	42.1	NS	CD163	RFS, OS	6/9
83	Ke 2023 (133)	China	45/232	NS	NS	NS	CD163	OS	5/9
84	Kanno 2020 (134)	Japan	117/117	70^a^	>60	NS	CD163	OS, DFS	6/9

α-SMA, alpha smooth muscle actin; CD, cluster of differentiation; CSS, cancer-specific survival; CXCL12, C-X-C motif chemokine ligand 12; DFS, disease-free survival; FAP, fibroblast activation protein α; LVI, lymphovascular invasion; MMP, matrix metalloproteinase; No., number; NS, not specified; MSI, microsatellite instability; OS, overall survival; PDGFR-β, platelet derived growth factor subunit B; PDPN, podoplanin; POSTN, periostin; pTNM, pathologic tumor node metastasis; RFS, relapse-free survival; S100A4, calcium binding protein A4; TAGLN 2, transgelin2; TNC, tenascin C; Vim, vimentin.

a Median age.

b Mean age.

While MMP-9, TNC, and POSTN support tumor progression through ECM remodeling, CD163 is involved in tumoral immune suppression (8).

Regarding the association with overall survival, the pooled effect was similar to that for DFS/RFS, and also included VIM, which is a marker of EMT.

As for PDPN, there were only two studies included in the meta-analysis, which showed a protective effect regarding the risk of death, but without statistical significance.

#### 4.1.3 Overall completeness and applicability of evidence

Although this is a topic that is attracting more and more attention, as evidenced by at least two meta-analyses related to specific biomarkers of CAF, i.e., S100A4 (50) and CXCL12 (51), the articles report a few robust outcomes, either due to a lack of comparator in the survival analyses, a lack of multivariate models, or lack of validation in external cohorts.

#### 4.1.4 Potential biases in the review process

In the review process, one potential source of bias was that we selected studies reported in articles, rather than those only reported in conference abstracts, with a view to including robust results in the analysis. The sources of bias for the selected publications were multiple covariates, coprimary endpoints, and a retrospective design.

### 4.2 Comparison with previous studies

Very few reviews specifically addressed the prognostic value of IHC biomarkers assessing CAFs, probably due to a lack of specificity regarding the normal fibroblasts (7). Single-cell sequencing has identified a significant number of specific biomarkers, but for now, these methods are not applicable in everyday practice (15, 52). Overall, in terms of either overall survival or survival without disease, CAFs were shown to carry a negative prognostic value.

One explanation might lie in their origin, as they are derived from common fibroblasts, one of the most versatile and resistant cells, through epigenetic alterations that lead them to a state of permanent hyperactivation (53). Therefore, CAFs acquire features important to tumor progression, such as migratory capacity, ECM, chemokines, and growth factors, such as synthesis of transforming growth factor−β (TGFβ), platelet-derived growth factors (PDGFs), epidermal growth factors (EGFs), and fibroblast growth factors (FGFs) (8, 54).

Among the IHC biomarkers expressed in colorectal tumors, FAP, α-SMA, VIM, S100A4, PDGFR-α/β, and POSTN were reported in a general review addressing the question of the significance of CAFs in tumor progression and metastasis (55). PDGFR-β and α-SMA were associated with angiogenesis (56), and PDPN and S100A4 with lymphatic metastasis (57). α-SMA and FAP were correlated with adverse outcomes, through lymphangiogenesis, treatment resistance, and immune suppression (55).

FAP is a type II integral membrane protein, with a role in fibrosis and tissue repair, not specific for CAF, and when present, has a role in ECM remodeling and angiogenesis (7).

In our analysis, the HR for OS in FAP-positive cases was 0.8 (95% CI 0.37–1.73, *p* = 0.022), while for the risk of recurrence or progression, only one study reported a non-significant unitary value. High FAP expression in the tumors of patients with CRC was associated with poor prognosis in a specific meta-analysis (HR 1.72, 95% CI: 1.58–9.48, *p* = 0.009), in 876 stage I–IV CRC patients (58).

α-SMA is a member of the actin family, with functions in cell motility and integrity, not specific to CAF, but it is a marker of CAF B, expressed in activated myofibroblasts (7). In our study, only two articles were found reporting the association between α-SMA and prognosis, with no significant results, probably due to the reduced number and overselection of cases. Hashimoto et al. studied two stage II–III cohorts, one for testing (*N* = 148) with an HR of 0.68, *p* = 0.546, for DFS and 0.63, *p* = 0.546 for OS, and a second for validation (*N* = 138) with an HR = 1.58 (95% CI 0.33–28.26), *p* = 0.630, for OS, in stages II–III, using Dako 1A4 antibody, a score based on intensity and percentage of stained cells and univariate analysis (59). Ikuta reported an HR of 1.18, *p* = 0.463, in stage III–IV CRC (*N* = 94), using a score based on the area of stained cells by another monoclonal antibody ab7817, in multivariate analysis (60).

In this review, S100A4 was consistently associated with significant HRs, for DFS/RFS in two studies and for OS in eight studies, but the overall association between S100A4 and survival outcomes was not statistically significant (HR = 1.66, *p* = 0.109; HR = 1.99, *p* = 0.591). S100A4 is a member of the S100 calcium-binding protein family localized in the nucleus, cytoplasm, and extracellular space and is also known as metastasin. It increases cell motility via interactions with the cytoskeleton and contributes to a more aggressive cell phenotype via p53 modulation and is not specific for CAFs (61).

S100A4 was assessed in a previous meta-analysis, published in 2013, in 2,615 patients from 13 studies, in stage II–IV CRC, which found a significant association with worse OS (HR 1.90, 95% CI 1.58–2.29, eight studies) and DFS (HR 2.16, 95% CI 1.53–3.05, three studies) (50).

CXCL12 is a chemokine that is not CAF specific, but when present, it contributes to the formation of a tumor-supportive microenvironment through angiogenesis, immunomodulation, and extracellular matrix remodeling (12). It was associated with a threefold increased risk of recurrence or death, but overall, the HR was non-significant in this analysis. In a previous meta-analysis (six studies, 109 patients) addressing the prognostic value of CXCL12 expression in several subtypes of cancer including CRC, there was no statistically significant association with either RFS (HR 0.83, 95% CI 0.46–1.49, *p* = 0.48, seven cohorts, *n* = 1,446) or OS (HR 1.27, 95% CI 0.64–2.51, *p* = 0.49) (51).

POSTN is a matrix glycoprotein with a role in EMT, invasion, and metastasis (62) and is a marker of ECM CAFs (15). POSTN was significantly associated with poorer outcomes, showing a 2.29-fold increased risk for relapse-free survival (HR = 2.29) and a 2.66-fold increased risk for overall survival (HR = 2.66), with both associations being statistically significant.

In a review that discussed the prognosis and therapeutic application of CAF expression, only COL11A1 was reported to be a potential negative prognostic indicator in right-sided colon tumors (10). COL11A1 is a fibrillar collagen protein, primarily involved in cartilage formation, which is thought to be a novel biomarker with specificity to CAFs and cancer progression, through ECM remodeling (7). However, our search strategy failed to find any articles on COL11A1 protein expression in relation to prognosis that met our inclusion criteria, with studies focusing only on gene signature assays (63).

#### 4.2.1 Individualized biomarkers

In this analysis, MMP-9 was consistently associated with poor prognosis. Across five studies (*N* = 1,139), the hazard ratio for disease-free survival was 1.8 (*p* < 0.001). Intratumoral cytoplasmic expression was assessed qualitatively based on staining [in the studies of Salem (64), Chu (31), and Buhmeida (65)] and quantitatively [in two studies by Chu (31) and Ogata (66)] based on the percentage of stained cells. In contrast, one study (*N* = 340) showed a near-protective effect, likely due to its focus on MMP-9 expression in lymphocytes at the invasive front, assessed quantitatively (34).

MMP-9 is a member of the matrix metalloproteinase family that plays an important role in degrading compounds of the ECM, such as collagen fibers, fibrin, and decorin (67). In CRC, it is involved in degrading collagen type IV fibers in the basal membrane, favoring tumor progression (65). A meta-analysis by Wang et al. (including 18 studies, *N* = 3,944) reported that MMP-9 expression was not significantly associated with a decrease in OS (risk ratio 1.48, 95% CI 0.97 to 2.24, *p* = 0.069) or DFS (risk ratio 1.60, 95% CI 0.87 to 2.94, *p* = 0.133) (68).

CD163 is a macrophage-specific protein hemoglobin specific for M2-tumor-associated macrophages (69), found at increased levels in stages III–IV, compared to stages I–II of CRC, and considered to be involved in immune suppression (70). In the present meta-analysis, reports from four publications found an association between CD163-positive tumors and outcomes, with an almost twofold higher risk of relapse or progression, albeit not with OS. Conversely, Ye et al. reported consistently worse survival in all three cohorts of their study [training (*N* = 359), test (*N* = 249), and validation (*N* = 400)] (30). Our findings are consistent with those of other reviews, such as that by Larionova et al., where the presence of M2 tumor-associated macrophages was found to be associated with aggressive disease, tumor progression, angiogenesis, and lymphatic metastasis in many types of cancer, including CRC (71).

A meta-analysis investigating the prognostic role of TAM in all stages of CRC, in the subgroup of CD163-positive tumors, including six articles (*N* = 1,550), reported a pooled HR showing a 40% increase in the risk of death at 5 years in CRC, but without reaching statistical significance (HR 1.41, 95% CI 0.83–2.39) (72).

Vimentin represents one of the most widely expressed members of the type III intermediate filament protein family (11), not specific for CAF, and recognized as a marker of EMT in CRC, possibly revealing an epithelial origin of CAFs (73).

Another specific meta-analysis (11 studies, *N* = 1,969) including all stages of CRC showed an association between VIM and survival, with a 60% increase in the risk of death (HR for OS 1.633, 95% CI 1.223–2.181, *p* = 0.001) and worse DFS (HR 2.802, 1.421–5.527, *p* = 0.035) (74).

In this analysis, VIM was significantly associated with poor overall survival in stage I–IV CRC (four studies, *N* = 507) (36, 75, 76) including localized stages with an HR of 2.01 (*p* = 0.015) reported by Birzozowa-Zasada et al. (*N* = 97) (35), whereas no significant association was found for disease-free survival (three studies, *N* = 513) (36, 75, 77). One explanation could be the difference in populations enrolled in these studies. Articles included in the DFS analysis had more homogeneous populations, with only localized disease, and all used a score for quantification of positivity in the stroma. Conversely, for OS, the populations were more heterogeneous, including also stage IV patients and Caucasian patients (*N* = 97), and in 105 cases, Xiao et al. assessed cytoplasmic expression in tumor cells (76).

Transgelin 2 is an actin-binding protein, expressed in fibroblasts, endothelial cells, and smooth muscle cells, and has a key role in cytoskeleton rearrangement and cell mobility (78). It is a marker of activated myofibroblasts and CAF B (16). Its role in CRC is still controversial, with earlier studies suggesting that it acts as a tumor suppressor (78). At the same time, later research correlated the overexpression of TAGLN2 in advanced stages with tumor progression via the TGFβ signaling pathway (79).

In our review, in the qualitative analysis, we found three studies that met our inclusion criteria, two of which used positive/high expression as a reference: Zhao (*N* = 126, stages I–IV) (27), who used quantitative assessment of the biomarker in tumor cells, and Xu (stage III disease) (28), who used a score for determination, and most of the staining was not in tumor cells but in cells of the lamina propria. Both these studies showed a protective effect of TAGLN2 on OS and DFS. On the other hand, we retained only one study in the meta-analysis, showing a borderline negative impact on OS (HR = 1.6) in stage I–IV patients (*N* = 120), with assessment of TAGLN2 in the intratumoral cytoplasm using a score combining intensity and area of stained cells (80).

A review by Dvorakova et al. considered that a possible explanation for the contradictory results in the literature regarding the association TAGLN2 with prognosis stems from the fact that it has a dual effect. Indeed, at the first stages of carcinogenesis, its level decreases in tumor cells, while in advanced stages, it may increase in stromal cells as cancer progresses (81).

TNC is an ECM protein with a structural component of epidermal growth factor-like and fibronectin type III domains that can combine with other ECM molecules, like integrins and fibronectin (7). It is non-specific to CAFs, but when present in CAFs in CRC, it promotes EMT and proliferation involved in tumor growth and metastasis via hedgehog signaling (31).

We identified four studies evaluating TNC in a total of 625 patients—one limited to stage II–III and three including stage I–IV CRC. A significant association with worse DFS and OS was observed in only one study (*N* = 259, stage II–III), which used a combined score based on both staining intensity and the proportion of stained cells. However, this study did not report a conclusive result for DFS, and in a second cohort of localized-stage patients, the association with OS was no longer significant.

Our results are in concordance with the data found in the literature, where TNC has been described to be associated with poor OS in a meta-analysis studying TNC expression in multiple types of cancers [*N* = 2,732, 18 studies, pooled HR of 1.73 (1.29–2.32), *p* < 0.001] (82).

PDGFR, a platelet-derived growth factor tyrosine kinase receptor, divided into two classes (α and β), is a common marker used for fibroblast identification, but is not specific. CAFs expressing the PDGF/PDGFR axis, through paracrine signaling and vascular injuries, favor angiogenesis and metastasis (7). In our meta-analysis, we retained only one study reporting the prognostic value of PDGFR-β, with a twofold higher risk of recurrence in 21 patients with stage I–IV disease but no significant impact on OS. Conversely, the other study of PDGFR, by Mezheyeuski (*N* = 311, stage IV), included only in the qualitative analysis, showed that low expression of PDGFR-β is associated with shorter OS, with an HR of 1.82 (95% CI 1.009–3.04), but the assessment was done in perivascular cells (83).

Podoplanin is a sialo glycoprotein, whose expression in CAFs is associated with an origin from vascular endothelial cells and carries good prognostic value for DFS only, indicating a strong protective effect. PDPN-high CAFs in CRC seem to be protective against cell invasion and are associated with favorable clinicopathological parameters and prolonged DFS (17). However, there have been data linking this biomarker with poor prognosis, advanced stage, and EMT in lung, breast, or bladder cancer, through lymphangiogenesis, and therefore, the promotion of metastases (18).

In the present meta-analysis, we included only one study (*N* = 120), in stage II–III CRC, which showed a good prognostic value both for DFS and OS (47). In another study, Cai et al. (*N* = 164, stages I–IV) reported a non-significant HR of 0.646 (95% CI 0.37–1.12) (48). Both these studies assessed the expression by proportion of stained cells, using a monoclonal antibody, but with different cutoffs: 30% for Yamanashi (47) and 10% for Cai (48).

### 4.3 Mechanistic insights

Biomarkers from CAFs are generally of negative prognostic value, through secretion of growth factors, angiogenesis, inhibition of immune cells, and remodeling of ECM (8). As for the few studies that found a positive association with prognosis, PDPN in CRC is associated with a decrease in lymph node invasion, possibly through interaction with CD9, known to be a metastasis suppressor (18), and TAGLN, possibly through actin binding and cytoskeleton stabilization. However, these results must be interpreted with caution, due to their small, retrospective, specific cohorts (27).

### 4.4 Heterogeneity and context-specific effects

The high *I*² values for DFS and OS suggest substantial heterogeneity among the studies included in the meta-analysis, explaining variability in the results. In our study, there was high variation in the study populations, ranging from very small, specific cohorts to studies with over 1,000 patients. Also, 30 studies included only localized disease, while 7 studies included only metastatic stage disease. Regarding the chemotherapy regimens, the most frequent option was FOLFOX/CAPEOX, followed by fluoropyrimidine monotherapy and also targeted therapy in case of metastatic disease. In 36 studies, however, there is no information regarding the oncological treatment, and 7 studies reported cases without adjuvant treatment.

Regarding differences in methodology, apart from 3 studies that used ELISA from tissue specimens for biomarker determination, all the other studies detected CAF biomarkers through IHC, and 15 articles also reported performing immunoblotting for confirmation or to assess therapeutic effects on cell lines. For the quantification of positivity, 16/59 studies used qualitative analysis (by staining intensity), 28/59 used the area of stained cells, and 14 used a score (combination of intensity and percentage of positive cells).

There was no uniformity across studies regarding cutoffs, with some using the median value and others calculating it using receiver operating characteristic curves (ROCs) or simply using a predetermined value. Another source of heterogeneity comes from different assessment strategies, i.e., whether the biomarker was tested specifically at the invasion front (6 articles), in the surrounding stroma (12 articles), or in the tumor in general. Eighteen articles analyzed the outcomes separately for different assessment strategies, while others did not define the strategy clearly. Regarding tissue specimens, only 4 studies used liver metastasis for marker determination, while the rest used the primary tumor. Given these manifold sources of variability, standardization is needed for future research in this field.

Furthermore, these proteins are not only markers of CAF presence, but PDPN, for example, is also used as a marker for lymphovascular density quantification in CRC (84), which might be a source of confusion in the literature search.

### 4.5 Clinical implications

In current practice, CAF biomarkers can help better characterize the prognosis of both localized and liver metastatic CRC and may also guide treatment decisions—particularly systemic therapies—given their apparent correlation with responses to various cytotoxic and targeted agents. To date, however, there has been limited translation of these findings into clinical practice due to variability and lack of universal validation.

### 4.6 Study limitations

Our study has some limitations, including potential publication bias, which affects the selection of studies. Most of the articles were retrospective studies (68/84), 41 in small, specific cohorts. Six of them included patients who had been enrolled in clinical trials. There was also inconsistent reporting of hazard ratios and confidence intervals across studies: 15 articles reported more than one HR, and there was a lack of data on some of the less well-explored CAF biomarkers, such as PDPN, with 3 articles found, of which only 2 met the criteria for inclusion in the final statistical analysis. Other markers with only 1 study each included in the meta-analysis were αSMA, TAGLN2, and PDGFR-β, with 2, 3, and 2 articles, respectively, included in the qualitative assessment. Finally, other potential limitations include the fact that we ran our literature search in only two databases (Medline and Embase), and we also limited our selection to publications in English. Additional potential sources of bias arise from the subjective application of the Newcastle–Ottawa Scale and QUADAS-2 tools, even though the consistency between reviewers was strong. An additional limitation is the pooling of DFS, RFS, and PFS, which, despite reflecting different clinical endpoints, was necessary due to the inconsistent and overlapping definitions across studies.

### 4.7 Future directions

This review and meta-analysis opens the way to the validation of promising prognostic biomarkers, such as MMP-9 and CD163, in larger studies, as well as the exploration of the role of CAF subtypes and their interplay with other elements of the TME.

Our future research directions will focus on characterizing CAFs in liver metastasis of CRC, and how they can refine the features of histopathological growth patterns of tissue reaction at the tumor–host tissue interference (85). By comparing liver metastasis CAFS to those from the primary tumor, we hope to better understand the mechanisms of dormancy and early or late relapse in this type of cancer.

### 4.8 Conclusion

This meta-analysis is one of the most exhaustive and complete to date, and it reinforces the importance of CAF biomarkers in predicting CRC outcomes, most of which carry a negative prognosis. While not yet ready for clinical application, with standard assay development being necessary, our findings can serve as the basis for validation studies in larger cohorts of patients and especially in metastatic stages. The role of CAF biomarkers should probably be assessed in relation to other microenvironmental elements, such as lymphocytic infiltrates.

Few studies have assessed the expression of markers of good prognosis by IHC, probably considered less important for clinical practice. We should consider the inducibility of certain CAFs with a good prognosis.

## Data Availability

The original contributions presented in the study are included in the article/**Supplementary Material**. Further inquiries can be directed to the corresponding authors.
