# Supplementary figures and images for: Cancer-associated fibroblasts and their prognostic role in colorectal cancer: review and meta-analysis
Source: Front Oncol. 2025 Dec 10;15:1635055. doi: 10.3389/fonc.2025.1635055 (PMC12727558; doi:10.3389/fonc.2025.1635055)

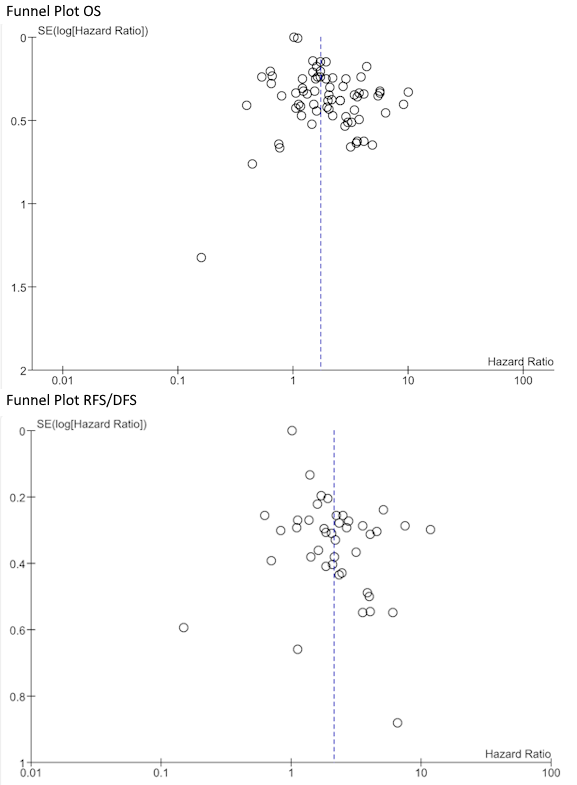

Supplement: Supplementary file 1 [file Image1.png]

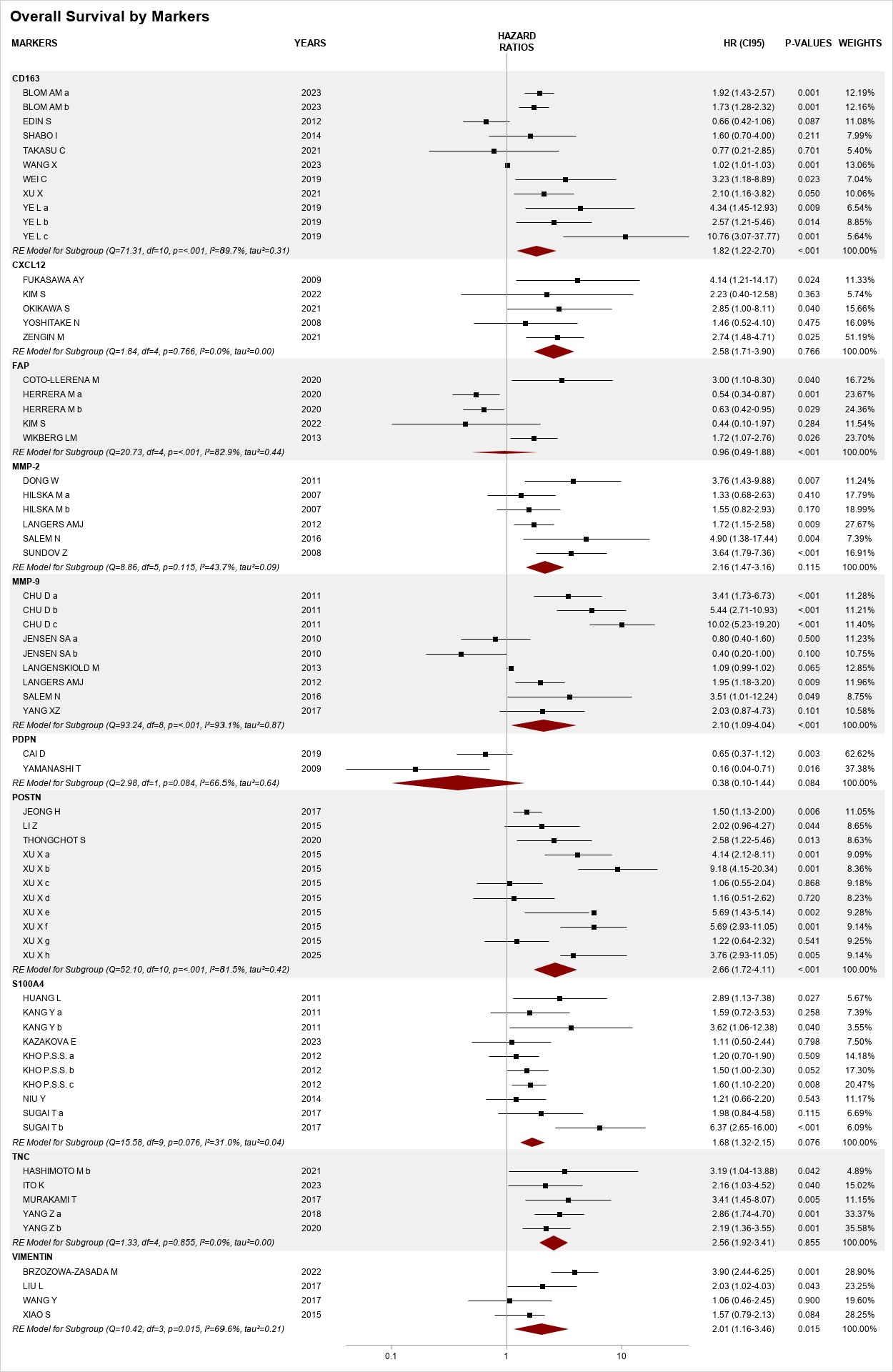

Supplement: Supplementary file 2 [file Image2.jpg]
