# Supplementary material for: Cancer-associated fibroblasts and their prognostic role in colorectal cancer: review and meta-analysis
Source: Front Oncol. 2025 Dec 10;15:1635055. doi: 10.3389/fonc.2025.1635055 (PMC12727558; doi:10.3389/fonc.2025.1635055)
Supplement: Supplementary file 3 [file Table1.docx]

Tabel 1. Search and retrieval strategy Medline

| TNC – PUBMED 1 |
| --- |
| ("colorectal neoplasms/blood"[MeSH Terms] OR "colorectal neoplasms/chemically induced"[MeSH Terms] OR "colorectal neoplasms/chemistry"[MeSH Terms] OR "colorectal neoplasms/classification"[MeSH Terms] OR "colorectal neoplasms/complications"[MeSH Terms] OR "colorectal neoplasms/diagnosis"[MeSH Terms] OR "colorectal neoplasms/drug therapy"[MeSH Terms] OR "colorectal neoplasms/epidemiology"[MeSH Terms] OR "colorectal neoplasms/ethnology"[MeSH Terms] OR "colorectal neoplasms/etiology"[MeSH Terms] OR "colorectal neoplasms/genetics"[MeSH Terms] OR "colorectal neoplasms/immunology"[MeSH Terms] OR "colorectal neoplasms/metabolism"[MeSH Terms] OR "colorectal neoplasms/mortality"[MeSH Terms] OR "colorectal neoplasms/pathology"[MeSH Terms] OR "colorectal neoplasms/physiopathology"[MeSH Terms] OR "colorectal neoplasms/radiotherapy"[MeSH Terms] OR "colorectal neoplasms/secondary"[MeSH Terms] OR "colorectal neoplasms/surgery"[MeSH Terms] OR "colorectal neoplasms/therapy"[MeSH Terms] OR ("colorectal neoplasm*"[All Fields] OR "colorectal tumour*"[All Fields] OR "colorectal cancer*"[All Fields] OR "colorectal adenocarcinoma"[All Fields] OR "colorectal carcinoma"[All Fields] OR "colon neoplasm*"[All Fields] OR "colon cancer*"[All Fields] OR "colon tumour*"[All Fields] OR "colon tumor*"[All Fields] OR "colon adenocarcinoma"[All Fields] OR "colon carcinoma"[All Fields] OR "rectal cancer*"[All Fields] OR "rectal neoplasm*"[All Fields] OR "rectal tumor*"[All Fields] OR "rectal tumour*"[All Fields] OR "rectal carcinoma"[All Fields] OR "rectal adenocarcinoma"[All Fields])) AND ("Prognosis"[MeSH Terms] OR "Survival"[MeSH Terms] OR ("Prognosis"[All Fields] OR "prognostic"[All Fields] OR "Survival"[All Fields] OR "outcome"[All Fields])) AND ("tnc protein human"[Supplementary Concept] OR ("tenascin/adverse effects"[MeSH Terms] OR "tenascin/analysis"[MeSH Terms] OR "tenascin/blood"[MeSH Terms] OR "tenascin/chemistry"[MeSH Terms] OR "tenascin/classification"[MeSH Terms] OR "tenascin/drug effects"[MeSH Terms] OR "tenascin/genetics"[MeSH Terms] OR "tenascin/immunology"[MeSH Terms] OR "tenascin/metabolism"[MeSH Terms] OR "tenascin/pharmacology"[MeSH Terms] OR "tenascin/physiology"[MeSH Terms] OR "tenascin/radiation effects"[MeSH Terms] OR "tenascin/therapeutic use"[MeSH Terms] OR "tenascin/toxicity"[MeSH Terms]) OR ("TNC"[All Fields] OR "Cytotactin"[All Fields] OR "Hexabrachion"[All Fields] OR "tenascin c"[All Fields] OR "tenascin c"[All Fields] OR "J1-200-220"[All Fields] OR "TN-C"[All Fields])) |

| TNC - PUBMED 2 |
| --- |
| (("Tenascin"[Mesh] OR "Tenascin C" OR "TNC") |
| AND |
| ("Colorectal Neoplasms"[Mesh] OR "Colorectal Cancer" OR "Colon Cancer" OR "Rectal Cancer" OR "Colorectal Carcinoma" OR "Colorectal Adenocarcinoma" OR "Colonic Neoplasms" OR "Rectal Neoplasms") |
| AND |
| ("Prognosis"[Mesh] OR "Prognostic Value" OR "Prognostic Factor" OR "Survival" OR "Overall Survival" OR "Disease-Free Survival" OR "Relapse-Free Survival" OR "Progression-Free Survival" OR "Event-Free Survival" OR "Hazard Ratio" OR "Kaplan Meier" OR "Recurrence" OR "Time to progression")) |

| MMP9, MMP2 – Pubmed 1 |
| --- |
| ("colorectal neoplasms/blood"[MeSH Terms] OR "colorectal neoplasms/chemically induced"[MeSH Terms] OR "colorectal neoplasms/chemistry"[MeSH Terms] OR "colorectal neoplasms/classification"[MeSH Terms] OR "colorectal neoplasms/complications"[MeSH Terms] OR "colorectal neoplasms/diagnosis"[MeSH Terms] OR "colorectal neoplasms/drug therapy"[MeSH Terms] OR "colorectal neoplasms/epidemiology"[MeSH Terms] OR "colorectal neoplasms/ethnology"[MeSH Terms] OR "colorectal neoplasms/etiology"[MeSH Terms] OR "colorectal neoplasms/genetics"[MeSH Terms] OR "colorectal neoplasms/immunology"[MeSH Terms] OR "colorectal neoplasms/metabolism"[MeSH Terms] OR "colorectal neoplasms/mortality"[MeSH Terms] OR "colorectal neoplasms/pathology"[MeSH Terms] OR "colorectal neoplasms/physiopathology"[MeSH Terms] OR "colorectal neoplasms/radiotherapy"[MeSH Terms] OR "colorectal neoplasms/secondary"[MeSH Terms] OR "colorectal neoplasms/surgery"[MeSH Terms] OR "colorectal neoplasms/therapy"[MeSH Terms] OR ("colorectal neoplasm*"[All Fields] OR "colorectal tumour*"[All Fields] OR "colorectal cancer*"[All Fields] OR "colorectal adenocarcinoma"[All Fields] OR "colorectal carcinoma"[All Fields] OR "colon neoplasm*"[All Fields] OR "colon cancer*"[All Fields] OR "colon tumour*"[All Fields] OR "colon tumor*"[All Fields] OR "colon adenocarcinoma"[All Fields] OR "colon carcinoma"[All Fields] OR "rectal cancer*"[All Fields] OR "rectal neoplasm*"[All Fields] OR "rectal tumor*"[All Fields] OR "rectal tumour*"[All Fields] OR "rectal carcinoma"[All Fields] OR "rectal adenocarcinoma"[All Fields])) AND ("Prognosis"[MeSH Terms] OR "Survival"[MeSH Terms] OR ("Prognosis"[All Fields] OR "prognostic"[All Fields] OR "Survival"[All Fields] OR "outcome"[All Fields])) AND ("matrix metalloproteinase 9/adverse effects"[MeSH Terms] OR "matrix metalloproteinase 9/blood"[MeSH Terms] OR "matrix metalloproteinase 9/chemistry"[MeSH Terms] OR "matrix metalloproteinase 9/classification"[MeSH Terms] OR "matrix metalloproteinase 9/drug effects"[MeSH Terms] OR "matrix metalloproteinase 9/genetics"[MeSH Terms] OR "matrix metalloproteinase 9/immunology"[MeSH Terms] OR "matrix metalloproteinase 9/metabolism"[MeSH Terms] OR "matrix metalloproteinase 9/pharmacology"[MeSH Terms] OR "matrix metalloproteinase 9/physiology"[MeSH Terms] OR "matrix metalloproteinase 9/radiation effects"[MeSH Terms] OR "matrix metalloproteinase 9/therapeutic use"[MeSH Terms] OR "matrix metalloproteinase 9/toxicity"[MeSH Terms] OR "mmp9 protein human"[Supplementary Concept] OR ("MMP9"[All Fields] OR "metalloproteinase 9 matrix"[All Fields] OR "92 kda type iv collagenase"[All Fields] OR "92 kda type iv collagenase"[All Fields] OR "matrix metalloproteinase 9"[All Fields] OR "matrix metalloproteinase 9"[All Fields] OR "92 kda gelatinase"[All Fields] OR "92 kda gelatinase"[All Fields] OR "MMP-9 Metalloproteinase"[All Fields] OR "metalloproteinase mmp 9"[All Fields] OR "MMP9 Metalloproteinase"[All Fields] OR "metalloproteinase mmp9"[All Fields] OR "Gelatinase B"[All Fields])) |

| MMP9-PUBMED 2 |
| --- |
| (("Matrix Metalloproteinase 9"[Mesh] OR "matrix metalloproteinase 9" OR "MMP-9") |
| AND |
| ("Colorectal Neoplasms"[Mesh] OR "Colorectal Cancer" OR "Colon Cancer" OR "Rectal Cancer" OR "Colorectal Carcinoma" OR "Colorectal Adenocarcinoma" OR "Colonic Neoplasms" OR "Rectal Neoplasms") |
| AND |
| ("Prognosis"[Mesh] OR "Prognostic Value" OR "Prognostic Factor" OR "Survival" OR "Overall Survival" OR "Disease-Free Survival" OR "Relapse-Free Survival" OR "Progression-Free Survival" OR "Event-Free Survival" OR "Hazard Ratio" OR "Kaplan Meier" OR "Recurrence" OR "Time to progression")) |

| MMP2 PUBMED 2 |
| --- |
| (("Matrix Metalloproteinase 2"[Mesh]) OR ("matrix metalloproteinase 2") OR ("MMP-2") |
| AND |
| ("Colorectal Neoplasms"[Mesh] OR "Colorectal Cancer" OR "Colon Cancer" OR "Rectal Cancer" OR "Colorectal Carcinoma" OR "Colorectal Adenocarcinoma" OR "Colonic Neoplasms" OR "Rectal Neoplasms") |
| AND |
| ("Prognosis"[Mesh] OR "Prognostic Value" OR "Prognostic Factor" OR "Survival" OR "Overall Survival" OR "Disease-Free Survival" OR "Relapse-Free Survival" OR "Progression-Free Survival" OR "Event-Free Survival" OR "Hazard Ratio" OR "Kaplan Meier" OR "Recurrence" OR "Time to progression")) |

| CXCL12 – Pubmed |
| --- |
| **((( "Chemokine CXCL12/agonists"[Mesh] OR "Chemokine CXCL12/analysis"[Mesh] OR "Chemokine CXCL12/antagonists and inhibitors"[Mesh] OR "Chemokine CXCL12/biosynthesis"[Mesh] OR "Chemokine CXCL12/blood"[Mesh] OR "Chemokine CXCL12/chemistry"[Mesh] OR "Chemokine CXCL12/classification"[Mesh] OR "Chemokine CXCL12/drug effects"[Mesh] OR "Chemokine CXCL12/genetics"[Mesh] OR "Chemokine CXCL12/immunology"[Mesh] OR "Chemokine CXCL12/isolation and purification"[Mesh] OR "Chemokine CXCL12/metabolism"[Mesh] OR "Chemokine CXCL12/pharmacokinetics"[Mesh] OR "Chemokine CXCL12/pharmacology"[Mesh] OR "Chemokine CXCL12/physiology"[Mesh] OR "Chemokine CXCL12/therapeutic use"[Mesh] ) OR "Chemokine (C-X-C Motif) Ligand 12" OR "CXCL12" OR" Stromal Cell Derived Factor 1" OR "SDF1*") AND (( "Colorectal Neoplasms/blood"[Mesh] OR "Colorectal Neoplasms/chemically induced"[Mesh] OR "Colorectal Neoplasms/chemistry"[Mesh] OR "Colorectal Neoplasms/complications"[Mesh] OR "Colorectal Neoplasms/diagnosis"[Mesh] OR "Colorectal Neoplasms/drug therapy"[Mesh] OR "Colorectal Neoplasms/epidemiology"[Mesh] OR "Colorectal Neoplasms/ethnology"[Mesh] OR "Colorectal Neoplasms/etiology"[Mesh] OR "Colorectal Neoplasms/genetics"[Mesh] OR "Colorectal Neoplasms/immunology"[Mesh] OR "Colorectal Neoplasms/metabolism"[Mesh] OR "Colorectal Neoplasms/mortality"[Mesh] OR "Colorectal Neoplasms/pathology"[Mesh] OR "Colorectal Neoplasms/physiopathology"[Mesh] OR "Colorectal Neoplasms/radiotherapy"[Mesh] OR "Colorectal Neoplasms/secondary"[Mesh] OR "Colorectal Neoplasms/surgery"[Mesh] OR "Colorectal Neoplasms/therapy"[Mesh] ) OR "Colorectal Neoplasm*" OR "Colorectal tumour*" OR "Colorectal tumour*" OR "Colorectal cancer*" OR " colorectal adenocarcinoma" OR "colorectal carcinoma" OR "colon neoplasm*" OR "colon cancer*" OR "colon tumour*" OR "colon tumor*" OR " colon adenocarcinoma" OR "colon carcinoma" OR "rectal cancer*" OR "Rectal neoplasm*" OR "rectal tumor*" OR "rectal tumour" OR "rectal carcinoma" OR "rectal adenocarcinoma")) AND ("Prognosis"[Mesh] OR "prognosis" OR "prognostic" OR "survival")** Filters: **English, Humans, from 2003 - 2024** |

| FAP |
| --- |
| Pubmed |
| ("colorectal neoplasms/blood"[MeSH Terms] OR "colorectal neoplasms/chemically induced"[MeSH Terms] OR "colorectal neoplasms/chemistry"[MeSH Terms] OR "colorectal neoplasms/classification"[MeSH Terms] OR "colorectal neoplasms/complications"[MeSH Terms] OR "colorectal neoplasms/diagnosis"[MeSH Terms] OR "colorectal neoplasms/drug therapy"[MeSH Terms] OR "colorectal neoplasms/epidemiology"[MeSH Terms] OR "colorectal neoplasms/ethnology"[MeSH Terms] OR "colorectal neoplasms/etiology"[MeSH Terms] OR "colorectal neoplasms/genetics"[MeSH Terms] OR "colorectal neoplasms/immunology"[MeSH Terms] OR "colorectal neoplasms/metabolism"[MeSH Terms] OR "colorectal neoplasms/mortality"[MeSH Terms] OR "colorectal neoplasms/pathology"[MeSH Terms] OR "colorectal neoplasms/physiopathology"[MeSH Terms] OR "colorectal neoplasms/radiotherapy"[MeSH Terms] OR "colorectal neoplasms/secondary"[MeSH Terms] OR "colorectal neoplasms/surgery"[MeSH Terms] OR "colorectal neoplasms/therapy"[MeSH Terms] OR ("colorectal neoplasm*"[All Fields] OR "colorectal tumour*"[All Fields] OR "colorectal cancer*"[All Fields] OR "colorectal adenocarcinoma"[All Fields] OR "colorectal carcinoma"[All Fields] OR "colon neoplasm*"[All Fields] OR "colon cancer*"[All Fields] OR "colon tumour*"[All Fields] OR "colon tumor*"[All Fields] OR "colon adenocarcinoma"[All Fields] OR "colon carcinoma"[All Fields] OR "rectal cancer*"[All Fields] OR "rectal neoplasm*"[All Fields] OR "rectal tumor*"[All Fields] OR "rectal tumour*"[All Fields] OR "rectal carcinoma"[All Fields] OR "rectal adenocarcinoma"[All Fields])) AND ("Prognosis"[MeSH Terms] OR "Survival"[MeSH Terms] OR ("Prognosis"[All Fields] OR "prognostic"[All Fields] OR "Survival"[All Fields] OR "outcome"[All Fields])) AND ("fibroblast activation protein alpha"[Supplementary Concept] OR ("FAPalpha"[All Fields] OR "fibroblast activation protein*"[All Fields] OR "seprase"[All Fields] OR "FAP protein"[All Fields] OR "fibroblast |
| activating factor"[All Fields] OR "fibroblast proliferation factor"[All Fields])) |

| FAP – PUBMED 2 |
| --- |
| (("fibroblast activation protein alpha"[Supplementary Concept] OR "Fibroblast activation protein" OR "FAP") |
| AND |
| ("Colorectal Neoplasms"[Mesh] OR "Colorectal Cancer" OR "Colon Cancer" OR "Rectal Cancer" OR "Colorectal Carcinoma" OR "Colorectal Adenocarcinoma" OR "Colonic Neoplasms" OR "Rectal Neoplasms") |
| AND |
| ("Prognosis"[Mesh] OR "Prognostic Value" OR "Prognostic Factor" OR "Survival" OR "Overall Survival" OR "Disease-Free Survival" OR "Relapse-Free Survival" OR "Progression-Free Survival" OR "Event-Free Survival" OR "Hazard Ratio" OR "Kaplan Meier" OR "Recurrence" OR "Time to progression")) |

| PDPN – PUBMED |
| --- |
| ("colorectal neoplasms/blood"[MeSH Terms] OR "colorectal neoplasms/chemically induced"[MeSH Terms] OR "colorectal neoplasms/chemistry"[MeSH Terms] OR "colorectal neoplasms/classification"[MeSH Terms] OR "colorectal neoplasms/complications"[MeSH Terms] OR "colorectal neoplasms/diagnosis"[MeSH Terms] OR "colorectal neoplasms/drug therapy"[MeSH Terms] OR "colorectal neoplasms/epidemiology"[MeSH Terms] OR "colorectal neoplasms/ethnology"[MeSH Terms] OR "colorectal neoplasms/etiology"[MeSH Terms] OR "colorectal neoplasms/genetics"[MeSH Terms] OR "colorectal neoplasms/immunology"[MeSH Terms] OR "colorectal neoplasms/metabolism"[MeSH Terms] OR "colorectal neoplasms/mortality"[MeSH Terms] OR "colorectal neoplasms/pathology"[MeSH Terms] OR "colorectal neoplasms/physiopathology"[MeSH Terms] OR "colorectal neoplasms/radiotherapy"[MeSH Terms] OR "colorectal neoplasms/secondary"[MeSH Terms] OR "colorectal neoplasms/surgery"[MeSH Terms] OR "colorectal neoplasms/therapy"[MeSH Terms] OR ("colorectal neoplasm*"[All Fields] OR "colorectal tumour*"[All Fields] OR "colorectal cancer*"[All Fields] OR "colorectal adenocarcinoma"[All Fields] OR "colorectal carcinoma"[All Fields] OR "colon neoplasm*"[All Fields] OR "colon cancer*"[All Fields] OR "colon tumour*"[All Fields] OR "colon tumor*"[All Fields] OR "colon adenocarcinoma"[All Fields] OR "colon carcinoma"[All Fields] OR "rectal cancer*"[All Fields] OR "rectal neoplasm*"[All Fields] OR "rectal tumor*"[All Fields] OR "rectal tumour*"[All Fields] OR "rectal carcinoma"[All Fields] OR "rectal adenocarcinoma"[All Fields])) AND ("Prognosis"[MeSH Terms] OR "Survival"[MeSH Terms] OR ("Prognosis"[All Fields] OR "prognostic"[All Fields] OR "Survival"[All Fields] OR "outcome"[All Fields])) AND ("pdpn protein human"[Supplementary Concept] OR ("PDPN"[All Fields] OR "T1alpha protein"[All Fields] OR "T1 alpha protein"[All Fields] OR "podoplanin"[All Fields] OR "gp38 protein"[All Fields] OR "aggrus protein"[All Fields])) |

| PDPN – PUBMED 2 |
| --- |
| (("Podoplanin"[Mesh] OR "Podoplanin" OR "PDPN") |
| AND |
| ("Colorectal Neoplasms"[Mesh] OR "Colorectal Cancer" OR "Colon Cancer" OR "Rectal Cancer" OR "Colorectal Carcinoma" OR "Colorectal Adenocarcinoma" OR "Colonic Neoplasms" OR "Rectal Neoplasms") |
| AND |
| ("Prognosis"[Mesh] OR "Prognostic Value" OR "Prognostic Factor" OR "Survival" OR "Overall Survival" OR "Disease-Free Survival" OR "Relapse-Free Survival" OR "Progression-Free Survival" OR "Event-Free Survival" OR "Hazard Ratio" OR "Kaplan Meier" OR "Recurrence" OR "Time to progression")) |

| **CD163 - PUBMED** |
| --- |
| ("colorectal neoplasms/blood"[MeSH Terms] OR "colorectal neoplasms/chemically induced"[MeSH Terms] OR "colorectal neoplasms/chemistry"[MeSH Terms] OR "colorectal neoplasms/classification"[MeSH Terms] OR "colorectal neoplasms/complications"[MeSH Terms] OR "colorectal neoplasms/diagnosis"[MeSH Terms] OR "colorectal neoplasms/drug therapy"[MeSH Terms] OR "colorectal neoplasms/epidemiology"[MeSH Terms] OR "colorectal neoplasms/ethnology"[MeSH Terms] OR "colorectal neoplasms/etiology"[MeSH Terms] OR "colorectal neoplasms/genetics"[MeSH Terms] OR "colorectal neoplasms/immunology"[MeSH Terms] OR "colorectal neoplasms/metabolism"[MeSH Terms] OR "colorectal neoplasms/mortality"[MeSH Terms] OR "colorectal neoplasms/pathology"[MeSH Terms] OR "colorectal neoplasms/physiopathology"[MeSH Terms] OR "colorectal neoplasms/radiotherapy"[MeSH Terms] OR "colorectal neoplasms/secondary"[MeSH Terms] OR "colorectal neoplasms/surgery"[MeSH Terms] OR "colorectal neoplasms/therapy"[MeSH Terms] OR ("colorectal neoplasm*"[All Fields] OR "colorectal tumour*"[All Fields] OR "colorectal cancer*"[All Fields] OR "colorectal adenocarcinoma"[All Fields] OR "colorectal carcinoma"[All Fields] OR "colon neoplasm*"[All Fields] OR "colon cancer*"[All Fields] OR "colon tumour*"[All Fields] OR "colon tumor*"[All Fields] OR "colon adenocarcinoma"[All Fields] OR "colon carcinoma"[All Fields] OR "rectal cancer*"[All Fields] OR "rectal neoplasm*"[All Fields] OR "rectal tumor*"[All Fields] OR "rectal tumour*"[All Fields] OR "rectal carcinoma"[All Fields] OR "rectal adenocarcinoma"[All Fields])) AND ("Prognosis"[MeSH Terms] OR "Survival"[MeSH Terms] OR ("Prognosis"[All Fields] OR "prognostic"[All Fields] OR "Survival"[All Fields] OR "outcome"[All Fields])) AND ("CD163 antigen"[Supplementary Concept] OR ("CD163"[All Fields] OR (("collect dyn"[Journal] OR "can dimens"[Journal] OR "cd"[All Fields]) AND "163"[All Fields] AND ("protein s"[All Fields] OR "proteinous"[All Fields] OR "proteins"[MeSH Terms] OR "proteins"[All Fields] OR "protein"[All Fields])) OR ("CD163 antigen"[Supplementary Concept] OR "CD163 antigen"[All Fields]) OR "rm3 1 antigen"[All Fields] OR "rm3 1 antigen"[All Fields] OR "CD163B"[All Fields] OR ("CD163 antigen"[Supplementary Concept] OR "CD163 antigen"[All Fields]) OR "CD163 antigen"[All Fields])) |
|  |

| CD163 – PUBMED 2 |
| --- |
| (("CD163 Antigen"[Mesh] OR "CD163") |
| AND |
| ("Colorectal Neoplasms"[Mesh] OR "Colorectal Cancer" OR "Colon Cancer" OR "Rectal Cancer" OR "Colorectal Carcinoma" OR "Colorectal Adenocarcinoma" OR "Colonic Neoplasms" OR "Rectal Neoplasms") |
| AND |
| ("Prognosis"[Mesh] OR "Prognostic Value" OR "Prognostic Factor" OR "Survival" OR "Overall Survival" OR "Disease-Free Survival" OR "Relapse-Free Survival" OR "Progression-Free Survival" OR "Event-Free Survival" OR "Hazard Ratio" OR "Kaplan Meier" OR "Recurrence" OR "Time to progression")) |

| PDGFR – PUBMED |
| --- |
| (((((("receptor, platelet derived growth factor beta/analysis"[MeSH Terms] OR "receptor, platelet derived growth factor beta/blood"[MeSH Terms] OR "receptor, platelet derived growth factor beta/chemistry"[MeSH Terms] OR "receptor, platelet derived growth factor beta/drug effects"[MeSH Terms] OR "receptor, platelet derived growth factor beta/genetics"[MeSH Terms] OR "receptor, platelet derived growth factor beta/immunology"[MeSH Terms] OR "receptor, platelet derived growth factor beta/physiology"[MeSH Terms] OR "receptor, platelet derived growth factor beta/radiation effects"[MeSH Terms]) AND ("humans"[MeSH Terms] AND "english"[Language])) OR (("receptor platelet derived growth factor beta"[All Fields] OR "platelet-derived growth factor beta receptor"[All Fields] OR "platelet derived growth factor beta receptor"[All Fields] OR "PDGFR1"[All Fields] OR "PDGFR beta"[All Fields] OR "beta pdgfr"[All Fields] OR "receptor pdgf beta"[All Fields] OR "CD140b"[All Fields] OR "PDGF beta Receptor"[All Fields] OR "PDGFRB"[All Fields]) AND ("humans"[MeSH Terms] AND "english"[Language]))) AND ("humans"[MeSH Terms] AND "english"[Language])) OR (("receptor, platelet derived growth factor alpha/analysis"[MeSH Terms] OR "receptor, platelet derived growth factor alpha/blood"[MeSH Terms] OR "receptor, platelet derived growth factor alpha/chemistry"[MeSH Terms] OR "receptor, platelet derived growth factor alpha/drug effects"[MeSH Terms] OR "receptor, platelet derived growth factor alpha/genetics"[MeSH Terms] OR "receptor, platelet derived growth factor alpha/immunology"[MeSH Terms] OR "receptor, platelet derived growth factor alpha/metabolism"[MeSH Terms] OR "receptor, platelet derived growth factor alpha/pharmacology"[MeSH Terms] OR "receptor, platelet derived growth factor alpha/radiation effects"[MeSH Terms] OR "receptor, platelet derived growth factor alpha/therapeutic use"[MeSH Terms] OR ("PDGFRA"[All Fields] OR "PDGFR A"[All Fields] OR "PDGFR alpha"[All Fields] OR "pdgf r alpha"[All Fields] OR "pdgf r alpha"[All Fields] OR "receptor pdgf alpha"[All Fields] OR "CD140a"[All Fields] OR "PDGFRalpha"[All Fields] OR "PDGFR alpha"[All Fields] OR "PDGF alpha Receptor"[All Fields] OR "platelet-derived growth factor alpha receptor"[All Fields] OR "platelet derived growth factor alpha receptor"[All Fields] OR "PDGFR2"[All Fields] OR "platelet derived growth factor receptor alpha"[All Fields] OR "platelet derived growth factor receptor alpha"[All Fields] OR "PDGFR 2"[All Fields])) AND ("humans"[MeSH Terms] AND "english"[Language]))) AND ("humans"[MeSH Terms] AND "english"[Language]) AND (("Prognosis"[MeSH Terms] OR "Survival"[MeSH Terms] OR ("Prognosis"[All Fields] OR "prognostic"[All Fields] OR "Survival"[All Fields] OR "outcome"[All Fields])) AND ("humans"[MeSH Terms] AND "english"[Language])) AND (("colorectal neoplasms/blood"[MeSH Terms] OR "colorectal neoplasms/chemically induced"[MeSH Terms] OR "colorectal neoplasms/chemistry"[MeSH Terms] OR "colorectal neoplasms/classification"[MeSH Terms] OR "colorectal neoplasms/complications"[MeSH Terms] OR "colorectal neoplasms/diagnosis"[MeSH Terms] OR "colorectal neoplasms/drug therapy"[MeSH Terms] OR "colorectal neoplasms/epidemiology"[MeSH Terms] OR "colorectal neoplasms/ethnology"[MeSH Terms] OR "colorectal neoplasms/etiology"[MeSH Terms] OR "colorectal neoplasms/genetics"[MeSH Terms] OR "colorectal neoplasms/immunology"[MeSH Terms] OR "colorectal neoplasms/metabolism"[MeSH Terms] OR "colorectal neoplasms/mortality"[MeSH Terms] OR "colorectal neoplasms/pathology"[MeSH Terms] OR "colorectal neoplasms/physiopathology"[MeSH Terms] OR "colorectal neoplasms/radiotherapy"[MeSH Terms] OR "colorectal neoplasms/secondary"[MeSH Terms] OR "colorectal neoplasms/surgery"[MeSH Terms] OR "colorectal neoplasms/therapy"[MeSH Terms] OR ("colorectal neoplasm*"[All Fields] OR "colorectal tumour*"[All Fields] OR "colorectal cancer*"[All Fields] OR "colorectal adenocarcinoma"[All Fields] OR "colorectal carcinoma"[All Fields] OR "colon neoplasm*"[All Fields] OR "colon cancer*"[All Fields] OR "colon tumour*"[All Fields] OR "colon tumor*"[All Fields] OR "colon adenocarcinoma"[All Fields] OR "colon carcinoma"[All Fields] OR "rectal cancer*"[All Fields] OR "rectal neoplasm*"[All Fields] OR "rectal tumor*"[All Fields] OR "rectal tumour*"[All Fields] OR "rectal carcinoma"[All Fields] OR "rectal adenocarcinoma"[All Fields])) AND ("humans"[MeSH Terms] AND "english"[Language]))) AND ((humans[Filter]) AND (english[Filter]) AND (1993:2024[pdat])) |

| (("Receptors, Platelet-Derived Growth Factor"[Mesh] OR "Receptor, Platelet-Derived Growth Factor beta"[Mesh] OR "Receptor, Platelet-Derived Growth Factor alpha"[Mesh] OR "PDGFR α" OR "PDGFR β" OR "PDGFR") |
| --- |
| AND |
| ("Colorectal Neoplasms"[Mesh] OR "Colorectal Cancer" OR "Colon Cancer" OR "Rectal Cancer" OR "Colorectal Carcinoma" OR "Colorectal Adenocarcinoma" OR "Colonic Neoplasms" OR "Rectal Neoplasms") |
| AND |
| ("Prognosis"[Mesh] OR "Prognostic Value" OR "Prognostic Factor" OR "Survival" OR "Overall Survival" OR "Disease-Free Survival" OR "Relapse-Free Survival" OR "Progression-Free Survival" OR "Event-Free Survival" OR "Hazard Ratio" OR "Kaplan Meier" OR "Recurrence" OR "Time to progression")) |

| ACTA2 – PUBMED |
| --- |
| (("colorectal neoplasms/blood"[MeSH Terms] OR "colorectal neoplasms/chemically induced"[MeSH Terms] OR "colorectal neoplasms/chemistry"[MeSH Terms] OR "colorectal neoplasms/classification"[MeSH Terms] OR "colorectal neoplasms/complications"[MeSH Terms] OR "colorectal neoplasms/diagnosis"[MeSH Terms] OR "colorectal neoplasms/drug therapy"[MeSH Terms] OR "colorectal neoplasms/epidemiology"[MeSH Terms] OR "colorectal neoplasms/ethnology"[MeSH Terms] OR "colorectal neoplasms/etiology"[MeSH Terms] OR "colorectal neoplasms/genetics"[MeSH Terms] OR "colorectal neoplasms/immunology"[MeSH Terms] OR "colorectal neoplasms/metabolism"[MeSH Terms] OR "colorectal neoplasms/mortality"[MeSH Terms] OR "colorectal neoplasms/pathology"[MeSH Terms] OR "colorectal neoplasms/physiopathology"[MeSH Terms] OR "colorectal neoplasms/radiotherapy"[MeSH Terms] OR "colorectal neoplasms/secondary"[MeSH Terms] OR "colorectal neoplasms/surgery"[MeSH Terms] OR "colorectal neoplasms/therapy"[MeSH Terms] OR ("colorectal neoplasm*"[All Fields] OR "colorectal tumour*"[All Fields] OR "colorectal cancer*"[All Fields] OR "colorectal adenocarcinoma"[All Fields] OR "colorectal carcinoma"[All Fields] OR "colon neoplasm*"[All Fields] OR "colon cancer*"[All Fields] OR "colon tumour*"[All Fields] OR "colon tumor*"[All Fields] OR "colon adenocarcinoma"[All Fields] OR "colon carcinoma"[All Fields] OR "rectal cancer*"[All Fields] OR "rectal neoplasm*"[All Fields] OR "rectal tumor*"[All Fields] OR "rectal tumour*"[All Fields] OR "rectal carcinoma"[All Fields] OR "rectal adenocarcinoma"[All Fields])) AND ("Prognosis"[MeSH Terms] OR "Survival"[MeSH Terms] OR ("Prognosis"[All Fields] OR "prognostic"[All Fields] OR "Survival"[All Fields] OR "outcome"[All Fields])) AND ("ACTA2 protein"[All Fields] OR "alpha smooth muscle actin human"[All Fields] OR "alpha-SMA"[All Fields] OR "acta2 protein human"[Supplementary Concept])) AND ((humans[Filter]) AND (english[Filter]) AND (1997:2024[pdat])) |

| (("ACTA2 protein, human"[Supplementary Concept] OR "alpha smooth muscle actin" OR "αSMA") |
| --- |
| AND |
| ("Colorectal Neoplasms"[Mesh] OR "Colorectal Cancer" OR "Colon Cancer" OR "Rectal Cancer" OR "Colorectal Carcinoma" OR "Colorectal Adenocarcinoma" OR "Colonic Neoplasms" OR "Rectal Neoplasms") |
| AND |
| ("Prognosis"[Mesh] OR "Prognostic Value" OR "Prognostic Factor" OR "Survival" OR "Overall Survival" OR "Disease-Free Survival" OR "Relapse-Free Survival" OR "Progression-Free Survival" OR "Event-Free Survival" OR "Hazard Ratio" OR "Kaplan Meier" OR "Recurrence" OR "Time to progression")) |

| POSTN-PUBMED |
| --- |
| ("Prognosis"[MeSH Terms] OR "Survival"[MeSH Terms] OR ("Prognosis"[All Fields] OR "prognostic"[All Fields] OR "Survival"[All Fields] OR "outcome"[All Fields])) AND ("colorectal neoplasms/blood"[MeSH Terms] OR "colorectal neoplasms/chemically induced"[MeSH Terms] OR "colorectal neoplasms/chemistry"[MeSH Terms] OR "colorectal neoplasms/classification"[MeSH Terms] OR "colorectal neoplasms/complications"[MeSH Terms] OR "colorectal neoplasms/diagnosis"[MeSH Terms] OR "colorectal neoplasms/drug therapy"[MeSH Terms] OR "colorectal neoplasms/epidemiology"[MeSH Terms] OR "colorectal neoplasms/ethnology"[MeSH Terms] OR "colorectal neoplasms/etiology"[MeSH Terms] OR "colorectal neoplasms/genetics"[MeSH Terms] OR "colorectal neoplasms/immunology"[MeSH Terms] OR "colorectal neoplasms/metabolism"[MeSH Terms] OR "colorectal neoplasms/mortality"[MeSH Terms] OR "colorectal neoplasms/pathology"[MeSH Terms] OR "colorectal neoplasms/physiopathology"[MeSH Terms] OR "colorectal neoplasms/radiotherapy"[MeSH Terms] OR "colorectal neoplasms/secondary"[MeSH Terms] OR "colorectal neoplasms/surgery"[MeSH Terms] OR "colorectal neoplasms/therapy"[MeSH Terms] OR ("colorectal neoplasm*"[All Fields] OR "colorectal tumour*"[All Fields] OR "colorectal cancer*"[All Fields] OR "colorectal adenocarcinoma"[All Fields] OR "colorectal carcinoma"[All Fields] OR "colon neoplasm*"[All Fields] OR "colon cancer*"[All Fields] OR "colon tumour*"[All Fields] OR "colon tumor*"[All Fields] OR "colon adenocarcinoma"[All Fields] OR "colon carcinoma"[All Fields] OR "rectal cancer*"[All Fields] OR "rectal neoplasm*"[All Fields] OR "rectal tumor*"[All Fields] OR "rectal tumour*"[All Fields] OR "rectal carcinoma"[All Fields] OR "rectal adenocarcinoma"[All Fields])) AND ("POSTN protein"[All Fields] OR "Periostin"[All Fields] OR ("Periostin"[MeSH Terms] OR "postn protein human"[Supplementary Concept])) |

| (("Periostin"[Mesh] OR "Periostin" OR "POSTN") |
| --- |
| AND |
| ("Colorectal Neoplasms"[Mesh] OR "Colorectal Cancer" OR "Colon Cancer" OR "Rectal Cancer" OR "Colorectal Carcinoma" OR "Colorectal Adenocarcinoma" OR "Colonic Neoplasms" OR "Rectal Neoplasms") |
| AND |
| ("Prognosis"[Mesh] OR "Prognostic Value" OR "Prognostic Factor" OR "Survival" OR "Overall Survival" OR "Disease-Free Survival" OR "Relapse-Free Survival" OR "Progression-Free Survival" OR "Event-Free Survival" OR "Hazard Ratio" OR "Kaplan Meier" OR "Recurrence" OR "Time to progression")) |

| TAGLN – PUBMED |
| --- |
| (("Transgelin"[Supplementary Concept] OR "tagln2 protein human"[Supplementary Concept] OR ("Transgelin"[All Fields] OR "TAGLN protein"[All Fields] OR "transgelin protein"[All Fields] OR "SM22 protein"[All Fields])) AND ("colorectal neoplasm*"[All Fields] OR "colorectal tumour*"[All Fields] OR "colorectal cancer*"[All Fields] OR "colorectal adenocarcinoma"[All Fields] OR "colorectal carcinoma"[All Fields] OR "colon neoplasm*"[All Fields] OR "colon cancer*"[All Fields] OR "colon tumour*"[All Fields] OR "colon tumor*"[All Fields] OR "colon adenocarcinoma"[All Fields] OR "colon carcinoma"[All Fields] OR "rectal cancer*"[All Fields] OR "rectal neoplasm*"[All Fields] OR "rectal tumor*"[All Fields] OR "rectal tumour*"[All Fields] OR "rectal carcinoma"[All Fields] OR "rectal adenocarcinoma"[All Fields] OR ("colorectal neoplasms/blood"[MeSH Terms] OR "colorectal neoplasms/chemically induced"[MeSH Terms] OR "colorectal neoplasms/chemistry"[MeSH Terms] OR "colorectal neoplasms/classification"[MeSH Terms] OR "colorectal neoplasms/complications"[MeSH Terms] OR "colorectal neoplasms/diagnosis"[MeSH Terms] OR "colorectal neoplasms/drug therapy"[MeSH Terms] OR "colorectal neoplasms/epidemiology"[MeSH Terms] OR "colorectal neoplasms/ethnology"[MeSH Terms] OR "colorectal neoplasms/etiology"[MeSH Terms] OR "colorectal neoplasms/genetics"[MeSH Terms] OR "colorectal neoplasms/immunology"[MeSH Terms] OR "colorectal neoplasms/metabolism"[MeSH Terms] OR "colorectal neoplasms/mortality"[MeSH Terms] OR "colorectal neoplasms/pathology"[MeSH Terms] OR "colorectal neoplasms/physiopathology"[MeSH Terms] OR "colorectal neoplasms/radiotherapy"[MeSH Terms] OR "colorectal neoplasms/secondary"[MeSH Terms] OR "colorectal neoplasms/surgery"[MeSH Terms] OR "colorectal neoplasms/therapy"[MeSH Terms])) AND ("Prognosis"[MeSH Terms] OR "Survival"[MeSH Terms] OR ("Prognosis"[All Fields] OR "prognostic"[All Fields] OR "Survival"[All Fields] OR "outcome"[All Fields]))) AND (2001:2024[pdat]) |

| (("Tagln2 protein, human"[Supplementary Concept] OR "Transgelin 2" OR "TAGLN2") |
| --- |
| AND |
| ("Colorectal Neoplasms"[Mesh] OR "Colorectal Cancer" OR "Colon Cancer" OR "Rectal Cancer" OR "Colorectal Carcinoma" OR "Colorectal Adenocarcinoma" OR "Colonic Neoplasms" OR "Rectal Neoplasms") |
| AND |
| ("Prognosis"[Mesh] OR "Prognostic Value" OR "Prognostic Factor" OR "Survival" OR "Overall Survival" OR "Disease-Free Survival" OR "Relapse-Free Survival" OR "Progression-Free Survival" OR "Event-Free Survival" OR "Hazard Ratio" OR "Kaplan Meier" OR "Recurrence" OR "Time to progression")) |

| VIMENTIN – PUBMED |
| --- |
| (("colorectal neoplasms/blood"[MeSH Terms] OR "colorectal neoplasms/chemically induced"[MeSH Terms] OR "colorectal neoplasms/chemistry"[MeSH Terms] OR "colorectal neoplasms/classification"[MeSH Terms] OR "colorectal neoplasms/complications"[MeSH Terms] OR "colorectal neoplasms/diagnosis"[MeSH Terms] OR "colorectal neoplasms/drug therapy"[MeSH Terms] OR "colorectal neoplasms/epidemiology"[MeSH Terms] OR "colorectal neoplasms/ethnology"[MeSH Terms] OR "colorectal neoplasms/etiology"[MeSH Terms] OR "colorectal neoplasms/genetics"[MeSH Terms] OR "colorectal neoplasms/immunology"[MeSH Terms] OR "colorectal neoplasms/metabolism"[MeSH Terms] OR "colorectal neoplasms/mortality"[MeSH Terms] OR "colorectal neoplasms/pathology"[MeSH Terms] OR "colorectal neoplasms/physiopathology"[MeSH Terms] OR "colorectal neoplasms/radiotherapy"[MeSH Terms] OR "colorectal neoplasms/secondary"[MeSH Terms] OR "colorectal neoplasms/surgery"[MeSH Terms] OR "colorectal neoplasms/therapy"[MeSH Terms] OR ("colorectal neoplasm*"[All Fields] OR "colorectal tumour*"[All Fields] OR "colorectal cancer*"[All Fields] OR "colorectal adenocarcinoma"[All Fields] OR "colorectal carcinoma"[All Fields] OR "colon neoplasm*"[All Fields] OR "colon cancer*"[All Fields] OR "colon tumour*"[All Fields] OR "colon tumor*"[All Fields] OR "colon adenocarcinoma"[All Fields] OR "colon carcinoma"[All Fields] OR "rectal cancer*"[All Fields] OR "rectal neoplasm*"[All Fields] OR "rectal tumor*"[All Fields] OR "rectal tumour*"[All Fields] OR "rectal carcinoma"[All Fields] OR "rectal adenocarcinoma"[All Fields])) AND ("Prognosis"[MeSH Terms] OR "Survival"[MeSH Terms] OR ("Prognosis"[All Fields] OR "prognostic"[All Fields] OR "Survival"[All Fields] OR "outcome"[All Fields])) AND ("vimentin/analysis"[MeSH Terms] OR "vimentin/blood"[MeSH Terms] OR "vimentin/chemistry"[MeSH Terms] OR "vimentin/deficiency"[MeSH Terms] OR "vimentin/immunology"[MeSH Terms] OR "vimentin/pharmacokinetics"[MeSH Terms] OR "vimentin/pharmacology"[MeSH Terms] OR "vimentin/physiology"[MeSH Terms] OR "vimentin/radiation effects"[MeSH Terms] OR "vimentin/therapeutic use"[MeSH Terms] OR ("VIMENTIN"[All Fields] OR "VIM"[All Fields] OR "VIMENTIN protein"[All Fields]))) AND ((humans[Filter]) AND (english[Filter]) AND (1989:2024[pdat])) |

| (("Vimentin"[Mesh] OR "Vimentin" OR "VIM") |
| --- |
| AND |
| ("Colorectal Neoplasms"[Mesh] OR "Colorectal Cancer" OR "Colon Cancer" OR "Rectal Cancer" OR "Colorectal Carcinoma" OR "Colorectal Adenocarcinoma" OR "Colonic Neoplasms" OR "Rectal Neoplasms") |
| AND |
| ("Prognosis"[Mesh] OR "Prognostic Value" OR "Prognostic Factor" OR "Survival" OR "Overall Survival" OR "Disease-Free Survival" OR "Relapse-Free Survival" OR "Progression-Free Survival" OR "Event-Free Survival" OR "Hazard Ratio" OR "Kaplan Meier" OR "Recurrence" OR "Time to progression")) |

| **S100A4 – PUBMED** |
| --- |
| ("s100 calcium binding protein a4/analysis"[MeSH Terms] OR "s100 calcium binding protein a4/blood"[MeSH Terms] OR "s100 calcium binding protein a4/chemistry"[MeSH Terms] OR "s100 calcium binding protein a4/immunology"[MeSH Terms] OR "s100 calcium binding protein a4/isolation and purification"[MeSH Terms] OR "s100 calcium binding protein a4/pharmacology"[MeSH Terms] OR "s100 calcium binding protein a4/physiology"[MeSH Terms] OR (("S100A4 protein"[All Fields] OR "s100a4 protein human"[All Fields] OR "S100 Calcium Binding Protein A4"[All Fields]) AND ("humans"[MeSH Terms] AND "english"[Language]))) AND ("Prognosis"[MeSH Terms] OR "Survival"[MeSH Terms] OR ("Prognosis"[All Fields] OR "prognostic"[All Fields] OR "Survival"[All Fields] OR "outcome"[All Fields])) AND ("colorectal neoplasms/blood"[MeSH Terms] OR "colorectal neoplasms/chemically induced"[MeSH Terms] OR "colorectal neoplasms/chemistry"[MeSH Terms] OR "colorectal neoplasms/classification"[MeSH Terms] OR "colorectal neoplasms/complications"[MeSH Terms] OR "colorectal neoplasms/diagnosis"[MeSH Terms] OR "colorectal neoplasms/drug therapy"[MeSH Terms] OR "colorectal neoplasms/epidemiology"[MeSH Terms] OR "colorectal neoplasms/ethnology"[MeSH Terms] OR "colorectal neoplasms/etiology"[MeSH Terms] OR "colorectal neoplasms/genetics"[MeSH Terms] OR "colorectal neoplasms/immunology"[MeSH Terms] OR "colorectal neoplasms/metabolism"[MeSH Terms] OR "colorectal neoplasms/mortality"[MeSH Terms] OR "colorectal neoplasms/pathology"[MeSH Terms] OR "colorectal neoplasms/physiopathology"[MeSH Terms] OR "colorectal neoplasms/radiotherapy"[MeSH Terms] OR "colorectal neoplasms/secondary"[MeSH Terms] OR "colorectal neoplasms/surgery"[MeSH Terms] OR "colorectal neoplasms/therapy"[MeSH Terms] OR ("colorectal neoplasm*"[All Fields] OR "colorectal tumour*"[All Fields] OR "colorectal cancer*"[All Fields] OR "colorectal adenocarcinoma"[All Fields] OR "colorectal carcinoma"[All Fields] OR "colon neoplasm*"[All Fields] OR "colon cancer*"[All Fields] OR "colon tumour*"[All Fields] OR "colon tumor*"[All Fields] OR "colon adenocarcinoma"[All Fields] OR "colon carcinoma"[All Fields] OR "rectal cancer*"[All Fields] OR "rectal neoplasm*"[All Fields] OR "rectal tumor*"[All Fields] OR "rectal tumour*"[All Fields] OR "rectal carcinoma"[All Fields] OR "rectal adenocarcinoma"[All Fields])) |

| (("S100 Calcium-Binding Protein A4"[Mesh] OR "S100A4") |
| --- |
| AND |
| ("Colorectal Neoplasms"[Mesh] OR "Colorectal Cancer" OR "Colon Cancer" OR "Rectal Cancer" OR "Colorectal Carcinoma" OR "Colorectal Adenocarcinoma" OR "Colonic Neoplasms" OR "Rectal Neoplasms") |
| AND |
| ("Prognosis"[Mesh] OR "Prognostic Value" OR "Prognostic Factor" OR "Survival" OR "Overall Survival" OR "Disease-Free Survival" OR "Relapse-Free Survival" OR "Progression-Free Survival" OR "Event-Free Survival" OR "Hazard Ratio" OR "Kaplan Meier" OR "Recurrence" OR "Time to progression")) |

| GENERAL SEARCH – PUBMED |
| --- |
| **(((( "Cancer-Associated Fibroblasts/classification"[Mesh] OR "Cancer-Associated Fibroblasts/cytology"[Mesh] OR "Cancer-Associated Fibroblasts/drug effects"[Mesh] OR "Cancer-Associated Fibroblasts/immunology"[Mesh] OR "Cancer-Associated Fibroblasts/microbiology"[Mesh] OR "Cancer-Associated Fibroblasts/pathology"[Mesh] OR "Cancer-Associated Fibroblasts/physiology"[Mesh] OR "Cancer-Associated Fibroblasts/radiation effects"[Mesh] )) OR (("Cancer-Associated Fibroblast" OR "Cancer-Associated Fibroblasts" OR "Fibroblast, Cancer-Associated" OR "Fibroblasts, Cancer-Associated" OR "Cancer Associated Fibroblasts" OR "Cancer Associated Fibroblast" OR "Fibroblast, Cancer Associated" OR "Fibroblasts, Cancer Associated" OR "Tumor-Associated Fibroblasts" OR "Fibroblasts, Tumor-Associated" OR "Fibroblast, Tumor-Associated" OR "Tumor-Associated Fibroblast" OR "Tumor Associated Fibroblasts"))) AND (("Colorectal Neoplasms/blood"[Mesh] OR "Colorectal Neoplasms/chemically induced"[Mesh] OR "Colorectal Neoplasms/chemistry"[Mesh] OR "Colorectal Neoplasms/complications"[Mesh] OR "Colorectal Neoplasms/diagnosis"[Mesh] OR "Colorectal Neoplasms/drug therapy"[Mesh] OR "Colorectal Neoplasms/epidemiology"[Mesh] OR "Colorectal Neoplasms/ethnology"[Mesh] OR "Colorectal Neoplasms/etiology"[Mesh] OR "Colorectal Neoplasms/genetics"[Mesh] OR "Colorectal Neoplasms/immunology"[Mesh] OR "Colorectal Neoplasms/metabolism"[Mesh] OR "Colorectal Neoplasms/mortality"[Mesh] OR "Colorectal Neoplasms/pathology"[Mesh] OR "Colorectal Neoplasms/physiopathology"[Mesh] OR "Colorectal Neoplasms/radiotherapy"[Mesh] OR "Colorectal Neoplasms/secondary"[Mesh] OR "Colorectal Neoplasms/surgery"[Mesh] OR "Colorectal Neoplasms/therapy"[Mesh] ) OR "Colorectal Neoplasm*" OR "Colorectal tumour*" OR "Colorectal tumour*" OR "Colorectal cancer*" OR " colorectal adenocarcinoma" OR "colorectal carcinoma" OR "colon neoplasm*" OR "colon cancer*" OR "colon tumour*" OR "colon tumor*" OR " colon adenocarcinoma" OR "colon carcinoma" OR "rectal cancer*" OR "Rectal neoplasm*" OR "rectal tumor*" OR "rectal tumour" OR "rectal carcinoma" OR "rectal adenocarcinoma" AND (1892:2024[pdat]))) AND (("Prognosis"[Mesh] OR "prognosis" OR "prognostic" OR "survival"))** Filters: **English, Humans, Adult: 19+ years, from 2006 – 2024** |

| COL11A1 -PUBMED |
| --- |
| ("col11a1 protein human"[Supplementary Concept] OR ("COL11A1"[All Fields] OR "collagen type XI alpha 1 chain"[All Fields] OR (("collagen"[MeSH Terms] OR "collagen"[All Fields] OR "collagens"[All Fields] OR "collagen s"[All Fields] OR "collagenation"[All Fields] OR "collagene"[All Fields] OR "collageneous"[All Fields] OR "collagenic"[All Fields] OR "collagenization"[All Fields] OR "collagenized"[All Fields] OR "collagenous"[All Fields]) AND "type"[All Fields] AND "11"[All Fields] AND ("alpha"[All Fields] OR "alpha s"[All Fields] OR "alphas"[All Fields]) AND "1"[All Fields] AND ("chain"[All Fields] OR "chain s"[All Fields] OR "chains"[All Fields])) OR (("collagen"[MeSH Terms] OR "collagen"[All Fields] OR "collagens"[All Fields] OR "collagen s"[All Fields] OR "collagenation"[All Fields] OR "collagene"[All Fields] OR "collageneous"[All Fields] OR "collagenic"[All Fields] OR "collagenization"[All Fields] OR "collagenized"[All Fields] OR "collagenous"[All Fields]) AND "xi"[All Fields] AND "alpha 1"[All Fields] AND ("peptides"[MeSH Terms] OR "peptides"[All Fields] OR "polypeptide"[All Fields] OR "polypeptides"[All Fields] OR "polypeptid"[All Fields] OR "polypeptide s"[All Fields] OR "polypeptidic"[All Fields])) OR ("coll6"[All Fields] AND ("protein s"[All Fields] OR "proteinous"[All Fields] OR "proteins"[MeSH Terms] OR "proteins"[All Fields] OR "protein"[All Fields])) OR "DFNA37"[All Fields] OR (("collagen type xi"[MeSH Terms] OR ("collagen"[All Fields] AND "type"[All Fields] AND "xi"[All Fields]) OR "collagen type xi"[All Fields] OR "collagen type xi"[All Fields]) AND ("alpha"[All Fields] OR "alpha s"[All Fields] OR "alphas"[All Fields]) AND "1"[All Fields] AND ("human s"[All Fields] OR "humans"[MeSH Terms] OR "humans"[All Fields] OR "human"[All Fields])) OR "STL2"[All Fields])) AND ("Prognosis"[MeSH Terms] OR "Survival"[MeSH Terms] OR ("Prognosis"[All Fields] OR "prognostic"[All Fields] OR "Survival"[All Fields] OR "outcome"[All Fields])) AND ("colorectal neoplasms/blood"[MeSH Terms] OR "colorectal neoplasms/chemically induced"[MeSH Terms] OR "colorectal neoplasms/chemistry"[MeSH Terms] OR "colorectal neoplasms/classification"[MeSH Terms] OR "colorectal neoplasms/complications"[MeSH Terms] OR "colorectal neoplasms/diagnosis"[MeSH Terms] OR "colorectal neoplasms/drug therapy"[MeSH Terms] OR "colorectal neoplasms/epidemiology"[MeSH Terms] OR "colorectal neoplasms/ethnology"[MeSH Terms] OR "colorectal neoplasms/etiology"[MeSH Terms] OR "colorectal neoplasms/genetics"[MeSH Terms] OR "colorectal neoplasms/immunology"[MeSH Terms] OR "colorectal neoplasms/metabolism"[MeSH Terms] OR "colorectal neoplasms/mortality"[MeSH Terms] OR "colorectal neoplasms/pathology"[MeSH Terms] OR "colorectal neoplasms/physiopathology"[MeSH Terms] OR "colorectal neoplasms/radiotherapy"[MeSH Terms] OR "colorectal neoplasms/secondary"[MeSH Terms] OR "colorectal neoplasms/surgery"[MeSH Terms] OR "colorectal neoplasms/therapy"[MeSH Terms] OR ("colorectal neoplasm*"[All Fields] OR "colorectal tumour*"[All Fields] OR "colorectal cancer*"[All Fields] OR "colorectal adenocarcinoma"[All Fields] OR "colorectal carcinoma"[All Fields] OR "colon neoplasm*"[All Fields] OR "colon cancer*"[All Fields] OR "colon tumour*"[All Fields] OR "colon tumor*"[All Fields] OR "colon adenocarcinoma"[All Fields] OR "colon carcinoma"[All Fields] OR "rectal cancer*"[All Fields] OR "rectal neoplasm*"[All Fields] OR "rectal tumor*"[All Fields] OR "rectal tumour*"[All Fields] OR "rectal carcinoma"[All Fields] OR "rectal adenocarcinoma"[All Fields])) |

| (("COL11A1 protein, human"[Supplementary Concept] OR "Collagen type XI Alpha 1 chain" OR "COL11A1") |
| --- |
| AND |
| ("Colorectal Neoplasms"[Mesh] OR "Colorectal Cancer" OR "Colon Cancer" OR "Rectal Cancer" OR "Colorectal Carcinoma" OR "Colorectal Adenocarcinoma" OR "Colonic Neoplasms" OR "Rectal Neoplasms") |
| AND |
| ("Prognosis"[Mesh] OR "Prognostic Value" OR "Prognostic Factor" OR "Survival" OR "Overall Survival" OR "Disease-Free Survival" OR "Relapse-Free Survival" OR "Progression-Free Survival" OR "Event-Free Survival" OR "Hazard Ratio" OR "Kaplan Meier" OR "Recurrence" OR "Time to progression")) |

| COL1A1-PUBMED |
| --- |
| ("collagen type i, alpha 1 chain/analysis"[MeSH Terms] OR "collagen type i, alpha 1 chain/genetics"[MeSH Terms] OR "collagen type i, alpha 1 chain/pharmacology"[MeSH Terms] OR ("Collagen Type I Alpha 1 Chain"[All Fields] OR "COL1A1 Protein"[All Fields] OR "COL1A1"[All Fields] OR "Type I Collagen alpha 1"[All Fields] OR "collagen alpha 1 i"[All Fields] OR "pro alpha1 i collagen"[All Fields] OR "pro alpha1 i collagen"[All Fields] OR "Pro alpha 1 Collagen"[All Fields] OR "Pro alpha 1 Collagen"[All Fields] OR "Pro alpha 1 Collagen"[All Fields] OR "procollagen alpha1 i"[All Fields])) AND ("colorectal neoplasms/blood"[MeSH Terms] OR "colorectal neoplasms/chemically induced"[MeSH Terms] OR "colorectal neoplasms/chemistry"[MeSH Terms] OR "colorectal neoplasms/classification"[MeSH Terms] OR "colorectal neoplasms/complications"[MeSH Terms] OR "colorectal neoplasms/diagnosis"[MeSH Terms] OR "colorectal neoplasms/drug therapy"[MeSH Terms] OR "colorectal neoplasms/epidemiology"[MeSH Terms] OR "colorectal neoplasms/ethnology"[MeSH Terms] OR "colorectal neoplasms/etiology"[MeSH Terms] OR "colorectal neoplasms/genetics"[MeSH Terms] OR "colorectal neoplasms/immunology"[MeSH Terms] OR "colorectal neoplasms/metabolism"[MeSH Terms] OR "colorectal neoplasms/mortality"[MeSH Terms] OR "colorectal neoplasms/pathology"[MeSH Terms] OR "colorectal neoplasms/physiopathology"[MeSH Terms] OR "colorectal neoplasms/radiotherapy"[MeSH Terms] OR "colorectal neoplasms/secondary"[MeSH Terms] OR "colorectal neoplasms/surgery"[MeSH Terms] OR "colorectal neoplasms/therapy"[MeSH Terms] OR ("colorectal neoplasm*"[All Fields] OR "colorectal tumour*"[All Fields] OR "colorectal cancer*"[All Fields] OR "colorectal adenocarcinoma"[All Fields] OR "colorectal carcinoma"[All Fields] OR "colon neoplasm*"[All Fields] OR "colon cancer*"[All Fields] OR "colon tumour*"[All Fields] OR "colon tumor*"[All Fields] OR "colon adenocarcinoma"[All Fields] OR "colon carcinoma"[All Fields] OR "rectal cancer*"[All Fields] OR "rectal neoplasm*"[All Fields] OR "rectal tumor*"[All Fields] OR "rectal tumour*"[All Fields] OR "rectal carcinoma"[All Fields] OR "rectal adenocarcinoma"[All Fields])) AND ("Prognosis"[MeSH Terms] OR "Survival"[MeSH Terms] OR ("Prognosis"[All Fields] OR "prognostic"[All Fields] OR "Survival"[All Fields] OR "outcome"[All Fields])) |

| (("Collagen Type I, alpha 1 Chain"[Mesh] OR "Collagen type I Alpha 1 chain" OR "COL1A1") |
| --- |
| AND |
| ("Colorectal Neoplasms"[Mesh] OR "Colorectal Cancer" OR "Colon Cancer" OR "Rectal Cancer" OR "Colorectal Carcinoma" OR "Colorectal Adenocarcinoma" OR "Colonic Neoplasms" OR "Rectal Neoplasms") |
| AND |
| ("Prognosis"[Mesh] OR "Prognostic Value" OR "Prognostic Factor" OR "Survival" OR "Overall Survival" OR "Disease-Free Survival" OR "Relapse-Free Survival" OR "Progression-Free Survival" OR "Event-Free Survival" OR "Hazard Ratio" OR "Kaplan Meier" OR "Recurrence" OR "Time to progression")) |

| DECORIN – PUBMED |
| --- |
| ("decorin receptor"[Supplementary Concept] OR "dcn protein human"[Supplementary Concept] OR ("decorin/blood"[MeSH Terms] OR "decorin/drug effects"[MeSH Terms] OR "decorin/genetics"[MeSH Terms] OR "decorin/immunology"[MeSH Terms] OR "decorin/pharmacology"[MeSH Terms] OR "decorin/physiology"[MeSH Terms] OR "decorin/therapeutic use"[MeSH Terms]) OR ("Decorin"[All Fields] OR "DCN"[All Fields] OR "DSPG-II"[All Fields] OR "Bone Proteoglycan II"[All Fields] OR ("Decorin"[MeSH Terms] OR "Decorin"[All Fields] OR ("proteoglycan"[All Fields] AND "ii"[All Fields] AND "bone"[All Fields])))) AND ("Prognosis"[MeSH Terms] OR "Survival"[MeSH Terms] OR ("Prognosis"[All Fields] OR "prognostic"[All Fields] OR "Survival"[All Fields] OR "outcome"[All Fields])) AND ("colorectal neoplasms/blood"[MeSH Terms] OR "colorectal neoplasms/chemically induced"[MeSH Terms] OR "colorectal neoplasms/chemistry"[MeSH Terms] OR "colorectal neoplasms/classification"[MeSH Terms] OR "colorectal neoplasms/complications"[MeSH Terms] OR "colorectal neoplasms/diagnosis"[MeSH Terms] OR "colorectal neoplasms/drug therapy"[MeSH Terms] OR "colorectal neoplasms/epidemiology"[MeSH Terms] OR "colorectal neoplasms/ethnology"[MeSH Terms] OR "colorectal neoplasms/etiology"[MeSH Terms] OR "colorectal neoplasms/genetics"[MeSH Terms] OR "colorectal neoplasms/immunology"[MeSH Terms] OR "colorectal neoplasms/metabolism"[MeSH Terms] OR "colorectal neoplasms/mortality"[MeSH Terms] OR "colorectal neoplasms/pathology"[MeSH Terms] OR "colorectal neoplasms/physiopathology"[MeSH Terms] OR "colorectal neoplasms/radiotherapy"[MeSH Terms] OR "colorectal neoplasms/secondary"[MeSH Terms] OR "colorectal neoplasms/surgery"[MeSH Terms] OR "colorectal neoplasms/therapy"[MeSH Terms] OR ("colorectal neoplasm*"[All Fields] OR "colorectal tumour*"[All Fields] OR "colorectal cancer*"[All Fields] OR "colorectal adenocarcinoma"[All Fields] OR "colorectal carcinoma"[All Fields] OR "colon neoplasm*"[All Fields] OR "colon cancer*"[All Fields] OR "colon tumour*"[All Fields] OR "colon tumor*"[All Fields] OR "colon adenocarcinoma"[All Fields] OR "colon carcinoma"[All Fields] OR "rectal cancer*"[All Fields] OR "rectal neoplasm*"[All Fields] OR "rectal tumor*"[All Fields] OR "rectal tumour*"[All Fields] OR "rectal carcinoma"[All Fields] OR "rectal adenocarcinoma"[All Fields])) |
|  |

| (("Decorin"[Mesh] OR "Decorin" OR "DCN") |
| --- |
| AND |
| ("Colorectal Neoplasms"[Mesh] OR "Colorectal Cancer" OR "Colon Cancer" OR "Rectal Cancer" OR "Colorectal Carcinoma" OR "Colorectal Adenocarcinoma" OR "Colonic Neoplasms" OR "Rectal Neoplasms") |
| AND |
| ("Prognosis"[Mesh] OR "Prognostic Value" OR "Prognostic Factor" OR "Survival" OR "Overall Survival" OR "Disease-Free Survival" OR "Relapse-Free Survival" OR "Progression-Free Survival" OR "Event-Free Survival" OR "Hazard Ratio" OR "Kaplan Meier" OR "Recurrence" OR "Time to progression")) |
