# Supplementary material for: Cancer-associated fibroblasts and their prognostic role in colorectal cancer: review and meta-analysis
Source: Front Oncol. 2025 Dec 10;15:1635055. doi: 10.3389/fonc.2025.1635055 (PMC12727558; doi:10.3389/fonc.2025.1635055)
Supplement: Supplementary file 4 [file Table2.docx]

| TNC-EMBASE |
| --- |
| #1 AND #2 AND #3 AND [article]/lim AND [english]/lim AND [<1966-2024]/py |
| [53](https://www.embase.com.ezproxy.medgrid.eu/)  **#4**  #1 AND #2 AND #3 |
| [96](https://www.embase.com.ezproxy.medgrid.eu/)  **#3**  **'tenascin'**/exp OR **'glioma mesenchymal extracellular matrix antigen'** OR **'glycoprotein j1'** OR **'j1 glycoprotein'** OR **'myotendinous antigen'** OR **'tenascin'** OR **'tnc'** OR **'cytotactin'** OR **'hexabrachion'** OR **'tenascin-c'** OR **'tenascin c'** OR **'j1-200-220'** OR **'tn-c'** |
| [13,290](https://www.embase.com.ezproxy.medgrid.eu/)  **#2**  **'prognosis'**/exp OR **'survival'**/exp OR **'prognosis'** OR **'prognostic'** OR **'survival'** OR **'outcome'** |
| [6,388,488](https://www.embase.com.ezproxy.medgrid.eu/)  **#1**  **'colorectal cancer'**/exp OR **'cancer of colon and rectum'** OR **'cancer of rectum and colon'** OR **'cancer of the colon and rectum'** OR **'cancer of the colon and the rectum'** OR **'cancer of the rectum and colon'** OR **'cancer of the rectum and the colon'** OR **'colo-rectal cancer'** OR **'colo-rectal carcinogenesis'** OR **'colo-rectal malignancies'** OR **'colo-rectal malignancy'** OR **'colorectal cancerogenesis'** OR **'colorectal carcinogenesis'** OR **'colorectal malignancies'** OR **'colorectal malignancy'** OR **'malignancies of the colon and rectum'** OR **'malignancy of colon and rectum'** OR **'malignancy of the colon and rectum'** OR **'recto-colonic cancer'** OR **'rectocolonic cancer'** OR **'colorectal cancer'** |

Tabel 2. Search and retrieval strategy Embase

| MMP9,MMP2 – Embase |
| --- |
| #5 AND [embase]/lim NOT ([embase]/lim AND [medline]/lim) AND [english]/lim  [106](https://www.embase.com.ezproxy.medgrid.eu/)  **#6** |
| #5 AND [embase]/lim NOT ([embase]/lim AND [medline]/lim)  [121](https://www.embase.com.ezproxy.medgrid.eu/)  **#5** |
| #4 AND **'article'**/it  [511](https://www.embase.com.ezproxy.medgrid.eu/)  **#4** |
| #1 AND #2 AND #3  [941](https://www.embase.com.ezproxy.medgrid.eu/)  **#3** |
| **'prognosis'**/exp OR **'survival'**/exp OR **'prognosis'** OR **'prognostic'** OR **'survival'** OR **'outcome'**  [6,277,933](https://www.embase.com.ezproxy.medgrid.eu/)  **#2** |
| **'colorectal cancer'**/exp OR **'cancer of colon and rectum'** OR **'cancer of rectum and colon'** OR **'cancer of the colon and rectum'** OR **'cancer of the colon and the rectum'** OR **'cancer of the rectum and colon'** OR **'cancer of the rectum and the colon'** OR **'colo-rectal cancer'** OR **'colo-rectal carcinogenesis'** OR **'colo-rectal malignancies'** OR **'colo-rectal malignancy'** OR **'colorectal cancerogenesis'** OR **'colorectal carcinogenesis'** OR **'colorectal malignancies'** OR **'colorectal malignancy'** OR **'malignancies of the colon and rectum'** OR **'malignancy of colon and rectum'** OR **'malignancy of the colon and rectum'** OR **'recto-colonic cancer'** OR **'rectocolonic cancer'** OR **'colorectal cancer'**  [460,134](https://www.embase.com.ezproxy.medgrid.eu/)  **#1**  **'mmp2'**/exp OR **'mmp2'** OR **'mmp2 metalloproteinase'** OR **'72-kda type iv collagenase'** OR **'72 kda type iv collagenase'** OR **'matrix metalloproteinase-2'**/exp OR **'matrix metalloproteinase-2'** OR **'72-kda gelatinase'** OR **'72 kda gelatinase'** OR **'gelatinase, 72-kda'** OR **'mmp-2 metalloproteinase'** OR **'metalloproteinase, mmp-2'** OR **'mmp 2 metalloproteinase'** OR **'metalloproteinase, mmp2'** OR **'gelatinase a'**/exp OR **'gelatinase a'** |

| CXCL12 – EMBASE |
| --- |
| #4 AND (**'clinical study'**/de OR **'clinical trial'**/de OR **'clinical trial topic'**/de OR **'cohort analysis'**/de OR **'comparative study'**/de OR **'controlled clinical trial'**/de OR **'controlled study'**/de OR **'diagnostic test accuracy study'**/de OR **'human'**/de OR **'major clinical study'**/de OR **'meta analysis'**/de OR **'multicenter study'**/de OR **'multicenter study topic'**/de OR **'phase 1 clinical trial'**/de OR **'phase 1 clinical trial topic'**/de OR **'phase 2 clinical trial'**/de OR **'phase 2 clinical trial topic'**/de OR **'phase 3 clinical trial topic'**/de OR **'prospective study'**/de OR **'randomized controlled trial'**/de OR **'randomized controlled trial topic'**/de OR **'retrospective study'**/de OR **'systematic review'**/de OR **'validation process'**/de) AND [article]/lim AND [english]/lim AND [<1966-2024]/py  [176](https://www.embase.com.ezproxy.medgrid.eu/)  **#5** |
| #4 AND (**'clinical study'**/de OR **'clinical trial'**/de OR **'clinical trial topic'**/de OR **'cohort analysis'**/de OR **'comparative study'**/de OR **'controlled clinical trial'**/de OR **'controlled study'**/de OR **'diagnostic test accuracy study'**/de OR **'human'**/de OR **'major clinical study'**/de OR **'meta analysis'**/de OR **'multicenter study'**/de OR **'multicenter study topic'**/de OR **'phase 1 clinical trial'**/de OR **'phase 1 clinical trial topic'**/de OR **'phase 2 clinical trial'**/de OR **'phase 2 clinical trial topic'**/de OR **'phase 3 clinical trial topic'**/de OR **'prospective study'**/de OR **'randomized controlled trial'**/de OR **'randomized controlled trial topic'**/de OR **'retrospective study'**/de OR **'systematic review'**/de OR **'validation process'**/de)  [421](https://www.embase.com.ezproxy.medgrid.eu/)  **#4** |
| #1 AND #2 AND #3  [432](https://www.embase.com.ezproxy.medgrid.eu/)  **#3** |
| **'stromal cell derived factor 1'**/exp OR **'chemokine cxcl12'** OR **'cxc chemokine ligand 12'** OR **'cxcl12 chemokine'** OR **'pbsf'** OR **'pre b cell growth stimulating factor'** OR **'protein scyb12'** OR **'scyb12 protein'** OR **'sdf 1'** OR **'small inducible cytokine b12'** OR **'stromal derived factor 1'** OR **'stromal cell derived factor 1'**  [25,530](https://www.embase.com.ezproxy.medgrid.eu/)  **#2** |
| **'prognosis'**/exp OR **'survival'**/exp OR **'prognosis'** OR **'prognostic'** OR **'survival'** OR **'outcome'** |
| [6,388,488](https://www.embase.com.ezproxy.medgrid.eu/)  **#1**  **'colorectal cancer'**/exp OR **'cancer of colon and rectum'** OR **'cancer of rectum and colon'** OR **'cancer of the colon and rectum'** OR **'cancer of the colon and the rectum'** OR **'cancer of the rectum and colon'** OR **'cancer of the rectum and the colon'** OR **'colo-rectal cancer'** OR **'colo-rectal carcinogenesis'** OR **'colo-rectal malignancies'** OR **'colo-rectal malignancy'** OR **'colorectal cancerogenesis'** OR **'colorectal carcinogenesis'** OR **'colorectal malignancies'** OR **'colorectal malignancy'** OR **'malignancies of the colon and rectum'** OR **'malignancy of colon and rectum'** OR **'malignancy of the colon and rectum'** OR **'recto-colonic cancer'** OR **'rectocolonic cancer'** OR **'colorectal cancer'** |

| FAP-Embase |
| --- |
| #1 AND #2 AND #3 AND [<1966-2024]/py AND [article]/lim AND [english]/lim |
| [52](https://www.embase.com.ezproxy.medgrid.eu/)  **#5**  #1 AND #2 AND #3 AND [<1966-2024]/py |
| [80](https://www.embase.com.ezproxy.medgrid.eu/)  **#4**  #1 AND #2 AND #3 |
| [81](https://www.embase.com.ezproxy.medgrid.eu/)  **#3**  **'seprase'**/exp OR **'fibroblast activation protein alpha'** OR **'seprase'** OR **'fibroblast activation protein'**/exp |
| [2,145](https://www.embase.com.ezproxy.medgrid.eu/)  **#2**  **'prognosis'**/exp OR **'survival'**/exp OR **'prognosis'** OR **'prognostic'** OR **'survival'** OR **'outcome'** |
| [6,388,488](https://www.embase.com.ezproxy.medgrid.eu/)  **#1**  **'colorectal cancer'**/exp OR **'cancer of colon and rectum'** OR **'cancer of rectum and colon'** OR **'cancer of the colon and rectum'** OR **'cancer of the colon and the rectum'** OR **'cancer of the rectum and colon'** OR **'cancer of the rectum and the colon'** OR **'colo-rectal cancer'** OR **'colo-rectal carcinogenesis'** OR **'colo-rectal malignancies'** OR **'colo-rectal malignancy'** OR **'colorectal cancerogenesis'** OR **'colorectal carcinogenesis'** OR **'colorectal malignancies'** OR **'colorectal malignancy'** OR **'malignancies of the colon and rectum'** OR **'malignancy of colon and rectum'** OR **'malignancy of the colon and rectum'** OR **'recto-colonic cancer'** OR **'rectocolonic cancer'** OR **'colorectal cancer'** |

| PDPN- EMBASE |
| --- |
| #1 AND #2 AND #3 AND [article]/lim AND [english]/lim AND [<1966-2024]/py |
| [48](https://www.embase.com.ezproxy.medgrid.eu/)  **#4**  #1 AND #2 AND #3 |
| [105](https://www.embase.com.ezproxy.medgrid.eu/)  **#3**  **'podoplanin'**/exp OR **'pdpn'** OR **'t1alpha protein'** OR **'t1 alpha protein'** OR **'podoplanin'** OR **'gp38 protein'** OR **'aggrus protein'** |
| [5,111](https://www.embase.com.ezproxy.medgrid.eu/)  **#2**  **'prognosis'**/exp OR **'survival'**/exp OR **'prognosis'** OR **'prognostic'** OR **'survival'** OR **'outcome'** |
| [6,388,488](https://www.embase.com.ezproxy.medgrid.eu/)  **#1**  **'colorectal cancer'**/exp OR **'cancer of colon and rectum'** OR **'cancer of rectum and colon'** OR **'cancer of the colon and rectum'** OR **'cancer of the colon and the rectum'** OR **'cancer of the rectum and colon'** OR **'cancer of the rectum and the colon'** OR **'colo-rectal cancer'** OR **'colo-rectal carcinogenesis'** OR **'colo-rectal malignancies'** OR **'colo-rectal malignancy'** OR **'colorectal cancerogenesis'** OR **'colorectal carcinogenesis'** OR **'colorectal malignancies'** OR **'colorectal malignancy'** OR **'malignancies of the colon and rectum'** OR **'malignancy of colon and rectum'** OR **'malignancy of the colon and rectum'** OR **'recto-colonic**  **cancer'** OR **'rectocolonic cancer'** OR **'colorectal cancer'** |

| CD163 – EMBASE |
| --- |
| #1 AND #2 AND #3 AND [humans]/lim AND [english]/lim AND [<1966-2024]/py AND [article]/lim |
| [202](https://www.embase.com.ezproxy.medgrid.eu/)  **#5**  #1 AND #2 AND #3 AND [humans]/lim AND [english]/lim AND [<1966-2024]/py |
| [317](https://www.embase.com.ezproxy.medgrid.eu/)  **#4**  #1 AND #2 AND #3 |
| [356](https://www.embase.com.ezproxy.medgrid.eu/)  **#3**  **'cd163 antigen'**/exp OR **'cd163'** OR **'cd163 protein'** OR **'rm3-1 antigen'** OR **'rm3 1 antigen'** OR **'cd163b'** OR **'cd163 antigen'** |
| [16,451](https://www.embase.com.ezproxy.medgrid.eu/)  **#2**  **'prognosis'**/exp OR **'survival'**/exp OR **'prognosis'** OR **'prognostic'** OR **'survival'** OR **'outcome'** |
| [6,388,488](https://www.embase.com.ezproxy.medgrid.eu/)  **#1**  **'colorectal cancer'**/exp OR **'cancer of colon and rectum'** OR **'cancer of rectum and colon'** OR **'cancer of the colon and rectum'** OR **'cancer of the colon and the rectum'** OR **'cancer of the rectum and colon'** OR **'cancer of the rectum and the colon'** OR **'colo-rectal cancer'** OR **'colo-rectal carcinogenesis'** OR **'colo-rectal malignancies'** OR **'colo-rectal malignancy'** OR **'colorectal cancerogenesis'** OR **'colorectal carcinogenesis'** OR **'colorectal malignancies'** OR **'colorectal malignancy'** OR **'malignancies of the colon and rectum'** OR **'malignancy of colon and rectum'** OR **'malignancy of the colon and rectum'** OR **'recto-colonic cancer'** OR **'rectocolonic cancer'** OR **'colorectal cancer'** |

| PDGFR-EMBASE |
| --- |
| 1 AND #2 AND #3 AND [article]/lim AND [humans]/lim AND [english]/lim AND [<1966-2024]/py |
| [145](https://www.embase.com.ezproxy.medgrid.eu/)  **#4**  #1 AND #2 AND #3 |
| [419](https://www.embase.com.ezproxy.medgrid.eu/)  **#3**  **'platelet derived growth factor receptor'**/exp OR **'pdgf receptor'** OR **'receptors, platelet derived growth factor'** OR **'receptors, platelet-derived growth factor'** OR **'thrombocyte growth factor receptor'** OR **'platelet derived growth factor receptor'** OR **'platelet-derived growth factor receptor'** OR **'platelet-derived growth factor alpha receptor'** OR **'platelet derived growth factor alpha receptor'** OR **'platelet-derived growth factor beta receptor'** OR **'plateletderived growth factor beta receptor'** |
| [24,183](https://www.embase.com.ezproxy.medgrid.eu/)  **#2**  **'prognosis'**/exp OR **'survival'**/exp OR **'prognosis'** OR **'prognostic'** OR **'survival'** OR **'outcome'** |
| [6,388,488](https://www.embase.com.ezproxy.medgrid.eu/)  **#1**  **'colorectal cancer'**/exp OR **'cancer of colon and rectum'** OR **'cancer of rectum and colon'** OR **'cancer of the colon and rectum'** OR **'cancer of the colon and the rectum'** OR **'cancer of the rectum and colon'** OR **'cancer of the rectum and the colon'** OR **'colo-rectal cancer'** OR **'colo-rectal carcinogenesis'** OR **'colo-rectal malignancies'** OR **'colo-rectal malignancy'** OR **'colorectal cancerogenesis'** OR **'colorectal carcinogenesis'** OR **'colorectal malignancies'** OR **'colorectal malignancy'** OR **'malignancies of the colon and rectum'** OR **'malignancy of colon and rectum'** OR **'malignancy of the colon and rectum'** OR **'recto-colonic cancer'** OR **'rectocolonic cancer'** OR **'colorectal cancer'** |

| ACTA2-EMBASE |
| --- |
| #1 AND #2 AND #3 AND [article]/lim AND [english]/lim AND [humans]/lim AND [<1966-2024]/py |
| [129](https://www.embase.com.ezproxy.medgrid.eu/)  **#4**  #1 AND #2 AND #3 |
| [211](https://www.embase.com.ezproxy.medgrid.eu/)  **#3**  **'alpha smooth muscle actin'**/exp OR **'smooth muscle alpha actin'** OR **'alpha smooth muscle actin'** OR **'acta2 protein human'**/exp OR **'alpha-sma'** OR **'acta2'** OR **'alpha 2 actin'** |
| [38,305](https://www.embase.com.ezproxy.medgrid.eu/)  **#2**  **'prognosis'**/exp OR **'survival'**/exp OR **'prognosis'** OR **'prognostic'** OR **'survival'** OR **'outcome'** |
| [6,386,321](https://www.embase.com.ezproxy.medgrid.eu/)  **#1**  **'colorectal cancer'**/exp OR **'cancer of colon and rectum'** OR **'cancer of rectum and colon'** OR **'cancer of the colon and rectum'** OR **'cancer of the colon and the rectum'** OR **'cancer of the rectum and colon'** OR **'cancer of the rectum and the colon'** OR **'colo-rectal cancer'** OR **'colo-rectal carcinogenesis'** OR **'colo-rectal malignancies'** OR **'colo-rectal malignancy'** OR **'colorectal cancerogenesis'** OR **'colorectal carcinogenesis'** OR **'colorectal malignancies'** OR **'colorectal malignancy'** OR **'malignancies of the colon and rectum'** OR **'malignancy of colon and rectum'** OR **'malignancy of the colon and rectum'** OR **'recto-colonic cancer'** OR **'rectocolonic cancer'** OR **'colorectal cancer'** |

| POSTN-EMBASE |
| --- |
| #1 AND #2 AND #3 AND [humans]/lim AND [english]/lim AND [<1966-2024]/py AND [article]/lim |
| [67](https://www.embase.com.ezproxy.medgrid.eu/)  **#5**  #1 AND #2 AND #3 AND [humans]/lim AND [english]/lim AND [<1966-2024]/py |
| [122](https://www.embase.com.ezproxy.medgrid.eu/)  **#4**  #1 AND #2 AND #3 |
| [137](https://www.embase.com.ezproxy.medgrid.eu/)  **#3**  'transcription factor runx2'/exp AND 'acute myeloid leukaemia 3 protein' OR 'acute myeloid leukemia 3 protein' OR 'aml3 protein' OR 'cbfa1 protein' OR 'core binding factor alpha 1 subunit' OR 'core binding factor alpha1' OR 'osf2' OR 'osteoblast specific factor 2' OR 'osteoblast specific transcription factor 2' OR 'periostin' OR 'postn protein' OR 'protein aml3' OR 'protein cbfa1' OR 'protein postn' OR 'protein runx2' OR 'runt related transcription factor 2' OR 'runx2 protein' OR 'transcription factor aml3' OR 'transcription factor cbfa1' OR 'transcription factor runx2' |
| [24,673](https://www.embase.com.ezproxy.medgrid.eu/)  **#2**  'prognosis'/exp OR 'survival'/exp OR 'prognosis' OR 'prognostic' OR 'survival' OR 'outcome' |
| [6,388,488](https://www.embase.com.ezproxy.medgrid.eu/)  **#1**  'colorectal cancer'/exp OR 'cancer of colon and rectum' OR 'cancer of rectum and colon' OR 'cancer of the colon and rectum' OR 'cancer of the colon and the rectum' OR 'cancer of the rectum and colon' OR 'cancer of the rectum and the colon' OR 'colo-rectal cancer' OR 'colo-rectal carcinogenesis' OR 'colo-rectal malignancies' OR 'colo-rectal malignancy' OR 'colorectal cancerogenesis' OR 'colorectal carcinogenesis' OR 'colorectal malignancies' OR 'colorectal malignancy' OR 'malignancies of the colon and rectum' OR 'malignancy of colon and rectum' OR 'malignancy of the colon and rectum' OR 'recto-colonic cancer' OR 'rectocolonic cancer' OR 'colorectal cancer' |

| TAGLN – EMBASE |
| --- |
|  |
| #1 AND #2 AND #3 AND [english]/lim AND [<1966-2024]/py AND [article]/lim |
| [57](https://www.embase.com.ezproxy.medgrid.eu/)  **#4**  #1 AND #2 AND #3 |
| [60](https://www.embase.com.ezproxy.medgrid.eu/)  **#3**  'transgelin'/exp OR 'protein sm 22' OR 'protein sm22' OR 'protein sm22 alpha' OR 'protein sm22alpha' OR 'protein tagln' OR 'protein ws3-10' OR 'sm 22 muscle protein' OR 'sm 22 protein' OR 'sm22 alpha protein' OR 'sm22 muscle protein' OR 'sm22 protein' OR 'sm22alpha protein' OR 'smooth muscle protein 22 alpha' OR 'smooth muscle protein 22alpha' OR 'tagln protein' OR 'ws3-10 protein' OR 'transgelin' |
| [2,750](https://www.embase.com.ezproxy.medgrid.eu/)  **#2**  'prognosis'/exp OR 'survival'/exp OR 'prognosis' OR 'prognostic' OR 'survival' OR 'outcome' |
| [6,388,488](https://www.embase.com.ezproxy.medgrid.eu/)  **#1**  'colorectal cancer'/exp OR 'cancer of colon and rectum' OR 'cancer of rectum and colon' OR 'cancer of the colon and rectum' OR 'cancer of the colon and the rectum' OR 'cancer of the rectum and colon' OR 'cancer of the rectum and the colon' OR 'colo-rectal cancer' OR 'colo-rectal carcinogenesis' OR 'colo-rectal malignancies' OR 'colo-rectal malignancy' OR 'colorectal cancerogenesis' OR 'colorectal carcinogenesis' OR 'colorectal malignancies' OR 'colorectal malignancy' OR 'malignancies of the colon and rectum' OR 'malignancy of colon and rectum' OR 'malignancy of the colon and rectum' OR 'recto-colonic cancer' OR 'rectocolonic cancer' OR 'colorectal cancer' |

| VIMENTIN – EMBASE |
| --- |
| #1 AND #2 AND #3 AND [article]/lim AND [english]/lim AND [humans]/lim AND [<1966-2024]/py AND ([adult]/lim OR [aged]/lim OR [very elderly]/lim) AND ([embase]/lim OR [medline]/lim) |
| [375](https://www.embase.com.ezproxy.medgrid.eu/)  **#5**  #1 AND #2 AND #3 AND [article]/lim AND [english]/lim AND [humans]/lim AND [<1966-2024]/py |
| [801](https://www.embase.com.ezproxy.medgrid.eu/)  **#4**  #1 AND #2 AND #3 |
| [1,379](https://www.embase.com.ezproxy.medgrid.eu/)  **#3**  **'vimentin'**/exp OR **'vimentin'** OR **'vim'** OR **'vim protein'** |
| [82,355](https://www.embase.com.ezproxy.medgrid.eu/)  **#2**  **'prognosis'**/exp OR **'survival'**/exp OR **'prognosis'** OR **'prognostic'** OR **'survival'** OR **'outcome'** |
| [6,386,321](https://www.embase.com.ezproxy.medgrid.eu/)  **#1**  **'colorectal cancer'**/exp OR **'cancer of colon and rectum'** OR **'cancer of rectum and colon'** OR **'cancer of the colon and rectum'** OR **'cancer of the colon and the rectum'** OR **'cancer of the rectum and colon'** OR **'cancer of the rectum and the colon'** OR **'colo-rectal cancer'** OR **'colo-rectal carcinogenesis'** OR **'colo-rectal malignancies'** OR **'colo-rectal malignancy'** OR **'colorectal cancerogenesis'** OR **'colorectal carcinogenesis'** OR **'colorectal malignancies'** OR **'colorectal malignancy'** OR **'malignancies of the colon and rectum'** OR **'malignancy of colon and rectum'** OR **'malignancy of the colon and rectum'** OR **'recto-colonic cancer'** OR **'rectocolonic cancer'** OR **'colorectal cancer'** |

| S100A4 – EMBASE |
| --- |

| #1 AND #2 AND #3 AND [article]/lim AND [english]/lim AND [<1966-2024]/py AND [humans]/lim  [72](https://www.embase.com.ezproxy.medgrid.eu/) |
| --- |
| **#5**  #1 AND #2 AND #3 AND [article]/lim AND [english]/lim AND [<1966-2024]/py |
| [74](https://www.embase.com.ezproxy.medgrid.eu/)  **#4**  #1 AND #2 AND #3 |
| [141](https://www.embase.com.ezproxy.medgrid.eu/)  **#3**  **'calvasculin'**/exp OR **'fibroblast specific protein 1'** OR **'fsp 1 protein'** OR **'fsp1 protein'** OR **'metastasin'** OR **'metastasis associated protein mts1'** OR **'metastasis related protein mts1'** OR **'mts1 protein'** OR **'placental calcium binding protein'** OR **'protein fsp 1'** OR **'protein fsp1'** OR **'protein mts1'** OR **'protein s 100a4'** OR **'protein s100a4'** OR **'s100 calcium binding protein a4'** OR **'s100 calcium-binding protein a4'** OR **'s100a4 protein'** OR **'calvasculin'** |
| [3,930](https://www.embase.com.ezproxy.medgrid.eu/)  **#2**  **'prognosis'**/exp OR **'survival'**/exp OR **'prognosis'** OR **'prognostic'** OR **'survival'** OR **'outcome'** |
| [6,386,321](https://www.embase.com.ezproxy.medgrid.eu/)  **#1**  **'colorectal cancer'**/exp OR **'cancer of colon and rectum'** OR **'cancer of rectum and colon'** OR **'cancer of the colon and rectum'** OR **'cancer of the colon and the rectum'** OR **'cancer of the rectum and colon'** OR **'cancer of the rectum and the colon'** OR **'colo-rectal cancer'** OR **'colo-rectal carcinogenesis'** OR **'colo-rectal malignancies'** OR **'colo-rectal malignancy'** OR **'colorectal cancerogenesis'** OR **'colorectal carcinogenesis'** OR **'colorectal malignancies'** OR **'colorectal malignancy'** OR **'malignancies of the colon and rectum'** OR **'malignancy of colon and rectum'** OR **'malignancy of the colon and rectum'** OR **'recto-colonic cancer'** OR **'rectocolonic cancer'** OR **'colorectal cancer'** |

| GENERAL SEARCH- EMBASE |
| --- |
| #1 AND #2 AND #3 AND [article]/lim AND ([adult]/lim OR [aged]/lim OR [very elderly]/lim) AND [humans]/lim AND [english]/lim AND [<1966-2024]/py |
| [158](https://www.embase.com.ezproxy.medgrid.eu/)  **#4**  #1 AND #2 AND #3 |
| [998](https://www.embase.com.ezproxy.medgrid.eu/)  **#3**  (**'cancer associated fibroblast'**/exp OR **'cancer associated fibroblast'**) AND (**'cancer associated fibroblasts'**/exp OR **'cancer associated fibroblasts'**) OR **'cancer-associated fibroblast'**/exp OR **'cancer-associated fibroblast'** OR **'cancer-associated fibroblasts'**/exp OR **'cancer-associated fibroblasts'** OR **'tumor associated fibroblast'**/exp OR **'tumor associated fibroblast'** OR **'tumor associated fibroblasts'**/exp OR **'tumor associated fibroblasts'** OR **'tumor-associated fibroblast'**/exp OR **'tumor-associated fibroblast'** OR **'tumor-associated fibroblasts'**/exp OR **'tumor-associated fibroblasts'** OR **'cancer associated fibroblast'**/exp OR **'cancer associated fibroblast'** |
| [14,370](https://www.embase.com.ezproxy.medgrid.eu/)  **#2**  **'prognosis'**/exp OR **'survival'**/exp OR **'prognosis'** OR **'prognostic'** OR **'survival'** OR **'outcome'** |
| [6,388,488](https://www.embase.com.ezproxy.medgrid.eu/)  **#1**  **'colorectal cancer'**/exp OR **'cancer of colon and rectum'** OR **'cancer of rectum and colon'** OR **'cancer of the colon and rectum'** OR **'cancer of the colon and the rectum'** OR **'cancer of the rectum and colon'** OR **'cancer of the rectum and the colon'** OR **'colo-rectal cancer'** OR **'colo-rectal carcinogenesis'** OR **'colo-rectal malignancies'** OR **'colo-rectal malignancy'** OR **'colorectal cancerogenesis'** OR **'colorectal carcinogenesis'** OR **'colorectal malignancies'** OR **'colorectal malignancy'** OR **'malignancies of the colon and rectum'** OR **'malignancy of colon and rectum'** OR **'malignancy of the colon and rectum'** OR **'recto-colonic cancer'** OR **'rectocolonic cancer'** OR **'colorectal cancer'** |

| COL11A1- EMBASE  #1 AND #2 AND #3 AND [article]/lim AND [english]/lim AND [<1966-2024]/py  [15](https://www.embase.com.ezproxy.medgrid.eu/) |
| --- |
| **#4**  #1 AND #2 AND #3  [29](https://www.embase.com.ezproxy.medgrid.eu/) |
| **#3**  **'collagen type xi alpha 1 chain'**/exp OR **'col11a1'** OR **'collagen type xi alpha 1 chain'** OR **'collagen type 11 alpha 1 chain'** OR **'collagen xi, alpha-1 polypeptide'** OR **'coll6 protein'** OR **'dfna37'** OR **'collagen, type xi, alpha 1, human'** OR **'stl2'**  [1,073](https://www.embase.com.ezproxy.medgrid.eu/) |
| **#2**  **'prognosis'**/exp OR **'survival'**/exp OR **'prognosis'** OR **'prognostic'** OR **'survival'** OR **'outcome'**  [6,386,321](https://www.embase.com.ezproxy.medgrid.eu/) |
| **#1**  **'colorectal cancer'**/exp OR **'cancer of colon and rectum'** OR **'cancer of rectum and colon'** OR **'cancer of the colon and rectum'** OR **'cancer of the colon and the rectum'** OR **'cancer of the rectum and colon'** OR **'cancer of the rectum and the colon'** OR **'colo-rectal cancer'** OR **'colo-rectal carcinogenesis'** OR **'colo-rectal malignancies'** OR **'colo-rectal malignancy'** OR **'colorectal cancerogenesis'** OR **'colorectal carcinogenesis'** OR **'colorectal malignancies'** OR **'colorectal malignancy'** OR **'malignancies of the colon and rectum'** OR **'malignancy of colon and rectum'** OR **'malignancy of the colon and rectum'** OR **'recto-colonic cancer'** OR **'rectocolonic cancer'** OR **'colorectal cancer'**  [468,202](https://www.embase.com.ezproxy.medgrid.eu/) |

| COL1A1 – EMBASE |
| --- |
| #1 AND #2 AND #3 AND [article]/lim AND [english]/lim AND ([embase]/lim OR [medline]/lim) AND [<1966-2024]/py |
| [38](https://www.embase.com.ezproxy.medgrid.eu/) |
| **#4**  #1 AND #2 AND #3  [49](https://www.embase.com.ezproxy.medgrid.eu/) |
| **#3**  **'collagen type i alpha 1 chain'**/exp OR **'alpha-1 type 1 collagen'** OR **'alpha-1 type i collagen'** OR **'col1a1 protein'** OR **'collagen alpha 1 (i)'** OR **'collagen alpha-1 (i) chain'** OR **'collagen type 1 alpha1 chain'** OR **'collagen type 1, alpha 1 chain'** OR **'collagen type i, alpha 1 chain'** OR **'protein col1a1'** OR **'type i collagen alpha 1'** OR **'collagen type i alpha 1 chain'**  [4,870](https://www.embase.com.ezproxy.medgrid.eu/) |
| **#2**  **'prognosis'**/exp OR **'survival'**/exp OR **'prognosis'** OR **'prognostic'** OR **'survival'** OR **'outcome'**  [6,386,321](https://www.embase.com.ezproxy.medgrid.eu/) |
| **#1**  **'colorectal cancer'**/exp OR **'cancer of colon and rectum'** OR **'cancer of rectum and colon'** OR **'cancer of the colon and rectum'** OR **'cancer of the colon and the rectum'** OR **'cancer of the rectum and colon'** OR **'cancer of the rectum and the colon'** OR **'colo-rectal cancer'** OR **'colo-rectal carcinogenesis'** OR **'colo-rectal malignancies'** OR **'colo-rectal malignancy'** OR **'colorectal cancerogenesis'** OR **'colorectal carcinogenesis'** OR **'colorectal malignancies'** OR **'colorectal malignancy'** OR **'malignancies of the colon and rectum'** OR **'malignancy of colon and rectum'** OR **'malignancy of the colon and rectum'** OR **'recto-colonic cancer'** OR **'rectocolonic cancer'** OR **'colorectal cancer'** |

| DECORIN – EMBASE  #1 AND #2 AND #3 AND [article]/lim AND [humans]/lim AND [english]/lim AND [<1966-2024]/py  [25](https://www.embase.com.ezproxy.medgrid.eu/) |
| --- |
| **#4**  #1 AND #2 AND #3  [43](https://www.embase.com.ezproxy.medgrid.eu/) |
| **#3**  **'decorin'**/exp OR **'decorin'** OR **'dcn'** OR **'dspg-ii'** OR **'bone proteoglycan ii'**  [9,497](https://www.embase.com.ezproxy.medgrid.eu/) |
| **#2**  **'prognosis'**/exp OR **'survival'**/exp OR **'prognosis'** OR **'prognostic'** OR **'survival'** OR **'outcome'**  [6,386,321](https://www.embase.com.ezproxy.medgrid.eu/) |
| **#1**  **'colorectal cancer'**/exp OR **'cancer of colon and rectum'** OR **'cancer of rectum and colon'** OR **'cancer of the colon and rectum'** OR **'cancer of the colon and the rectum'** OR **'cancer of the rectum and colon'** OR **'cancer of the rectum and the colon'** OR **'colo-rectal cancer'** OR **'colo-rectal carcinogenesis'** OR **'colo-rectal malignancies'** OR **'colo-rectal malignancy'** OR **'colorectal cancerogenesis'** OR **'colorectal carcinogenesis'** OR **'colorectal malignancies'** OR **'colorectal malignancy'** OR **'malignancies of the colon and rectum'** OR **'malignancy of colon and rectum'** OR **'malignancy of the colon and rectum'** OR **'recto-colonic cancer'** OR **'rectocolonic cancer'** OR **'colorectal cancer'** |
