# Supplementary material for: Cancer-associated fibroblasts and their prognostic role in colorectal cancer: review and meta-analysis
Source: Front Oncol. 2025 Dec 10;15:1635055. doi: 10.3389/fonc.2025.1635055 (PMC12727558; doi:10.3389/fonc.2025.1635055)
Supplement: Supplementary file 5 [file Table3.doc]

Table 3 – Characteristics of studies included

| **Study** | **Country** | **Patients (n)** | **Age (years)** | **Follow-up (months)** | **Adjustment** | **Proteins assayed** | **Outcome** | **Quality score** |
| --- | --- | --- | --- | --- | --- | --- | --- | --- |
| Wang 2017 (75) | China | 102 | NS | 55 | NS | VIM | OS, DFS | 7/9 |
| Toiyama 2013(77) | Japan | 208 | *68 | 50 | NS | VIM | OS | 7/9 |
| Lau 2018(91) | Australia | 37 | ^58.8 | 39 | NS | VIM | OS, RFS | 6/9 |
| Secinti 2022 (92) | Turkey | 84 | ^62.8 | NS | NS | VIM | OS | 6/9 |
| Liu 2017 (36) | China | 203 | NS | NS | NS | VIM | CSS, DFS | 7/9 |
| Xiao 2015 (76) | China | 105 | ^52 | NS | NS | VIM | OS | 7/9 |
| Zasada 2022 (35) | Poland | 97 | 68 | 60 | NS | VIM | OS | 7/9 |
| Yun 2014 (73) | Korea | 409 | ^59 | NS | NS | VIM, α-SMA | OS, DFS | 7/9 |
| Ikuta 2018 (60) | Japan | 94 | *66 | 59.9 | NS | α-SMA | RFS | 6/9 |
| Hashimoto 2021(59) | Japan | 286 | NS | NS | NS | α-SMA, TNC | OS, DFS | 7/9 |
| Murakami 2017 (90) | Japan | 139 | NS | NS | NS | TNC | OS | 6/9 |
| Yang 2020 (105) | Japan | 100 | NS | 112 | NS | TNC | DFS, OS | 6/9 |
| Yang 2018 (106) | Japan | 100 | NS | 112 | NS | TNC | OS, DFS | 6/9 |
| Ito 2023 (33) | Japan | 259 | *69 | 60 | NS | TNC | DFS, OS | 5/9 |
| Fujino 2018 (23) | Japan | 21 | NS | 45.36 | NS | PDGFR‐β | OS, DFS | 6/9 |
| **Study** | **Country** | **Patients (n)** | **Age (years)** | **Follow-up (months)** | **Adjustment** | **Proteins assayed** | **Outcome** | **Quality score** |
| Mezheyeuski 2016 (83) | Sweden, Denmark, Norway, Belarus | 311 | NS | NS | NS | PDGFR-β | OS | 5/9 |
| Kazakova 2023 (88) | Russia | 118 | ^66,3 | NS | Age, pTNM, LVI | S100A4 | OS | 5/9 |
| Niu 2015 (89) | China | 131 | *57.9 | 56 | NS | S100A4 | OS | 6/9 |
| Kho 2012 (41) | Australia | 451 | NS | NS | Age, Grade, pTNM, Treatment | S100A4 | OS | 7/9 |
| Boye 2016 (61) | Norway | 783 | *73/63 | 116.4/ 91.2 | NS | S100A4 | RFS | 8/9 |
| Kang 2011 (40) | Korea | 526 | NS | 40.1 | NS | S100A4 | OS, RFS | 6/9 |
| Huang 2011(43) | China | 112 | NS | NS | NS | S100A4 | OS, RFS | 6/9 |
| Sugai 2017 (42) | Japan | 106 | NS | 66 | NS | S100A4 | OS | 6/9 |
| Boye 2010 (126) | Norway | 237 | ^73 | NS | NS | S100A4 | DFS, OS | 8/9 |
| Kwak 2010 (127) | Korea | 127 | ^59,3 | 58,7 | NS | S100A4 | OS | 6/9 |
| Zhang 2010 (80) | China | 120 | ^62.2 | 53.3 | NS | TAGLN2 | OS | 6/9 |
| Xu 2016 (28) | China | 192 | NS | NS | NS | TAGLN2 | OS, DFS | 7/9 |
| **Study** | **Country** | **Patients (n)** | **Age (years)** | **Follow-up (months)** | **Adjustment** | **Proteins assayed** | **Outcome** | **Quality score** |
| Zhao 2009 (27) | China | 126 | NS | NS | NS | TAGLN2 | OS | 7/9 |
| Yang 2017 (108) | China | 179 | ^59,5 | 60 | NS | MMP9 | PFS, OS | 6/9 |
| Ogata 2005 (66) | Japan | 307 | ^61,9/70,9 | 64/87 | NS | MMP9 | DFS | 6/9 |
| Langers 2012 (109) | Netherlands | 198 | NS | 60 | NS | MMP9 | OS | 7/9 |
| Jensen 2010 (34) | Denmark | 340 | NS | 73.2 | NS | MMP9 | RFS, OS | 6/9 |
| Chu 2011 (31) | China | 192 | NS | 56 | Gender, Age, Grade, pTNM, LVI | MMP9 | DFS, OS | 7/9 |
| Buhmeida 2009 (65) | Turkey | 202 | NS | >240 | Gender, Age, pTNM, Site | MMP9 | DFS | 7/9 |
| Wang 2019 (111) | China | 443 | NS | 60 | NS | MMP9 | OS | 7/9 |
| Bendardaf 2009 (112) | Turkey | 359 | NS | 240 | NS | MMP9 | DFS | 7/9 |
| Zhou 2011 (26) | China | 141 | *59 | 59 | NS | MMP2 | DFS, OS | 7/9 |
| Šundov 2008 (95) | Croatia | 152 | ^62 | NS | NS | MMP2 | OS | 6/9 |
| Dong 2011 (96) | China | 172 | *56.5 | NS | NS | MMP2 | OS | 7/9 |
| **Study** | **Country** | **Patients (n)** | **Age (years)** | **Follow-up (months)** | **Adjustment** | **Proteins assayed** | **Outcome** | **Quality score** |
| Hilska 2007 (97) | Turkey | 351 | NS | NS | NS | MMP2 | OS | 6/9 |
| Deng 2017 (25) | China | 463 | NS | 60 | NS | MMP2 | OS | 6/9 |
| Unsal 2008 (93) | Turkey | 60 | NS | 29.45 | NS | MMP9, MMP2 | OS, DFS | 7/9 |
| Salem 2016 (64) | Saudi Arabia | 127 | NS | NS | Age, grade, pN | MMP9, MMP2 | OS, DFS | 6/9 |
| Peltonen 2020 (94) | Finland | 111 | *62.8 | 182,4 | NS | MMP9, MMP2 | OS, DFS | 7/9 |
| Langer 2008 (98) | Germany | 215 | NS | NS | NS | MMP9, MMP2 | OS | 7/9 |
| Langenskiöld 2013 (113) | Sweden | 136 | ^73/ 81 | 65 | NS | MMP9, MMP2 | CSS | 8/9 |
| Araújo 2015 (110) | Brazil | 180 | ^63 | 60 | NS | MMP9, MMP2 | OS | 6/9 |
| Fukasawa 2009 (44) | Japan | 165 | ^61.8 | 61 | NS | CXCL12 | RFS, OS | 7/9 |
| D’Alterio 2012 (103) | Italy | 68 | NS | 64 | NS | CXCL12 | RFS, CSS | 7/9 |
| Stanisavljević 2015 (29) | Norway | 502 | ^61.9 | NS | Age, Gender, pTNM, Treatment | CXCL12 | DFS | 7/9 |
| Yoshitake 2008 (104) | Japan | 60 | ^63.8 | NS | NS | CXCL12 | OS | 6/9 |
| **Study** | **Country** | **Patients (n)** | **Age (years)** | **Follow-up (months)** | **Adjustment** | **Proteins assayed** | **Outcome** | **Quality score** |
| Zengin 2021(46) | Turkey | 260 | *69 | 60 | NS | CXCL12 | RFS, OS | 7/9 |
| Okikawa 2021 (45) | Japan | 98 | NS | 60 | NS | CXCL12 | DFS, OS | 6/9 |
| Kim 2021 (38) | South Korea | 121 | NS | 45 | NS | CXCL12, FAP | RFS, OS | 6/9 |
| Herrera 2020 (114) | Sweden | 520 | NS | 100 | Age, pTNM, Treatment, Gender, Site | FAP | OS | 7/9 |
| Coto 2020 (39) | Switzerland | 92 | NS | 72 | NS | FAP | OS | 5/9 |
| Wikberg 2013 (102) | Sweden | 449 | NS | NS | MSI status | FAP | CSS | 6/9 |
| Brown 2021 (125) | Australia | 110 | *62.88 | 66/45 | NS | FAP, POSTN | DFS  OS | 7/9 |
| Oh 2017(24) | Korea | 1125 | *62 | 69.8 | NS | POSTN | OS, PFS | 6/9 |
| Li 2015 (86) | China | 115 | NS | 61 | NS | POSTN | OS | 6/9 |
| Thongchot 2020 (87) | Thailand | 410 | ^ 64 | NS | NS | POSTN | OS | 6/9 |
| Xu 2015 (32) | China | 1025 | NS | 60 | pTNM, Treatment | POSTN | DFS, DSS | 7/9 |
| Cai 2019 (48) | China | 164 | ^62 | 60 | NS | PDPN | OS | 6/9 |
| Algars 2011 (107) | Finland | 145 | *72,8 | 66,2 | NS | PDPN | DSS | 7/9 |
| **Study** | **Country** | **Patients (n)** | **Age (years)** | **Follow-up (months)** | **Adjustment** | **Proteins assayed** | **Outcome** | **Quality score** |
| Yamanashi 2009(47) | Japan | 120 | *60 | 62,4 | pTNM, LVI | PDPN | RFS, DFS | 7/9 |
| Ye 2019 (30) | China | 1008 | NS | 60 | NS | CD 163 | DFS, OS | 7/9 |
| Xu 2021 (115) | China | 1021 | NS | 58 | NS | CD 163 | DFS, OS | 6/9 |
| Wang 2023 (116) | China | 255 | NS | 150 | NS | CD 163 | OS | 6/9 |
| Wen 2020 (117) | Sweeden | 219 | NS | 60 | NS | CD 163 | DFS, OS | 8/9 |
| Wei 2019 (118) | China | 81 | NS | >60 | NS | CD 163 | RFS, OS | 6/9 |
| Takasu 2021(119) | Japan | 71 | ^66,9 | 51,9 | NS | CD 163 | OS | 6/9 |
| Shin 2021 (120) | Korea | 148 | ^67.2 | >60 | NS | CD 163 | DFS, OS | 7/9 |
| Shabo 2014 (69) | Sweeden | 75 | NS | 120 | NS | CD 163 | OS | 6/9 |
| Ozaki 2023 (121) | Japan | 205 | *66 | 60 | NS | CD 163 | RFS | 7/9 |
| Kitagawa 2022 (122) | Japan | 275 | NS | 73 | NS | CD 163 | RFS, OS | 6/9 |
| Edin 2012 (123) | Sweden | 422 | NS | >60 | NS | CD 163 | CSS | 7/9 |
| Blom 2023 (37) | Sweden | 537 | *71 | NS | NS | CD 163 | OS | 7/9 |
| Xue 2021 (124) | China | 209 | NS | >50 | NS | CD 163 | DFS, OS | 6/9 |
| Akter 2022 (128) | Korea | 399 | NS | 42.1 | NS | CD163 | RFS, OS | 6/9 |
| Ke 2023 (129) | China | 45 | NS | NS | NS | CD163 | OS | 5/9 |
| **Study** | **Country** | **Patients (n)** | **Age (years)** | **Follow-up (months)** | **Adjustment** | **Proteins assayed** | **Outcome** | **Quality score** |
| Kanno 2020 (130) | Japan | 117 | *70 | >60 | NS | CD163 | OS, DFS | 6/9 |
| Liu 2021 (99) | China | 191 | NS | NS | NS | CD163 | OS, DFS | 6/9 |
| Ledys 2018 (100) | France | 114 | *63 | 34.8 | NS | CD163 | OS, PFS | 7/9 |
| Cavalleri 2022 (101) | Italy | 165 | NS | 57.48 | NS | CD163 | DFS | 7/9 |

Table 1 - Characteristics of the included studies; α-SMA = alpha smooth muscle actin; CD = cluster of differentiation; CSS = cancer specific survival; CXCL12 = C-X-C motif chemokine ligand 12; DFS = disease free survival; FAP = fibroblast activation protein α; LVI = lymphovascular invasion; MMP = matrix metalloproteinase; No = number; NS = Not specified; MSI = microsatellite instability; OS = overall survival; PDGFR - β = platelet derived growth factor subunit B; PDPN = podoplanin; POSTN = periostin; pTNM = pathologic tumor node metastasis; ; RFS = relapse free survival; S100A4 = calcium binding protein A4; TAGLN 2 = transgelin2; TNC = tenascin C; Vim = vimentin; *= median age; ^= mean age
