# Supplementary material for: Cancer-associated fibroblasts and their prognostic role in colorectal cancer: review and meta-analysis
Source: Front Oncol. 2025 Dec 10;15:1635055. doi: 10.3389/fonc.2025.1635055 (PMC12727558; doi:10.3389/fonc.2025.1635055)
Supplement: Supplementary file 6 [file Table4.doc]

Table 4. Summary of studies evaluating MMP2 expression in colorectal cancer and associated clinical outcomes.

| **Study** | **Patients** | **Stage** | **Compartment** | **Type of tissue specimen** | **Determination of CAF expression in samples** | **Cut-off** | **Outcomes (HR [95% CI], *p)*** | **Analysis** |
| --- | --- | --- | --- | --- | --- | --- | --- | --- |
| Deng 2017 (25) | 463 | I-IV | NS | PT | score^a^ | ≥ 4 | OS: 0.63 (0.46 - 0.82), *p* = 0.004 | MV |
| Unsal 2008 (93) | 60 | II-III | S | PT | score^b^ | ≥ 2 | DFS: 0.93 (0.28 - 3.06), NS  OS: 1.32 (0.31 - 5.26), NS | UV |
| Salem 2016 (64) | 127 | II-IV | C | PT | qualitative | ≥ 1 | DFS: 3.85 (1.48 - 10.02), *p* = 0.006 | UV |
| Peltonen 2020 a (94) | 111 | IV | C | PT | qualitative | ≥ 2 | DFS: 1.03 (0.49 - 2.18), *p* = 0.931  OS: 0.79 (0.37 - 1.69), *p* = 0.549 | MV |
| Peltonen 2020 b (94) | 111 | IV | C | CRLM | qualitative | ≥ 2 | DFS: 0.77 (0.48 - 1.23), *p* = 0.286  OS: 0.74 (0.43 - 1.28), *p* = 0.281 | UV |
| Sundov 2008 (95) | 152 | II | C | PT | quantitative | ≥10% | OS: 3.64 (1.79 - 7.36 ), *p* <0.001 | MV |
| Dong 2011 (96) | 172 | I-IV | C | PT | score^c^ | ≥ 6 | OS: 3.76 (1.43 - 9.88), *p* = 0.007 | MV |
| Hilska 2007 a (135) | 351 | I-IV | C | PT | score^d^ | 0.65 | CSS: 1.33 (0.68 - 2.63), *p* = 0.41 | MV |
| Hilska 2007 b (135) | 351 | I-IV | C | PT | score^d^ | 0.65 | CSS: 1.22 (0.82 - 2.93), *p* = 0.17 | MV |
| Langers 2008 (98) | 215 | I-IV | NA | PT | ELISA | ≥ 18.5 | OS: 1.42 (0.98 - 2.04), *p* = 0.062 | MV |
| Langers 2012 (110) | 198 | I-IV | NA | PT | ELISA | ≥ 8.7 | OS: 1.72 (1.15 – 2.58), *p* = 0.766 | MV |
| Araújo 2015 (112) | 180 | I-IV | C | PT | quantitative | ≥ 2 | OS: 1.36 (0.96 - 1.77), NS | MV |
| Langenskiöld 2013 a (115) | 103 | I-III | S | PT | ELISA | NS* | CSS: 1.01 (1.00 – 1.02), *p* = 0.060 | MV |
| Langenskiöld 2013 b (115) | 33 | I-III | S | PT | ELISA | NS* | CSS: 1.01 (0.99 – 1.02), *p* = 0.476 | MV |
| Zhou 2011 (116) | 141 | II-III | N | PT | NS | NS | DFS: 0.23 (0.10 – 0.54), *p* = 0.001  OS: 0.39 (0.19 – 0.84), *p* = 0.015 | MV |

MMP = matrix metalloproteinase; OS = Overall survival; DFS = disease free survival; CSS = cancer specific survival; *p*<0.05 = statistical significance;; HR = Hazard ratio; CI = Confidence Interval; C = cytoplasm; S = stromal; N = nucleus; M= membrane; NS = Not specified; NA= Not applicable; PT = primary tumor; CRLM = ColoRectal cancer Liver Metastasis; Hilska a = intratumoral expression; Hilska b= stromal expression; MV = multivariate analysis; UV = univariate analysis; quantitative = based on area of stained cells; qualitative = based on intensity of stained cells; Peltonen a= biomarker in primary tumor

Peltonen b = biomarker in CRLM; Langenskiöld a = patients with colon cancer; Langenskiöld b = patients with rectal cancer ; * cut-off selected based on the respective receiver operating characteristics (ROC) curve;

score a = score (IRS) of immunostaining, score b =intensity x percentage of stained cells; score c = intensity + percentage of stained cells, score d = [percentage of cells with intesity “ 1+”]+ [(percentage of cells with intesity “ 2+”)x2)]+ [(percentage of cells with intesity “ 3+”)x3];

Table 5 - Summary of the characteristics of studies included researching FAP expression

| **Study** | **Patients** | **Stage** | **Compartment** | **Type of tissue specimen** | **Determination of CAF expression in samples** | **Cut-off** | **Outcomes (HR [95% CI], *p)*** | **Analysis** |
| --- | --- | --- | --- | --- | --- | --- | --- | --- |
| Wikberg 2013 (102) | 449 | I-IV | S | PT | qualitative | +++ | CSS: 1.72 (1.07-2.76), *p* = 0.026 | MV |
| Herrera 2020 a (117) | 253 | I-IV | S | PT | qualitative | ≥1 | OS: 0.54 (0.33-0.87), *p* =0.001 | MV |
| Herrera 2020 b (117) | 267 | II-III | S | PT | qualitative | ≥1 | OS: 0.63 (0.41-0.95), *p* = 0.029 | MV |
| Coto 2020 (38) | 92 | I-IV | S, N | PT | score | >10% | OS: 3.0 (1.1 – 8.3), *p* = 0.04 | MV |
| Kim 2021 (37) | 121 | II-III | M | PT | qualitative | ≥2 | RFS: 1.12 (0.30 - 4.08), *p* = 0.868  OS: 0.44 (0.099 - 1.97), *p* = 0.284 | MV |
| Brown 2021 (129) a | 110 | IV | S | PT+CRLM | qualitative | ≥3 | DFS: 0.91 (0.49-1.72), *p* = 0.781  OS: 1.11 (0.53-2.31), *p* = 0.789 | UV |
| Brown 2021 (129) b | 110 | IV | S | PT+CRLM | qualitative | ≥2 | DFS: 1.19 (0.55-2.60), *p* = 0.662  OS: 1.66 (0.70-3.94), *p* = 0.253 | UV |

FAP = fibroblast activation proteinα; OS = Overall survival; DFS = disease free survival; RFS = relapse free survival; CSS = cancer specific survival; *p*<0.05 = statistical significance; HR = Hazard ratio; S = stromal; N = nucleus; M= membrane; NS = Not specified; PT = primary tumor; CRLM = ColoRectal cancer Liver Metastasis; MV = multivariate analysis; UV = univariate analysis Herrera a = a population-based Colorectal cancer cohort; Herrera b = stage II/III CRC patients from the “Nordic adjuvant randomized clinical trial”; Brown a = biomarker intratumor expression; Brown b = biomarker stromal expression;

Score = proportion of positive staining in stromal cells in 10% increments and the maximal staining intensity

Table 6 - Summary of the characteristics of studies included researching α-SMA expression

| **Study** | **Patients** | **Stage** | **Compartment** | **Type of tissue specimen** | **Determination of CAF expression in samples** | **Cut-off** | **Outcomes (HR [95% CI], *p)*** | **Analysis** |
| --- | --- | --- | --- | --- | --- | --- | --- | --- |
| Ikuta 2018 (59) | 94 | III-IV | I | PT | quantitative | ≥23.8 | OS: 1.18 (0.56 - 3.55), *p* = 0.463 | MV |
| Hashimoto 2021 a (58) | 148 | II-III | I | PT | score | ≥2 | OS: 0.63 (0.19 - 3.90), *p* = 0.553  DFS: 0.68 (0.25 - 2.82), *p* = 0.546 | UV |
| Hashimoto 2021 b (58) | 138 | II-III | I | PT | score | ≥2 | OS: 1.58 (0.33 - 28.26), *p* = 0.630  DFS: 1.42 (0.44-8.74 ), *p* = 0.607 | UV |

α-SMA = alpha smooth muscle actin; OS = Overall survival; DFS = disease free survival; *p*<0.05 = statistical significance;; HR = Hazard ratio; CI = Confidence Interval; I = interstitial; PT = primary tumor; MV = multivariate analysis; UV = univariate analysis Hashimoto a = test cohort; Hashimoto b = validation cohort;

score: = combination of intensity and extent of staining

Table 7 - Summary of the characteristics of studies included researching TAGLN 2 expression

| **Study** | **Patients** | **Stage** | **Compartment** | **Type of tissue specimen** | **Determination of CAF expression in samples** | **Cut-off** | **Outcomes (HR [95% CI], *p)*** | **Analysis** |
| --- | --- | --- | --- | --- | --- | --- | --- | --- |
| Zhao 2009 (27) | 126 | I-IV | C | PT | quantitative | ≥10% | OS: 2.34 (1.12–4.89 ), *p* = 0.02 | MV |
| Zhang 2010 (81) | 120 | I-IV | C | PT | score^a^ | ≥5 | OS: 1.639 (1.018–2.638), *p* = 0.042 | MV |
| Xu 2016 (28) | 192 | III | C | PT | score^b^ | ≥30% | DFS: 1.85 (1.07-3.20 ), *p* = 0.028  OS: 1.95(0.98-3.87 ), *p* = 0.059 | MV |

TAGLN 2 = transgelin 2 ; OS = Overall survival; DFS = disease free survival; *p*<0.05 = statistical significance;; HR = Hazard ratio; CI = Confidence Interval; C = cytoplasm; PT = primary tumor; MV = multivariate analysis;

score a = intensity + area of stained cells; score b = Immunoreactivity: Negative = no or weak staining, or staining in less than 30% of all cells regardless of the intensity. Positive expression = moderate to strong staining in at least 30% of all cells

Table 8 - Summary of the characteristics of studies included researching PDGFR beta expression

| **Study** | **Patients** | **Stage** | **Compartment** | **Type of tissue specimen** | **Determination of CAF expression in samples** | **Cut-off** | **Outcomes (HR [95% CI], *p)*** | **Analysis** |
| --- | --- | --- | --- | --- | --- | --- | --- | --- |
| Fujino 2018 (23) | 21 | I-IV | C,M | PT | quantitative | >50% | OS: 1.81(0.95-3.59), *p* = 0.068  DFS: 1.85 (1.02-3.44), *p* = 0.040 | UV  MV |
| Mezheyeuski 2016 (84) | 311 | IV | P | PT | qualitative | ≥2 | OS: 1.82 (1.00-3.04), *p* = 0.023 | MV |

PDGFR - β = platelet derived growth factor subunit B; OS = Overall survival; DFS = disease free survival; *p*<0.05 = statistical significance;; HR = Hazard ratio;

CI = Confidence Interval; C = cytoplasm; C = cytoplasm; S = stromal; N = nucleus; M= membrane; NS = Not specified; PT = primary tumor; MV = multivariate analysis; UV = univariate analysis; quantitative = based on area of stained cells; qualitative = based on intensity of stained cells;

Table 9 - Summary of the characteristics of studies included researching POSTN expression

| **Study** | **Patients** | **Stage** | **Compartment** | **Type of tissue specimen** | **Determination of CAF expression in samples** | **Cut-off** | **Outcomes (HR [95% CI], *p)*** | **Analysis** |
| --- | --- | --- | --- | --- | --- | --- | --- | --- |
| Oh 2017 (24) | 1125 | I-IV | S | PT | qualitative | 3 | OS: 1.50(1.13-2.00), *p* = 0.006  PFS: 1.40 (1.08-1.83), 0.012 | MV |
| Li 2015 (86) | 115 | I-IV | C | PT | score^a^ | 4 | OS: 2.02 (0.959 - 4.266 ), *p* = 0.044 | MV |
| Thongchot 2020 (87) | 410 | I-IV | C | PT | score^b^ | 4 | OS: 2.57 (1.21-5.457), *p* = 0.013 | MV |
| Xu 2015 a (32) | 682 | I-III | S | PT | score^c^ | 6 | DFS: 5.14 (3.21-8.23), *p* < 0.001  DSS: 4,14 (2.12-8.11), *p* < 0.001 | MV |
| Xu 2015 b (32) | 682 | I-III | S | PT | score^c^ | 9 | DFS: 11.85 (6.61-21.23), *p* < 0.001  DSS: 9.18 (4.15-20.34), *p* < 0.001 | MV |
| Xu 2015 c (32) | 682 | I-III | S | PT | score^c^ | 6 | DFS: 0.63 (0.38-1.04), *p* = 0.069  DSS: 1.06 (0.55-2.04), *p* = 0.868 | MV |
| Xu 2015 d (32) | 682 | I-III | S | PT | score^c^ | 9 | DFS: 0.83 (0.46–1.50), *p* = 0.534  DSS: 1.16 (0.51–2.62), *p* = 0.720 | MV |
| Xu 2015 e (32) | 343 | I-III | S | PT | score^c^ | 6 | DSS: 2.71 (1.43-5.14), *p* = 0.002 | MV |
| Xu 2015 f (32) | 343 | I-III | S | PT | score^c^ | 9 | DSS: 5.69 (2.93-11.05), *p* < 0.001 | MV |
| Xu 2015 g (32) | 343 | I-III | S | PT | score^c^ | 6 | DSS: 1.22 (0.64-2.32), *p* = 0.541 | MV |
| Xu 2015 h (32) | 343 | I-III | S | PT | score^c^ | 9 | DSS: 3.76 (2.93-11.05), *p* = 0.005 | MV |
| Brown 2021 a (129) | 110 | IV | S | PT +CRLM | qualitative | 1 | DFS: 1.04 (0.57-1.91), *p* = 0.891  OS: 1.26 (0.56-2.84), *p* = 0.572 | UV |
| Brown 2021 b(129) | 110 | IV | S | PT +CRLM | qualitative | 3 | DFS: 3.57 (1.23-10.38), *p* = 0.019  OS: 2.09 (0.74-5.85), *p* = 0.162 | MV |

POSTN= periostin; OS = Overall survival; DFS = disease free survival; *p*<0.05 = statistical significance;; HR = Hazard ratio; CI = Confidence Interval; C = cytoplasm; C = cytoplasm; S = stromal; N = nucleus; M= membrane; NS = Not specified; PT = primary tumor; CRLM = ColoRectal cancer Liver Metastasis; MV = multivariate analysis; UV = univariate analysis; quantitative = based on area of stained cells; qualitative = based on intensity of stained cells; score = combination of intensity and percentage of stained cells Brown a = POSTN intratumoral expression; Brown b = POSTN stromal expression; Xu a= Shanghai Cohort Stromal medium, Xu b= Shanghai Cohort Stromal HIGH, Xu c= Shanghai Cohort intratumoral medium, Xu d = Shanghai Cohort intratumoral high, Xu e = Guangzhou Cohort Stromal medium, Xu f = Guangzhou Cohort Stromal high, Xu g = Guangzhou Cohort intratumoral medium, Xu h = Guangzhou Cohort intratumoral high

score a= sum of immunostaining intensity score and immunoreactivity; score b = intensity x extent of staining

Table 10 - Summary of the characteristics of studies included researching S100A4 expression

| **Study** | **Patients** | **Stage** | **Compartment** | **Type of tissue specimen** | **Determination of CAF expression in samples** | **Cut-off** | **Outcomes (HR [95% CI], *p)*** | **Analysis** |
| --- | --- | --- | --- | --- | --- | --- | --- | --- |
| Kazakova 2023 (88) | 118 | I-III | S | PT | quantitative | 11.68 | OS: 1.10 (0.50-2.44), *p* = 0.798 | MV |
| Niu 2014 (89) | 131 | I-IV | C, N | PT | score^a^ | 3 | OS: 1.20 (0.66–2.20), *p* = 0.543 | MV |
| Kho 2012 a (40) | 404 | III | C | PT | quantitative | 70% | OS: 1.6 (1.1-2.2), *p* = 0.008 | MV |
| Kho 2012 b (40) | 404 | III | N | PT | quantitative | 70% | OS: 1.2 (0.7–1.9 ), *p* = 0.509 | MV |
| Kho 2012 c (40) | 404 | III | N | PT | quantitative | 70% | OS: 1.5 (1.0–2.3), *p* = 0.052 | MV |
| Boye 2016 (60) | 783 | II-III | C, N | PT | qualitative | moderate | RFS: 1.9 (1.3-2.9), *p* = 0.002 | MV |
| Kang 2011 a (39) | 526 | I-IV | C | PT | quantitative | 10% | OS: 1.59 (0.71–3.53), *p* = 0.258 | MV |
| Kang 2011 b (39) | 526 | I-IV | N | PT | quantitative | 10% | OS: 3.62 (1.06-12.38), *p* = 0.040 | MV |
| Huang 2011(42) | 112 | I-IV | C, N | PT+LN | score^b^ | 4 | OS: 2.88 (1.13-7.38), *p* = 0.027  RFS: 2.45 (1.05-5.70), *p* = 0.037 | MV |
| Sugai 2017 a (41) | 106 | II-IV | N | PT | quantitative | 11% | OS: 1.98 (0.84-4.58), *p* = 0.1145 | MV |
| Sugai 2017 b (41) | 106 | II-IV | S | PT | quantitative | 11% | OS: 6.37 (2.65-16), *p* = 0.0001 | MV |
| Boye 2010 (130) | 237 | I-III | C,N | PT | quantitative | 1% | OS: 1.6 (1.1-2.4), *p* = 0.02 | MV |
| Kwak 2010 (131) | 127 | I-IV | C,N | PT | quantitative | >10% | OS: 1.98 (0.95-4.13), *p* = 0.067 | MV |

S100A4 = Calcium-binding protein S100A4; OS = Overall survival; RFS = relapse free survival; MFS = metastasis free survival; *p*<0.05 = statistical significance;; HR = Hazard ratio; CI = Confidence Interval; C = cytoplasm; S = stromal; N = nuclear; LN=lymph nodes, N = nucleus; M= membrane; NS = Not specified; PT = primary tumor; CRLM = ColoRectal cancer Liver Metastasis; MV = multivariate analysis; UV = univariate analysis; quantitative = based on area of stained cells; qualitative = based on intensity of stained cells; Kho A = expression of S100A4 in cytoplasm at the tumor invasion front; Kho B = nuclear staining in central tumor; Kho C = nuclear expression at the tumor invasion front; Sugai A = S100A4 positive in tumor cells; Sugai B = S100A4 positive in interstitial space; score a= intesity + density, score b = intensity + extent of staining;

Table 11- Summary of the characteristics of studies included researching VIMENTIN expression

| **Study** | **Patients** | **Stage** | **Compartment** | **Type of tissue specimen** | **Determination of CAF expression in samples** | **Cut-off** | **Outcomes (HR [95% CI], *p)*** | **Analysis** |
| --- | --- | --- | --- | --- | --- | --- | --- | --- |
| Yun 2014 (74) | 409 | III | S | PT | score^a^ | 1 | DFS: 0.77 ( 0.42–1.41), *p* = 0.398  OS: 1.00 (0.51–1.97), *p* = 0.999 | UV |
| Wang 2017 (76) | 102 | I-III | C | PT | score^b^ | 3 | DFS: 1.41 (0.66-2.97), *p* = 0.369  OS: 1.05 (0.45-2.45), *p* = 0.900 | MV |
| Toiyama 2013(78) | 208 | I-III | S | PT | score^c^ | ≥2 | OS: 1.77 (0.84-3.71), *p* = 0.13 | UV |
| Lau 2016 (91) | 37 | IV | S | CRLM | quantitative | >3 | DFS: 0.92 (0.42-2.04), *p* = 0.851  OS: 0.32 (0.08-1.24), *p* = 0.101 | UV |
| Secinti 2022 (92) | 100 | I-IV | NS | PT | score^d^ | >3 | OS: 1.11 (0.88-1.40), *p* = 0.363 | UV |
| Liu 2017 (35) | 203 | II | S | PT | score^e^ | ≥6 | DFS: 2.03 (1.20-3.73), *p* = 0.022  OS: 2.02 (1.02-4.02), *p* = 0.043 | MV |
| Xiao 2015 (77) | 105 | I-IV | C | PT | score^f^ | ≥2 | OS: 1.573 (0.79-2.13), *p* = 0.084 | MV |
| Zasada 2022 (34) | 97 | I-III | S | PT | semi-quantitative | 6 | OS: 3.901 (2.43–6.24), *p* < 0.001 | MV |

OS = Overall survival; DFS = disease free survival; *p*<0.05 = statistical significance;; HR = Hazard ratio; CI = Confidence Interval; C = cytoplasm; S = stromal; N = nucleus; M= membrane; NS = Not specified; PT = primary tumor; CRLM = ColoRectal cancer Liver Metastasis; MV = multivariate analysis; UV = univariate analysis; quantitative = based on area of stained cells; qualitative = based on intensity of stained cells;

Score a–f: See Supplementary Materials 5 for full scoring criteria.

Table 12 - Summary of the characteristics of studies included researching CD 163 expression

| **Study** | **Patients** | | **Stage** | | **Compartment** | **Type of tissue specimen** | **Determination of CAF expression in samples** | **Cut-off** | **Outcomes (HR [95% CI], *p)*** | **Analysis** |
| --- | --- | --- | --- | --- | --- | --- | --- | --- | --- | --- |
| Liu 2021 (99) | 191 | | III | | M | PT | score^a^ | ≥4 | DFS: 1.01 (1.00-1.01), *p* = 0.003  OS: 1.00 (1.00-1.01), *p* = 0.049 | MV |
| Ledys 2018 a (100) | 114 | | IV | | M | CRLM | semi-quantitative | ++ | PFS: 1.10 (0.63-1.94), *p* = 0.73  OS: 0.719 (0.40-1.26), *p* = 0.25 | UV |
| Ledys 2018 b (100) | 114 | | IV | | M | CRLM | semi-quantitative | +++ | PFS: 1.196 (0.66-2.16), *p* = 0.56  OS: 0.80(0.44-1.46), *p* = 0.48 | UV |
| Cavalleri 2022 (101) | 236 | | III | | M | PT | quantitative | ≥0.93% | DFS: 3.15 (1.53–6.47), *p* = 0.002 | MV |
| Ye 2019 a (30) | 359 | | I-III | | N,C | PT | quantitative | Median | DFS: 1.01 (1.00-1.01), *p* = 0.001  OS: 4.33(1.45-12.93), *p* = 0.009 | MV |
| Ye 2019 b (30) | 249 | | I-III | | N,C | PT | quantitative | Median | DFS: 2.75 (1.61-4.69), *p* = 0.001  OS: 2.57 (1.20-5.46), *p* = 0.014 | MV |
| Ye 2019 c (30) | 400 | | I-III | | N,C | PT | quantitative | Median | DFS: 4.54 (2.49-8.28), *p* = 0,001  OS: 10.76 (3.06-37.77), *p* = 0.001 | MV |
| Xu 2021 (118) | 1021 | | I-III | | M,N | PT | quantitative | ≥181 | DFS: 1.70 (1.15–2.51), *p* = 0.05  OS: 2.10 (1.15–3.81), *p* < 0.05 | MV |
| Wang 2023 a (119) | 255 | | I-IV | | M | PT | quantitative | ≥ 10 | OS: 0.98 (0.95- 1.01), *p* = 0.192 | MV |
| Wang 2023 b (119) | 255 | | I-IV | | M | PT | quantitative | ≥ 35 | OS: 1.01 (1,01-1,02), *p* = < 0.001 | MV |
| Wen 2020 a (120) | 92 | | I-IV | | C,N,S | PT | qualitative | 1 | DFS: 0.71 (0.46–1.021), *p* = 0.320  OS: 0.96 (0.57–1.34), *p* = 0.465 | UV |
| Wen 2020 b (120) | 127 | | I-IV | | C,N,S | PT | qualitative | 1 | DFS: 0.69 (0.43–1.12), *p* = 0.608  OS: 0.88 (0.46–1.09), *p* = 0.442 | UV |
| Wei 2019 a (120) | 81 | | I-III | | S,M,N | PT | score^b^ | Median | RFS: 2.14 (1.01–4.52), *p* = 0.045  OS: 3.23 (1.17–8.89), *p* = 0.023 | MV |
| Wei 2019 b (120) | 81 | | I-III | | S,M,N | PT | score^b^ | Median | RFS: 1.23 (0.67–2.24), *p* = 0.498  OS: 1.91 (0.88–4.15), *p* = 0.100 | UV |
| Takasu 2021 (122) | 71 | | IV | | C,M | CRLM | quantitative | ≥ 20% | OS: 0.77 (0.21–2.85), *p* = 0.701 | MV |
| Shin 2021 (123) | 148 | | I-IV | | M | PT | quantitative | ≥21 | DFS: 0.99 (0.98–1.01), *p* = 0.848  OS: 1.002 (0.99–1.01), *p* = 0.747 | UV |
|  |  | |  | |  |  |  |  |  |  |
| **Study** | **Patients** | **Stage** | | **Compartment** | | **Type of tissue specimen** | **Determination of CAF expression in samples** | **Cut-off** | **Outcomes (HR [95% CI], *p)*** | **Analysis** |
| Shabo 2014 (124) | 75 | I-IV | | C | | PT | qualitative | ≥1 | OS: 1.6 (0.7-4.0), *p* = 0.211 | MV |
| Ozaki 2023 (125) | 205 | III | | C, S, N | | PT | quantitative | >40% | RFS: 1.38 (0.81-2.33), *p* = 0.232 | MV |
| Kitagawa 2022 (126) | 275 | I-III | | M | | PT | quantitative | 0.2 | RFS: 0.74 (0.43, 1.25), *p* = 0.266  OS: 0.70 (0.35, 1.35), *p* = 0.291 | UV |
| Edin 2012 (127) | 422 | I-IV | | M | | PT | semiquantitative | ≥2 | CSS: 0.66 (0.42–1.06), *p* = 0.087 | MV |
| Blom 2023 a (36) | 537 | I-IV | | M | | PT | semiquantitative | NS | OS: 1.91 (1.43 - 2.57), *p* < 0.001 | MV |
| Blom 2023 b (36) | 537 | I-IV | | M | | PT | semiquantitative | NS | OS: 1.72 (1.28 - 2.32), *p* < 0.001 | MV |
| Xue 2021 (128) | 209 | I-III | | M | | PT | quantitative | ≥115 | DFS: 0.19 (0.07–0.47), *p* < 0.001  OS: 0.18 (0.05–0.64), *p* = 0.008 | MV |
| Akter 2022 (132) | 399 | I-IV | | M | | PT | semiquantitative | ≥3 | RFS: 0.68 ( 0.35-1.30), *p* = 0.241 | UV |
| Ke 2023 (133) | 45 | I-IV | | M | | PT | quantitative | Median | OS: 0.29 (0.07-1.33), *p* = 0.112 | MV |
| Kanno 2020 a (134) | 117 | II-III | | M | | PT | quantitative | Median | DFS: 1.33 (0.58-3.12), *p* = 0.494  OS: 1.77 (0.43-8.63), *p* = 0.426 | UV |
| Kanno 2020 b (134) | 117 | II-III | | M | | PT | quantitative | Median | DFS: 1.33 (0.58-3.12), *p* = 0.494  OS: 1.27 (0.58-2.85), *p* = 0.542 | UV |

CD = cluster of differentiation; OS = Overall survival; DFS = disease free survival; RFS = relapse free survival; CSS = cancer specific survival; *p*<0.05 = statistical significance; HR = Hazard ratio; CI = Confidence Interval; C = cytoplasm; S = stromal,N = nucleus; M= membrane; NS = Not specified; PT = primary tumor; CRLM = ColoRectal cancer Liver Metastasis; MV = multivariate analysis; UV = univariate analysis; quantitative = based on area of stained cells; qualitative = based on intensity of stained cells; score = combination of intensity and percentage of stained cells; NS = Not specified; Wang a = CD163 intratumoral determination; Wang b= CD163 determination at invasion front; Wen a = patients with preoperative radiotherapy; Wen b = patients with surgery alone; Wei a = CD163 determination at the invasion front; Wei b = CD 163 determination at non invasion front; Blom a = expression in tumor; Blom b = expression in stroma; Kanno A = right sided tumor; Kanno B = left-sided tumor;

score a = proportion x intensity, score b = intensity + percentage, score c= immunoreactivity x intensity, scored 4; intensity of stain, score d = percentage of positive cells x staining intensity score; score e= intesity X percentage of stromal regions;

Table 13 - Summary of the characteristics of studies included researching TNC expression

| **Study** | **Patients** | **Stage** | **Compartment** | **Type of tissue specimen** | **Determination of CAF expression in samples** | **Cut-off** | **Outcomes (HR [95% CI], *p)*** | **Analysis** |
| --- | --- | --- | --- | --- | --- | --- | --- | --- |
| Hashimoto 2021 a (58) | 148 | II-III | C | PT | score | ≥2 | DFS: 2.31 (1.06 - 5.87), *p* = 0.0324  OS: 2.05 (0.84-6.13), *p* = 0.1201 | MV  UV |
| Hashimoto 2021 b (58) | 138 | II-III | C | PT | score | ≥2 | DFS: 3.97 (1.65-11.795), *p* = 0.0012  OS: 3.18 (1.038-13.882), *p* = 0.0421 | MV  UV |
| Murakami 2017 (90) | 139 | I-IV | S | PT | qualitative | ≥2 | OS: 3.41 (1.45–8.07), *p* = 0.005 | MV |
| Yang 2020 (105) | 100 | I-IV | N, C | PT | quantitative | NS | DFS: 2.23 (1.35-3.68), *p* < 0.002  OS: 2.19 (1.35-3.54), *p* < 0.001 | MV |
| Yang 2018 (106) | 100 | I-IV | S | PT | qualitative | ≥2 | OS: 2.86 (1.74-4.69), *p* < 0.001  DFS: 2.49 (1.51-4.12), *p* = <0.001 | MV |
| Ito 2023 (33) | 259 | II-III | S | PT | qualitative | ≥3 | DFS: 1.12 (0.66-1.90), *p* = 0.6647  OS: 2.16 (1.03 -4.52), *p* = 0.0412 | MV |

TNC = tenascin C; OS = Overall survival; DFS = disease free survival; CSS = cancer specific survival; *p*<0.05 = statistical significance;; HR = Hazard ratio; CI = Confidence Interval; C = cytoplasm; S = stromal; N = nucleus; M= membrane; NS = Not specified; PT = primary tumor; CRLM = ColoRectal cancer Liver Metastasis; MV = multivariate analysis; UV = univariate analysis; quantitative = based on area of stained cells; qualitative = based on intensity of stained cells; cells NS = Not specified; Hashimoto 2021 a = study cohort; Hashimoto 2021 b = validation cohort;

score a= Intensity x percentage of cells; score b= SI (staining intensity) × PP (percentage of positive cells);

Table 14 - Summary of the characteristics of studies included researching PDPN expression

| **Study** | **Patients** | **Stage** | **Compartment** | **Type of tissue specimen** | **Determination of CAF expression in samples** | **Cut-off** | **Outcomes (HR [95% CI], *p)*** | **Analysis** |
| --- | --- | --- | --- | --- | --- | --- | --- | --- |
| Yamanashi 2009 (46) | 120 | II-III | S | PT | quantitative | ≥30% | RFS: 0.15 (0.04-0.51), *p* = 0.0023  DSS: 0.16 (0.037-0.708), *p* = 0.0157 | MV |
| Cai 2019 (47) | 164 | I-IV | S | PT | quantitative | ≥10% | OS: 0.64 (0.37-1.12), *p* = 0.003 | MV |
| Algars 2011 (107) | 145 | II-IV | C | PT | qualitative | ++ | DSS: 1.09 (0.5-2.2), NS | UV |

PDPN = podoplanin; OS = Overall survival; DFS = disease free survival; RFS = relapse free survival; *p*<0.05 = statistical significance;; HR = Hazard ratio; CI = Confidence Interval; C = cytoplasm; S = stromal; N = nucleus; M= membrane; NS = Not specified; PT = primary tumor; CRLM = ColoRectal cancer Liver Metastasis; MV = multivariate analysis; UV = univariate analysis; quantitative = based on area of stained cells; qualitative = based on intensity of stained cells; score = combination of intensity and percentage of stained cells NS = Not specified;

score a = combination of intensity and extent of staining

Table 15 - Summary of the characteristics of studies included researching CXCL12 expression

| **Study** | **Patients** | **Stage** | **Compartment** | **Type of tissue specimen** | **Determination of CAF expression in samples** | **Cut-off** | **Outcomes (HR [95% CI], *p)*** | **Analysis** |
| --- | --- | --- | --- | --- | --- | --- | --- | --- |
| Yoshitake 2008 (104) | 60 | I-IV | C | PT | qualitative | Strong* | OS: 1.46 (0.52 – 4.10), *p* = 0.475 | MV |
| Fukasawa 2009 (43) | 165 | II-III | M, C | PT | quantitative | ≥50% | RFS: 4.08 (1.40-11.87 ), *p* = 0.010  OS: 4.13 (1.20-14.16), *p* = 0.024 | MV |
| D’Alterio 2014 (103) | 68 | II-III | M, C | PT | quantitative | ≥50% | RFS: 2.14 (0.87-5.27), *p* = 0.099  CSS: 2.38 (0.69-8.24), *p* = 0.171 | UV  MV |
| Stanisavljević 2015 a (29) | 263 | II-III | C | PT | quantitative | >10% | DFS: 5.13 (2.23 - 11.81), *p* < 0.001 | MV |
| Stanisavljević 2015 b (29) | 239 | I-III | C | PT | quantitative | >10% | DFS: 1.61 (0.95-2.73), *p* = 0.075 | MV |
| Stanisavljević 2015 c (29) | 96 | III | C | PT | quantitative | >10% | DFS: 3.25 (1.60 - 6.62), *p* = 0.001 | MV |
| Stanisavljević 2015 d (29) | 75 | III | C | PT | quantitative | >10% | DFS: 3.14 (1.04 to 9.44), *p* = 0.042 | MV |
| Zengin 2021 (45) | 260 | III-IV | C, M | PT | qualitative | ≥1 | RFS: 3.54 (1.52-4.67), *p* = 0.001  OS: 2.74 (1.48-4.71), *p* = 0.025 | MV |
| Okikawa 2021 (44) | 98 | I-IV | S, C | PT | qualitative | ≥2 | DFS: 2.07 (0.94-4.55), *p* = 0.06  OS: 2.85 (1.00-8.11), *p* = 0.04 | MV |
| Kim 2021 (37) | 121 | II-III | M | PT | semiquantitative | ≥2 | RFS: 6.62 (1.18-37.07), *p* = 0.031  OS: 2.23 (0.39-12.57), *p* = 0.363 | MV |

CXCL12 = C-X-C motif chemokine ligand 12; OS = Overall survival; DFS = disease free survival; RFS = relapse free survival; CSS = cancer specific survival;

*p*<0.05 = statistical significance;; HR = Hazard ratio; CI = Confidence Interval; C = cytoplasm; S = stromal; N = nucleus; M= membrane; NS = Not specified; PT = primary tumor; CRLM = ColoRectal cancer Liver Metastasis; MV = multivariate analysis; UV = univariate analysis; quantitative = based on area of stained cells; qualitative = based on intensity of stained cells; score = combination of intensity and percentage of stained cells; *Strong expression is greater than that of endothelial cells measured at the invasion front; Stanisavljević a= first cohort 1993-1996; Stanisavljević b = second cohort 2007-2011; Stanisavljević c = first cohort, stage III subgroup; Stanisavljević d = second cohort, stage III subgroup;

Table 16 - Summary of the characteristics of studies included researching MMP9 expression

| **Study** | **Patients** | **Stage** | **Compartment** | **Type of tissue specimen** | **Determination of CAF expression in samples** | **Cut-off** | **Outcomes (HR [95% CI], *p)*** | **Analysis** |
| --- | --- | --- | --- | --- | --- | --- | --- | --- |
| Unsal 2008 (93) | 60 | II-III | S | PT | score^a^ | ≥2 | DFS: 0.29 (0.09–0.86), *p* = 0.027  OS: 0.32 (0.10–0.98), *p* = 0.046 | MV  UV |
| Salem 2016 (64) | 127 | II-IV | C | PT | qualitative | ≥1 | DFS: 2.18 (1.14–4.18), *p* = 0.018 | MV |
| Peltonen 2020 a (94) | 111 | IV | C | PT | qualitative | ≥2 | DFS: 0.50 (0.29–0.86), *p* = 0.013  OS: 0.59 (0.33–1.05), *p* = 0.070 | UV |
| Peltonen 2020 b (94) | 111 | IV | C | CRLM | qualitative | ≥2 | DFS: 0.99 (0.57–1.71), *p* = 0.970  OS: 0.79 (0.43–1.45), *p* = 0.442 | UV |
| Yang 2017 (108) | 179 | I-III | C, M | PT | quantitative | ≥156 | PFS: 1.60 (0.79-3.25), *p* = 0.188  OS: 2.03 (0.87-4.72), *p* = 0.101 | MV |
| Ogata 2005 (109) | 307 | II-III | C | PT | quantitative | ≥10% | DFS: 2.34 (1.17–3.51), *p* < 0.001 | MV |
| Langers 2012 (110) | 198 | I-IV | N | PT | ELISA | ≥1,6 | OS: 1.948 (1.18-3.20), *p* = 0.009 | MV |
| Jensen 2010 a (67) | 340 | II-IV | C | PT | qualitative | ≥3 | RFS: 1.1 (0.6-1.9), *p* = 0.8  OS: 0.8 (0.4–1.6), *p* = 0.5 | MV |
| Jensen 2010 b (67) | 340 | II-IV | C | PT | qualitative | ≥3 | RFS: 0.7 (0.3-1.4), *p* = 0.3  OS: 0.4 (0.2–1.0), *p* = 0.1 | MV |
| Chu 2011 a (111) | 192 | II-IV | C | PT | qualitative | ≥1 | DFS: 2.65 (1.49–4.72), *p* = 0.001  OS: 3.41 (1.73–6.73), *p* < 0.001 | MV |
| Chu 2011 b (111) | 192 | II-IV | C | PT | qualitative | ≥5 | DFS: 4.07 (2.20–7.52), *p* < 0.001  OS: 5.44 (2.71–10.93), *p* < 0.001 | MV |
| Chu 2011 c (111) | 192 | II-IV | C | PT | qualitative | ≥9 | DFS: 7.46 (4.24–13.11), *p* < 0.001  OS: 10.02 (5.23–19.20), *p* < 0.001 | MV |
| Buhmeida 2009 (68) | 202 | II | S, C | PT | qualitative | ≥1 | DFS: 1.59 (1.02-2.45), *p* = 0.03 | MV |
| Araújo 2015 (112) | 180 | I-IV | C | PT | semiquanitative | ≥2 | OS: 1.12 (0.75 - 1.62), NS | MV |
| Wang 2019 (113) | 443 | I-IV | S, M | PT | qualitative | ≥6 | OS: 0.346 (0.24-0.48), *p* < 0.001 | MV |
| Bendardaf 2009 (114) | 359 | II-IV | S, C | PT | qualitative | ≥1 | OS: 0.636 (0.44-0.91), *p* = 0.014 | MV |
| Langenskiöld 2013 a (115) | 103 | I-III | S | PT | ELISA | NS* | CSS: 1.09 (0.99–1.20), *p* = 0.065 | MV |
| Langenskiöld 2013 b (115) | 103 | I-III | S | PT | ELISA | NS* | CSS: 1.01 (0.81–1.32), *p* = 0.767 | MV |

MMP = matrix metalloproteinase; OS = Overall survival; DFS = disease free survival; RFS = relapse free survival; CSS = cancer specific survival; *p*<0.05 = statistical significance; significance; HR = Hazard ratio; CI = Confidence Interval; C = cytoplasm; S = stromal; N = nucleus; M= membrane; NS = Not specified; PT = primary tumor; CRLM = ColoRectal cancer Liver Metastasis; MV = multivariate analysis; UV = univariate analysis; quantitative = based on area of stained cells; qualitative = based on intensity of stained cells; score = combination of intensity and percentage of stained cells; NS = Not specified; Peltonen 2020 a = Marker determined in primary tumor; Peltonen 2020 b = marker determined in the liver metastases; Jensen a = marker intratumoral cells; Jensen b = marker in immune infiltate at invasion front; Chu a = weak expression; Chu b = moderate expression; Chu c = strong expression; Langenskiöld a = patients with colon cancer; Langenskiöld b = patients with rectal cancer ; * cut-off selected based on the respective receiver operating characteristics (ROC) curve; score a = intensity x percentage of stained cells;
